# Global trends in testicular and prostate cancer among adolescents and young adult males aged 15–49 years, 1990–2021: insights from the GBD study

**DOI:** 10.1038/s41598-025-07361-3

**Published:** 2025-07-02

**Authors:** Xinyu Zhang, Yijia Li, Chaoguang Yan, Lanyue Ma, Mengjiao Yu, Yunchao Yang, Sen Lin, Ruiqi Zhao, Lisheng Peng

**Affiliations:** 1 The Fourth Clinical Medical College of Guangzhou University of Chinese Medicine, Guangzhou, China; 2 Weifang Traditional Chinese Hospital, Weifang, China; 3 Shandong College of Traditional Chinese Medicine, Yantai, Shandong China; 4 Shenzhen Traditional Chinese Medicine Hospital, Shenzhen, Guangdong China

**Keywords:** Global burden of disease, Adolescents and young adult males, Testicular cancer, Prostate cancer, Incidence, Disability-adjusted life years, Socio-demographic index, Estimated annual percentage change, Cancer epidemiology, Testicular cancer, Urological cancer

## Abstract

Malignant reproductive system tumors significantly impact the physical and mental health of adolescent and young adult males (AYAMs, aged 15–49 years), particularly their fertility. In recent years, the incidence of testicular and prostate cancer in this population has risen, while early diagnosis and treatment remain challenging. However, global epidemiological data on AYAMs are limited. This study aims to evaluate global trends in the incidence and burden of testicular and prostate cancers among AYAMs from 1990 to 2021, using data from the Global Burden of Disease (GBD) 2021 study, to address the research gap and inform public health strategies. We extracted data on the incidence and disability-adjusted life years (DALYs) for testicular and prostate cancer in adolescents and young adult males (AYAMs) aged 15–49 years from the 2021 Global Burden of Disease (GBD) database, covering 204 countries and territories from 1990 to 2021. As the GBD 2021 database only includes data for testicular cancer (ICD-10 code C62) and prostate cancer (ICD-10 code C61), other male reproductive system cancers, such as penile and epididymal cancer, were excluded. To analyze temporal trends, we applied the estimated annual percentage change (EAPC) method using linear regression models to calculate age-standardized incidence and DALY rates. Additionally, Spearman’s rank correlation was used to assess the relationship between age-standardized rates and the Socio-Demographic Index (SDI) for each country and region in 2021. In 2021, a total of 94,229 new cases of male reproductive system cancers were reported globally among AYAMs. From 1990 to 2021, the global age-standardized incidence rate significantly increased (EAPC = 1.38), while the age-standardized DALY rate exhibited a downward trend (EAPC = -0.26). Both testicular cancer and prostate cancer showed a consistent increase in age-standardized incidence rates, while their age-standardized DALY rates declined. Testicular cancer represented a significantly higher proportion of male reproductive system cancers in AYAMs globally compared to prostate cancer, with regional variations in the cancer burden. Additionally, the age-standardized incidence rates of both testicular and prostate cancers were positively correlated with the SDI, while the age-standardized DALY rate displayed a hill-shaped relationship with SDI. Over the past three decades, the incidence of testicular and prostate cancers among adolescents and young adult males (AYAMs) has steadily increased, accompanied by significant regional disparities in DALYs. To reduce the global burden of these cancers, particularly for AYAMs, targeted prevention strategies are crucial. These should include early screening, timely treatment, and public health education tailored to the unique needs of young males. In low- and middle-income countries, efforts should focus on improving health management, promoting physical activity, fostering healthy dietary habits, and enhancing access to early cancer screening for AYAMs. Early detection and intervention are essential for improving survival rates and minimizing the long-term effects of cancer, such as fertility issues and psychological impacts. Collaboration between governments, public health organizations, and social institutions is critical for advancing cancer prevention and control, with a focus on the specific health needs of AYAMs.

**Supplementary Information:**

The online version contains supplementary material available at 10.1038/s41598-025-07361-3.

## Introduction

Testicular and prostate cancers are significant global health concerns for men. With population growth and rapid societal development, the burden of these cancers has steadily increased, particularly among adolescent and young adult males (AYAMs) aged 15–49, where incidence rates show a notable upward trend^1^. Adolescents and young adults (15–49 years) experience crucial physical, emotional, and psychosocial development, often facing challenges like limited time for health check-ups, academic and work pressures, and unhealthy lifestyles^1–3^. Low health awareness can lead to delayed cancer diagnoses, missed treatments, and worse outcomes^4–6^. This is particularly critical during the prime reproductive years for men, as reproductive system cancers can significantly affect fertility^7^. Therefore, evaluating cancer burden and trends in this age group is essential for improving prognosis and shaping public health policies.

Testicular cancer is one of the most common malignancies among males aged 14–44^8,9^, has seen rising incidence in Western countries over the past two decades^10,11^. This cancer severely impacts patients’ quality of life, reproductive health, and imposes a long-term socioeconomic burden^12^. Advances in medical technology have made testicular cancer one of the most successfully treated malignancies, with multidisciplinary treatment approaches driving significant improvements^13^. Over the past two decades, the five-year survival rate for metastatic testicular cancer has increased from less than 30% to approximately 95%^14^. Despite these advancements, global disparities in survival rates remain stark. In well-resourced regions like Northern Europe, the incidence-to-mortality ratio for testicular cancer is 26:1, compared to just 2:1 in resource-limited areas such as Southeast Asia, South Asia, and Africa^15^. Prostate cancer is the most commonly diagnosed cancer in men across 112 countries and the leading cause of cancer-related deaths in men in 48 countries^16^. Its risk increases significantly with age, particularly after 60^16,17^. With global population aging and economic growth, the prostate cancer burden is expected to rise further^18^. Substantial disparities in incidence and mortality exist worldwide. Developed countries report higher incidence rates due to advanced screening, early diagnosis, and greater health awareness^19^. In contrast, mortality rates are disproportionately high among men of African descent, especially those in Afro-Caribbean, Sub-Saharan African, and African American populations^18,19^. Despite advances in screening and treatment, challenges persist in addressing survival disparities, ensuring early diagnosis, and improving equitable access to medical resources. To reduce preventable cancer deaths and address inequities among adolescent and young adult males aged 15–49, comprehensive epidemiological analyses of cancer burden trends in this group are essential.

The Global Burden of Diseases, Injuries, and Risk Factors Study provides a systematic evaluation of male reproductive system cancers burdens across 204 countries and territories, offering a unique perspective on trends over the past three decades^20^. The GBD 2021 database provides global epidemiological data for testicular and prostate cancer, while other male reproductive system cancers, such as penile and epididymal cancer, were excluded due to data limitations. While previous studies—such as Sung et al. (2021), which reported global incidence and mortality rates of testicular cancer, and Zhou et al. (2024), which conducted a comprehensive analysis of global cancer trends—have provided valuable population-level insights, they primarily focused on the general population and lacked targeted analyses of adolescents and young adult males (AYAM). Given the distinct disease patterns, behavioral risk profiles, and healthcare access challenges in the AYAM population, our study is the first to systematically assess trends in the incidence and disability-adjusted life years (DALYs) of testicular and prostate cancers among AYAM (aged 15–49 years), using data from the GBD 2021 study spanning 1990 to 2021. This work addresses a gap in the existing literature. In addition to assessing overall trends, our analysis also examined regional disparities across different Socio-Demographic Index (SDI) levels. Notably, we identified a marked increase in cancer burden among AYAM in low- and middle-SDI countries. These findings underscore the heterogeneity of public health challenges across development contexts and provide essential evidence to guide age-specific early screening programs, targeted health education, and more equitable resource allocation strategies aimed at improving male reproductive health globally.

## Methods

### Population definition

In this study, we analyzed data on male reproductive system cancers from the 2021 Global Burden of Diseases, Injuries, and Risk Factors Study. Although male reproductive system cancers include various types, such as prostate cancer, testicular cancer, penile cancer, and epididymal cancer, the GBD 2021 provides burden estimates only for two major cancers: testicular cancer and prostate cancer. According to the 10th edition of the International Classification of Diseases (ICD-10), published in 2019, testicular cancer is coded as C62 and prostate cancer as C61 (available on the official website: https://icd.who.int/browse10/2019/en).

In this study, adolescent and young adult males (AYAM) are defined as individuals aged 15 to 49 years, consistent with the age stratification used in the GBD framework to evaluate health burden across life stages. This range captures the epidemiological transition from post-puberty to early middle age. While some oncological studies define AYA populations as aged 15–29 or 15–39, the inclusion of prostate cancer—whose incidence increases markedly after age 40—necessitates extending the upper age limit to 49 years to better capture early-onset cases and relevant trends. This broader definition also improves sample size and statistical power. To address the inherent age heterogeneity within this group, subgroup analyses were conducted in 5-year intervals (15–19, 20–24, 25–29, 30–34, 35–39, 40–44, 45–49 years) to examine incidence and DALY trends across age strata and identify age-specific patterns of cancer burden within the AYAM population.

### Data source

The GBD 2021 project compiled data from cancer registries, cause-of-death surveillance systems, population-based surveys, and peer-reviewed literature across 204 countries and territories, with input from over 11,500 collaborators. It offers a comprehensive and systematic evaluation of the global burden of major diseases. In regions with limited or absent cancer registry data, the DisMod-MR 2.1 Bayesian meta-regression model was used to estimate disease burden by integrating temporal, spatial, and covariate data (e.g., smoking prevalence, healthcare access). Additional modeling tools, including Spatiotemporal Gaussian Process Regression (ST-GPR) and Cause of Death Ensemble Modeling (CODEm), were employed to improve estimate accuracy and consistency. All estimates are presented with 95% uncertainty intervals (UIs) to account for statistical uncertainty and variability in data quality. Bias correction procedures and data quality scoring were applied to minimize systematic errors. For this study, age-standardized incidence and disability-adjusted life year (DALY) data for testicular and prostate cancers in males aged 15–49 years from 1990 to 2021 were extracted using the GBD Results Tool (https://vizhub.healthdata.org/gbd-results/). Further details on data sources and modeling approaches are available in Supplementary File 1.

### SDI grouping

This study utilizes the Socio-Demographic Index (SDI) classification from GBD 2021 to categorize 204 countries and regions into five levels: low, low-middle, middle, high-middle, and high SDI, for subgroup analysis. SDI is a composite indicator of development, calculated as the geometric mean of the total fertility rate for women under 25, average years of schooling for individuals aged 15 and older, and per capita lagged distribution income, with values ranging from 0 (lowest) to 1 (highest)^21^.

### Statistical analysis

We calculated the age-standardized rate (ASR) per 100,000 population for the AYAMs population aged 15–49 using the formula^20^:

$$\:\text{ASR}=\frac{{\sum\:}_{i=1}^{k}\left({r}_{i}\times\:{w}_{i}\right)}{{\sum\:}_{i=1}^{k}{w}_{i}}\times\:\text{100,000}$$

($\:{r}_{i}$: the crude incidence or crude mortality rate for the *i*th age group, $\:{w}_{i}$: the standard population weight for the *i*-th age group, based on the World Standard Population provided by the World Health Organization (WHO), $\:k$: the number of age groups.)

We also calculated the estimated annual percentage change (EAPC) in the ASR to assess the average change trend over a specified time interval^22^.

$${\text{y}}=\alpha +\beta {\text{x}}+\varepsilon ;\,\,\,{\text{EAPC}}={\text{1}}00 \times ({\text{exp(}}\beta - {\text{1))}}$$

(y: the natural logarithm of ASR, x: corresponding calendar year)

If EAPC > 0, it indicates an annual increase (upward trend); if EAPC < 0, it indicates an annual decrease (downward trend). The absolute value of EAPC reflects the rate of annual change.

To validate the robustness of the estimated EAPC, we performed model diagnostics, including residual analysis and goodness-of-fit tests, to ensure compliance with linear regression assumptions. Additionally, stratified analyses were conducted based on SDI levels, regional types, and age groups. The results demonstrated consistent EAPC trends across all subsets, confirming the robustness and reliability of the model.

We used a local regression smoothing model (loess) and the “geom_smooth” function in ggplot2 to analyze the relationship between cancer burden in AYAMs and SDI across 21 regions and 204 countries and territories. Despite smaller sample sizes in some countries or regions, the GBD database provides reliable and accurate data based on model inference, considering factors such as population structure and health status. This enabled effective analysis using aggregated global and regional data. Additionally, Spearman’s correlation analysis was performed to calculate the r coefficient and p-value for the relationship between the burden and SDI in 2021. Statistical significance was defined as *p* < 0.05. All statistical analyses and graphical representations were conducted using R software (version 4.4.1).

### Ethics statement

For the GBD study, the Institutional Review Board of the University of Washington reviewed and approved a waiver of informed consent. This work has been reported in accordance with the STROCSS guidelines.

## Results

### Global trends

In 2021, the global incidence of cancer among adolescent and young adult males (AYAMs) reached 94,229 cases, with an age-standardized incidence rate of 4.66 per 100,000 population. The total disability-adjusted life years (DALYs) were approximately 607,131, with an age-standardized DALY rate of 30.06 per 100,000 population (Table 1). Testicular cancer accounted for 76,364 new cases and prostate cancer for 17,865, with age-standardized incidence rates of 3.81 and 0.85 per 100,000, respectively. The corresponding DALYs were 461,366 (23.07 per 100,000) for testicular cancer and 145,764 (6.99 per 100,000) for prostate cancer (Table 1).

**Table 1:** Incidence and dalys of male reproductive system cancers in adolescent and young adult males (AYAMs) in 1990 and 2021, and their estimated annual percentage changes from 1990 to 2021.

Characteristics	Incidence					DALYs				
	Cases, 1990	ASR per 100,000, 1990	Cases, 2021	ASR per 100,000, 2021	EAPC, 1990–2021	Cases, 1990	ASR per 100,000, 1990	Cases, 2021	ASR per 100,000, 2021	EAPC, 1990–2021
Global	40,426 (38457–42217)	3.07 (2.87–3.26)	94,229 (88,911–99,404)	4.66 (4.30–5.03)	1.38 (1.29–1.46)	422,010 (381,303–453,363)	32.00 (28.59–34.86)	607,131 (549142–657967)	30.06 (26.87–33.05)	− 0.26 (− 0.35 to − 0.16)
Causes										
Testicular cancer	33,858 (32686–35163)	2.49 (2.36–2.63)	76,364 (73294–79921)	3.81 (3.57–4.09)	1.5 (1.43–1.56)	335,128 (314,206–355,842)	24.52 (22.80–26.39)	461,366 (433067–493203)	23.07 (21.38–25.00)	− 0.18 (− 0.31 to − 0.04)
Prostate cancer	6568 (5771–7053)	0.58 (0.51–0.63)	17,865 (15617–19483)	0.85 (0.73–0.94)	0.9 (0.59–1.21)	86,882 (67,096–97,521)	7.48 (5.80–8.47)	145,764 (116075–164764)	6.99 (5.49–8.05)	− 0.5 (− 0.62 to − 0.38)
GBD regions										
Andean Latin America	161 (119–212)	1.90 (1.26–2.84)	1042 (780–1372)	5.94 (3.91–8.76)	3.82 (3.35–4.3)	4401 (3221–5754)	50.68 (33.87–74.27)	10,012 (7662–12844)	57.05 (39.91–79.77)	0.36 (− 0.03 to 0.75)
Australasia	736 (647–841)	13.51 (10.10–17.77)	1341 (1106–1598)	17.66 (12.22–24.45)	0.74 (0.24–1.25)	2957 (2652–3270)	54.29 (44.22–65.66)	2531 (2092–3087)	33.08 (24.56–44.75)	− 1.75 (− 1.99 to − 1.51)
Caribbean	134 (116–155)	1.78 (1.46–2.14)	499 (407–607)	4.11 (3.15–5.31)	2.4 (1.94–2.86)	1776 (1555–2068)	23.19 (19.28–28.03)	4288 (3470–5250)	35.37 (27.32–45.58)	1.15 (0.81 to 1.49)
Central Asia	316 (270–378)	2.06 (1.71–2.52)	767 (654–916)	3.06 (2.53–3.72)	1.28 (0.95–1.61)	6152 (5368–7104)	40.04 (34.60–46.59)	9642 (8254–11373)	38.47 (32.62–45.87)	− 0.26 (− 0.39 to − 0.12)
Central Europe	2412 (2232–2650)	7.66 (6.79–8.70)	4492 (4026–5036)	16.49 (14.04–19.34)	2.81 (2.67–2.95)	27,672 (25,987–29,766)	88.00 (81.29–95.95)	19,652 (17702–21799)	69.89 (62.15–78.46)	− 0.55 (− 0.69 to − 0.42)
Central Latin America	1185 (1116–1254)	3.22 (2.95–3.53)	7234 (6487–8017)	11.12 (9.72–12.68)	4.03 (3.86–4.21)	23,554 (22,581–24,541)	60.56 (56.77–64.66)	63,502 (57521–69608)	97.57 (87.12–108.54)	1.72 (1.58 to 1.87)
Central Sub-Saharan Africa	70 (44–95)	0.72 (0.41–1.13)	317 (201–458)	1.14 (0.66–1.83)	1.56 (1.31–1.82)	2661 (1684–3631)	26.63 (14.97–41.75)	8755 (5583–12571)	30.94 (17.45–50.20)	0.6 (0.44 to 0.75)
East Asia	2252 (1634–2777)	0.68 (0.47–0.87)	7049 (5378–9014)	1.79 (1.30–2.40)	3.11 (2.85–3.36)	59,962 (43,038–74,084)	17.73 (12.23–22.76)	48,967 (36164–62789)	12.52 (9.04–16.38)	− 1.61 (− 1.91 to − 1.31)
Eastern Europe	2435 (2245–2616)	4.40 (3.97–4.91)	4715 (4284–5132)	8.86 (7.88–9.88)	2.09 (1.88–2.3)	28,887 (26,270–31,308)	52.34 (46.44–58.89)	26,851 (23600–30118)	50.52 (43.54–58.81)	− 0.64 (− 0.93 to − 0.35)
Eastern Sub-Saharan Africa	272 (182–357)	0.82 (0.52–1.14)	1331 (987–1701)	1.52 (1.05–2.04)	1.98 (1.78–2.18)	10,223 (6781–13,674)	29.85 (18.75–41.19)	35,028 (25806–44951)	39.15 (27.32–52.50)	0.9 (0.77 to 1.03)
High-income Asia Pacific	2266 (2004–2576)	4.94 (3.80–6.40)	2597 (2309–2899)	6.38 (4.97–7.85)	0.55 (0.04–1.06)	9943 (9205–10,695)	21.42 (19.40–23.83)	5697 (5064–6478)	13.27 (11.46–15.67)	− 1.84 (− 2.13 to − 1.55)
High-income North America	8948 (8590–9346)	11.74 (10.93–12.64)	15,096 (14290–15995)	17.21 (15.77–18.85)	1.11 (0.96–1.26)	32,011 (30,256–34,268)	42.07 (39.34–45.33)	34,569 (31472–38189)	39.39 (35.52–43.97)	− 0.29 (− 0.4 to − 0.18)
North Africa and Middle East	1801 (1366–2296)	2.29 (1.48–3.47)	12,290 (10236–14507)	7.00 (5.19–9.52)	4.13 (3.86–4.39)	14,373 (10,856–17,916)	18.92 (12.08–27.92)	31,916 (25494–38077)	18.08 (13.26–23.46)	0.14 (− 0.08 to 0.35)
Oceania	12 (8–17)	0.83 (0.51–1.29)	35 (23–49)	1.05 (0.63–1.65)	0.78 (0.7–0.86)	318 (208–456)	22.31 (13.31–35.31)	823 (533–1190)	24.61 (14.30–40.28)	0.43 (0.34 to 0.53)
South Asia	2394 (1949–2827)	0.92 (0.73–1.13)	8266 (7066–9703)	1.63 (1.34–1.99)	1.89 (1.62–2.15)	80,364 (64,718–95,223)	30.59 (24.00–37.78)	143,529 (121576–168171)	28.33 (23.14–34.99)	− 0.26 (− 0.38 to − 0.13)
Southeast Asia	882 (718–1034)	0.84 (0.63–1.04)	3504 (2653–4346)	1.84 (1.33–2.39)	2.44 (2.38–2.51)	21,449 (17,579–25,018)	20.23 (15.56–24.68)	44,889 (33893–57368)	23.49 (17.20–30.69)	0.4 (0.34 to 0.45)
Southern Latin America	850 (719–998)	7.09 (5.40–9.22)	3450 (2963–4021)	19.77 (14.98–25.75)	3.47 (3.12–3.83)	13,188 (11,455–15,056)	110.18 (87.14–138.42)	19,360 (16927–21851)	110.76 (88.69–135.41)	0.23 (0.03 to 0.42)
Southern Sub-Saharan Africa	153 (122–186)	1.51 (1.16–1.95)	513 (414–675)	2.55 (1.96–3.48)	1.83 (1.69–1.96)	4004 (3197–4890)	39.31 (30.27–50.30)	9550 (7662–12141)	47.36 (36.13–63.34)	0.67 (0.51 to 0.84)
Tropical Latin America	725 (672–785)	2.04 (1.82–2.31)	3493 (3162–3821)	5.81 (5.03–6.70)	3.34 (3.21–3.48)	15,202 (14,247–16,119)	42.15 (38.13–46.52)	34,916 (32185–37421)	58.10 (51.86–64.89)	1.07 (1.01 to 1.13)
Western Europe	12,185 (11564–12938)	12.24 (10.99–13.66)	15,248 (14057–16649)	15.31 (13.28–17.71)	1.03 (0.74–1.31)	54,899 (51,805–58,437)	55.00 (50.92–59.88)	30,155 (26680–34409)	29.55 (25.41–34.77)	− 1.71 (− 1.82 to − 1.61)
Western Sub-Saharan Africa	238 (164–305)	0.68 (0.45–0.90)	950 (578–1290)	1.12 (0.65–1.55)	1.61 (1.48–1.73)	8016 (5552–10227)	22.52 (14.94–29.89)	22,500 (13936–29997)	25.98 (15.25–35.21)	0.43 (0.33 to 0.54)

From 1990 to 2021, the global age-standardized incidence rate of cancer among adolescent and young adult males (AYAMs) increased (EAPC = 1.38, 95% CI: 1.29 to 1.46), while the age-standardized DALY rate declined (EAPC = − 0.26, 95% CI: − 0.35 to − 0.16) (Table 1). Both testicular cancer (EAPC = 1.5, 95% CI: 1.43 to 1.56) and prostate cancer (EAPC = 0.9, 95% CI: 0.59 to 1.21) showed a significant rise in incidence rates. However, their age-standardized DALY rates decreased, with testicular cancer (EAPC = − 0.18, 95% CI: − 0.31 to − 0.04) and prostate cancer (EAPC = − 0.50, 95% CI: − 0.62 to − 0.38) exhibiting a downward trend (Table 1).

### Regional disparities

**Fig. 1:**
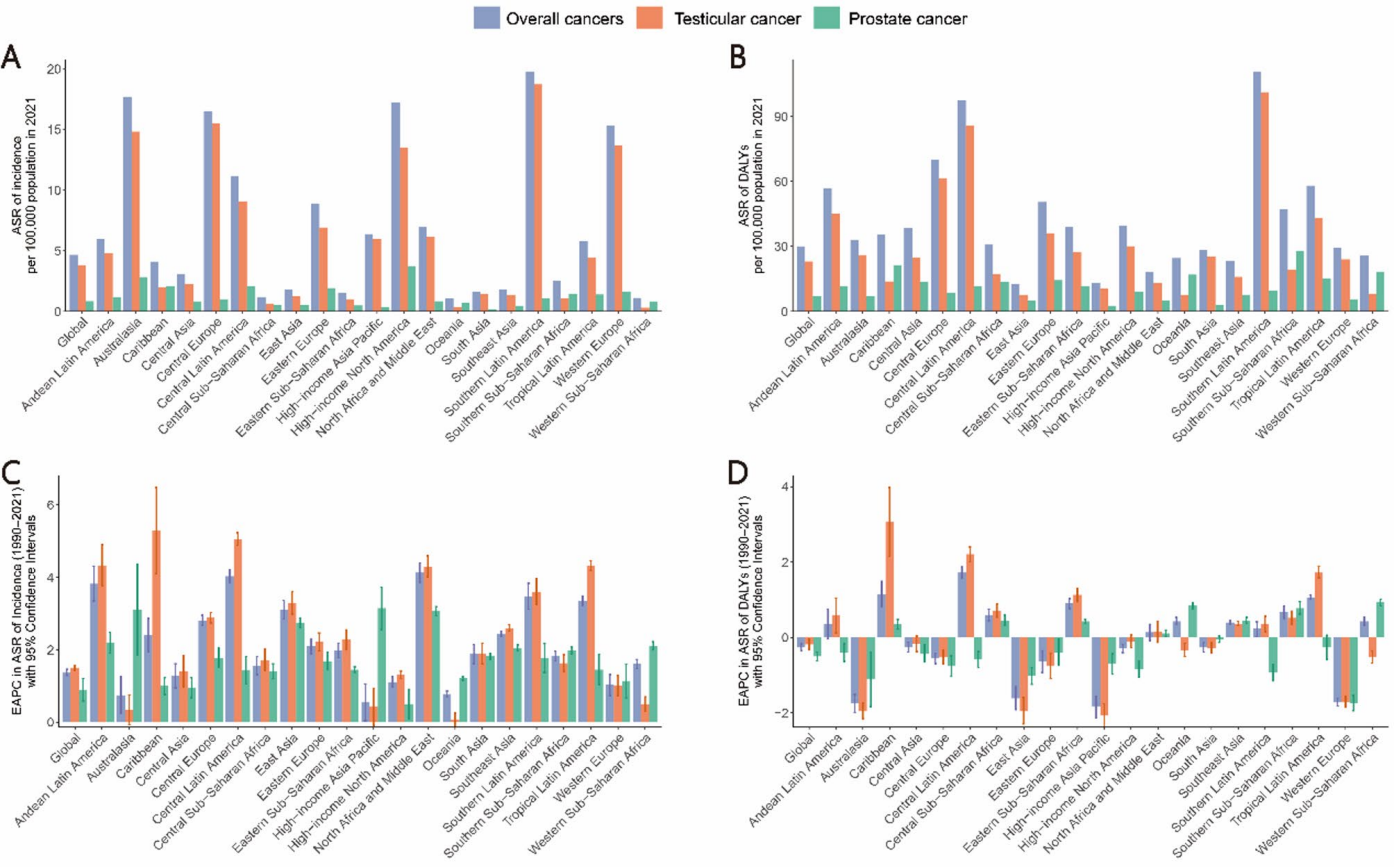
Age-standardized incidence and DALY rates in 2021, and their estimated annual percentage changes from 1990 to 2021 for male reproductive system cancers in adolescent and young adult males (AYAMs), globally and by 21 GBD regions. Age-standardized rates of incidence (**A**) and DALYs (**B**) and estimated annual percentage changes of age-standardized rates of incidence (**C**) and DALYs (**D**). Male reproductive system cancers include testicular and prostate cancers. *DALY* disability-adjusted life-years, *EAPC* estimated annual percentage change, *ASR* age-standardized rate.

**Fig. 2:**
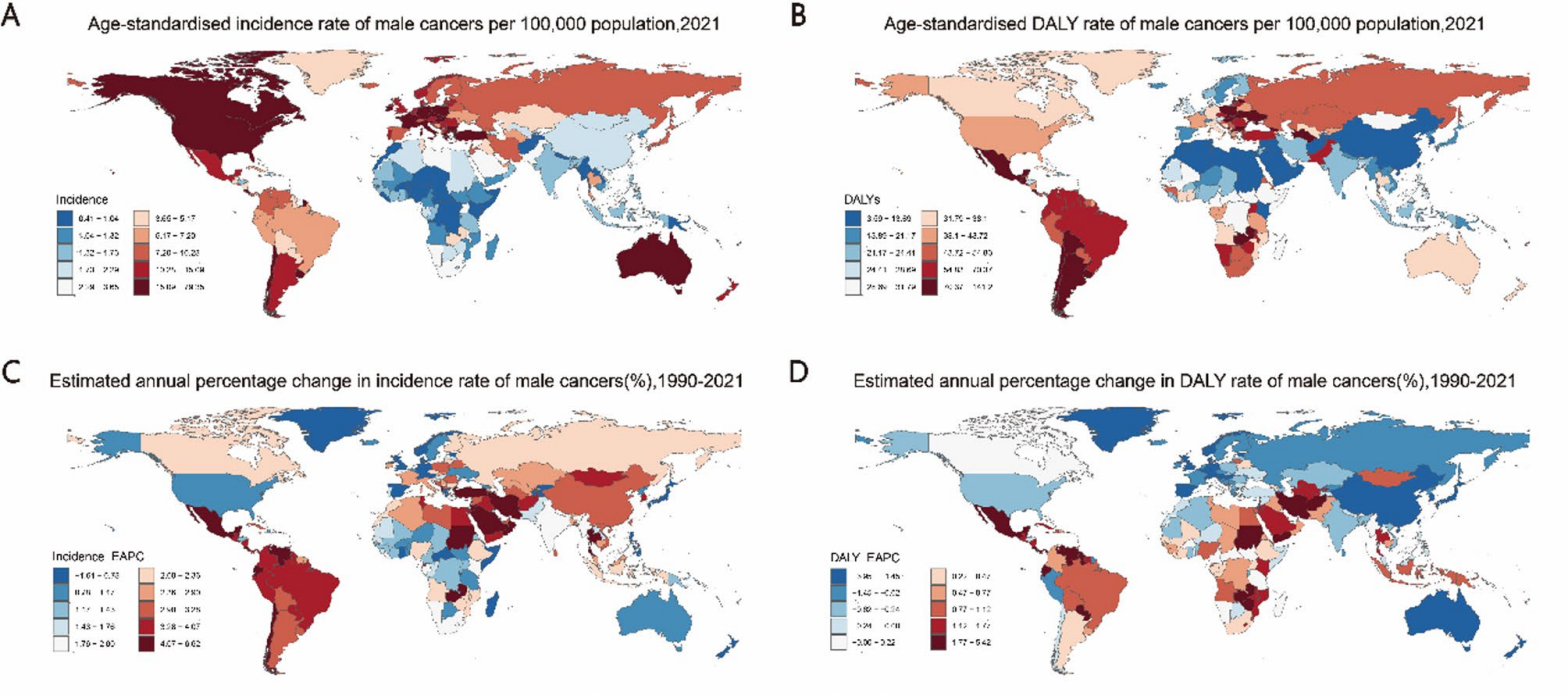
National age-standardized incidence and DALY rates in 2021, and their estimated annual percentage changes from 1990 to 2021 for overall male reproductive system cancers in adolescent and young adult males (AYAMs). Age-standardized rates of incidence (**A**) and DALYs (**B**). Estimated annual percentage changes of age-standardized incidence rate (**C**) and DALY rate (**D**). Male reproductive system cancers include testicular and prostate cancers. DALY, disability-adjusted life-years.

**Fig. 3:**
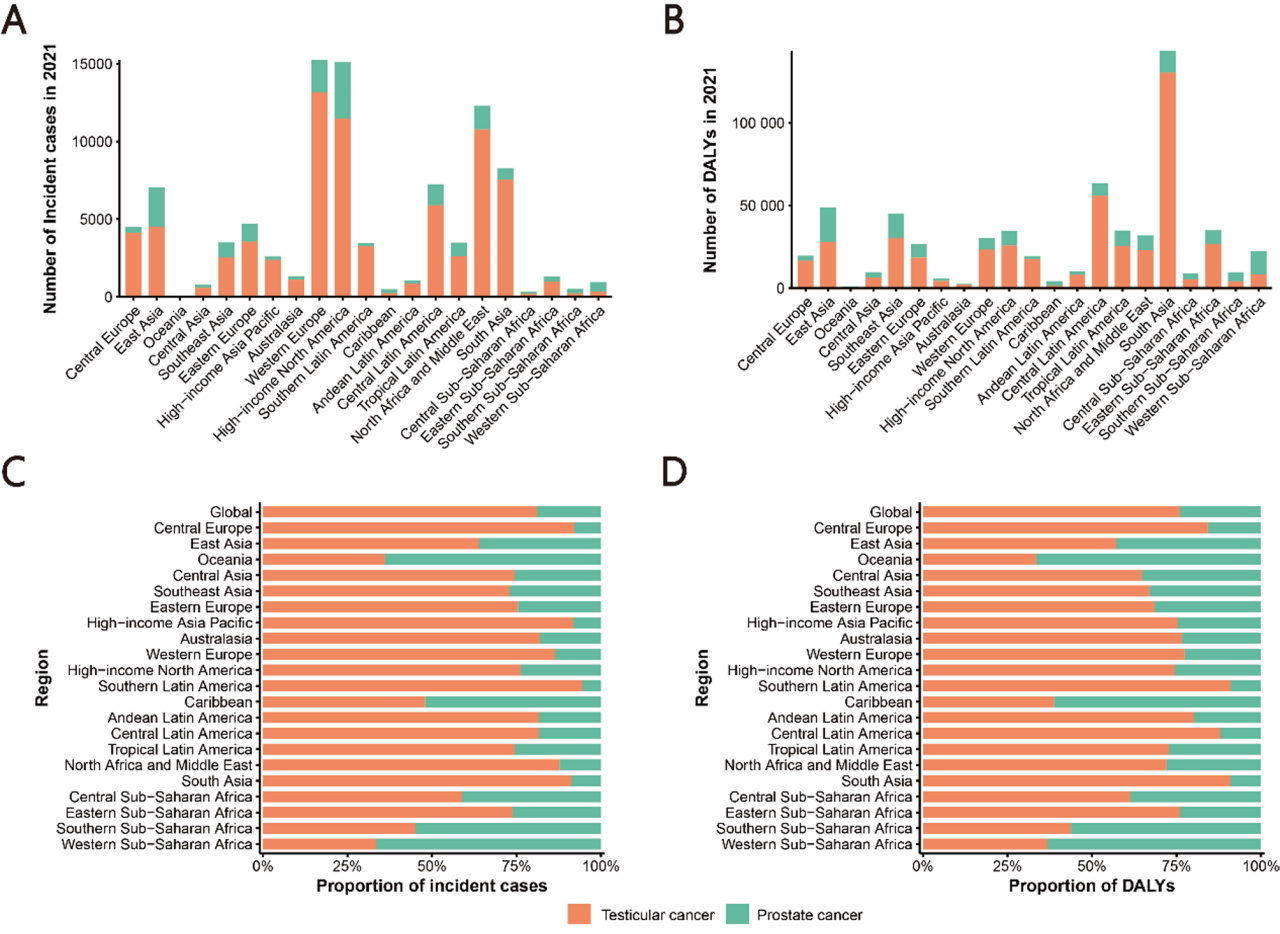
Numbers and proportions of incident cases and DALYs contributed by 21 GBD regions, for male reproductive system cancers in adolescent and young adult males (AYAMs), in 2021. Numbers of incident cases (**A**) and DALYs (**B**) of each cancer. Proportions of incident cases (**C**) and DALYs (**D**) accounted for by each cancer. Male reproductive system cancers include testicular and prostate cancers. DALY, disability-adjusted life-years.

In 2021, among the 21 GBD regions and 204 countries and territories, the highest age-standardized incidence rates of male reproductive system cancers were observed in Southern Latin America (19.77 per 100,000) and Monaco (79.35 per 100,000), while the highest age-standardized DALY rates were reported in Southern Latin America (110.76 per 100,000) and Monaco (141.23 per 100,000) (Table 1, Table S1, Figs. 1A,B and 2A,B). The highest number of new testicular and prostate cancer cases was recorded in Western Europe, high-income North America, and North Africa and the Middle East, whereas South Asia had the highest DALY burden for both cancers (Fig. 3A,B). Testicular cancer accounted for a larger proportion of global male reproductive system cancer cases (81.04% of incidence and 75.99% of DALYs) compared to prostate cancer (18.96% and 24.01%, respectively) (Fig. 3C,D).

Southern Latin America had the highest age-standardized incidence rate of testicular cancer (18.72 per 100,000), while high-income North America reported the highest incidence rate of prostate cancer (3.75 per 100,000). The highest age-standardized DALY rates for testicular and prostate cancer were observed in Southern Latin America (101.27 per 100,000) and Southern Sub-Saharan Africa (27.97 per 100,000), respectively. Western Sub-Saharan Africa (0.31) and South Asia (0.16) had the lowest age-standardized incidence rates of testicular and prostate cancer, while Oceania (7.61) and High-Income Asia Pacific (2.55) had the lowest age-standardized DALY rates (Tables S2, S4, Fig. 1A,B).

From 1990 to 2021, at the regional and national levels, the most rapid increase in age-standardized incidence rates of male reproductive system cancers was observed in North Africa and the Middle East (EAPC = 4.13, 95% CI: 3.86 to 4.39) and Zambia (EAPC = 6.63, 95% CI: 5.58 to 7.69). While most regions experienced a significant decline in age-standardized DALY rates, Central Latin America showed the most notable increase (EAPC = 1.72, 95% CI: 1.58 to 1.87). Among countries, Zambia recorded the highest rise in age-standardized DALY rates (EAPC = 5.43, 95% CI: 4.53 to 6.33) (Table 1, Table S1, Figs. 1C,D, 2C,D).

The age-standardized incidence rates of testicular and prostate cancer exhibited an upward trend across all regions. The Caribbean recorded the largest increase in testicular cancer incidence (EAPC = 5.29, 95% CI: 4.10–6.48), while the highest rise in prostate cancer incidence was observed in High-income Asia Pacific (EAPC = 3.15, 95% CI: 2.56–3.73). In contrast, the lowest increase in testicular cancer incidence was reported in Oceania (EAPC = 0.06, 95% CI: −0.14–0.25), whereas North America, High-income, had the smallest increase in prostate cancer incidence (EAPC = 0.06, 95% CI: 0.09–0.91). Regarding DALYs, the age-standardized DALY rate of testicular cancer increased in 10 regions, with the most pronounced rise observed in the Caribbean (EAPC = 3.06, 95% CI: 2.15–3.98), while the greatest decline occurred in High-income Asia Pacific (EAPC = −2.07, 95% CI: −2.38 to −1.76). The age-standardized DALY rate of prostate cancer increased in 8 regions, with the highest growth reported in Western Sub-Saharan Africa (EAPC = 0.93, 95% CI: 0.84–1.02) and the largest decline observed in Western Europe (EAPC = −1.74, 95% CI: −1.94 to −1.54) (Tables S2, S4; Fig. 1C,D). Detailed country-level trends are provided in Supplementary material (Tables S3 and S5; Figs. S1 and S2).

### Age-group disparities in the burden of two male reproductive system cancers

**Fig. 4:**
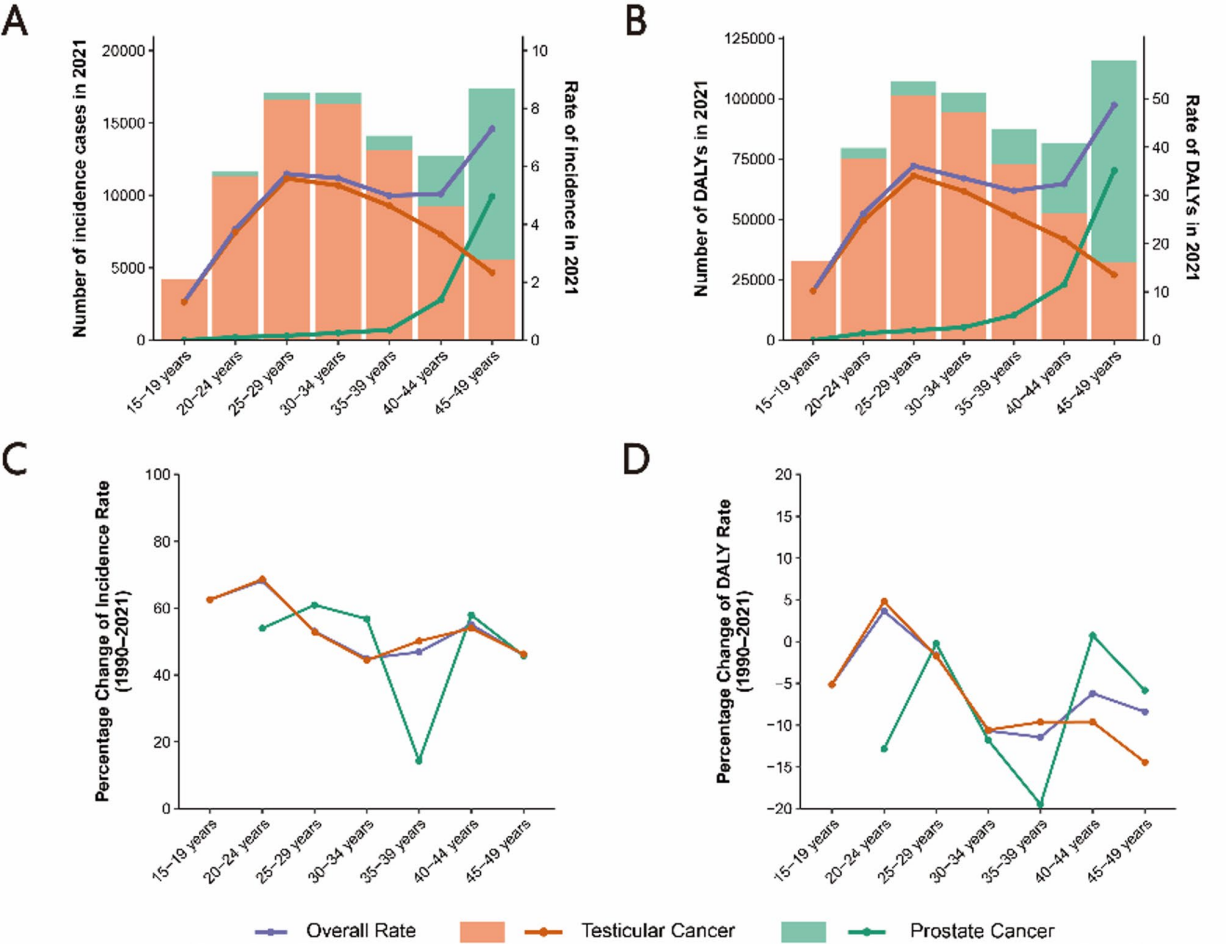
The cross-sectional (2021) and longitudinal trends (1990–2021) of incidence rate and DALY rate of male reproductive system cancers throughout adolescent and young adult males. Numbers and rates of incident cases (**A**) and DALYs (**B**) of male reproductive system cancers. Percentage changes of incidence rate (**C**) and DALY rate (**D**) of male reproductive system cancers. Male reproductive system cancers include testicular and prostate cancers. DALY, disability-adjusted life-years.

In 2021, the incidence and DALYs for testicular cancer among AYAMs peaked at ages 25–29 before declining with increasing age, whereas prostate cancer incidence and DALYs increased with age, peaking at 45–49 years (Fig. 4A,B). This age distribution pattern aligns with previous research: testicular cancer incidence peaks in early young adulthood, likely associated with post-pubertal germ cell mutations. In contrast, prostate cancer tends to manifest at an earlier age in some high-SDI regions, potentially reflecting increased health awareness and the widespread implementation of early PSA screening. Between 1990 and 2021, the incidence of both cancers increased across all age groups. The most significant increases in incidence and DALY rates for testicular cancer occurred in the 20–24 age group, while prostate cancer saw the most notable increases in the 25–29 and 40–44 age groups (Fig. 4C,D).

### The association between ASR, EAPC, and SDI

**Fig. 5:**
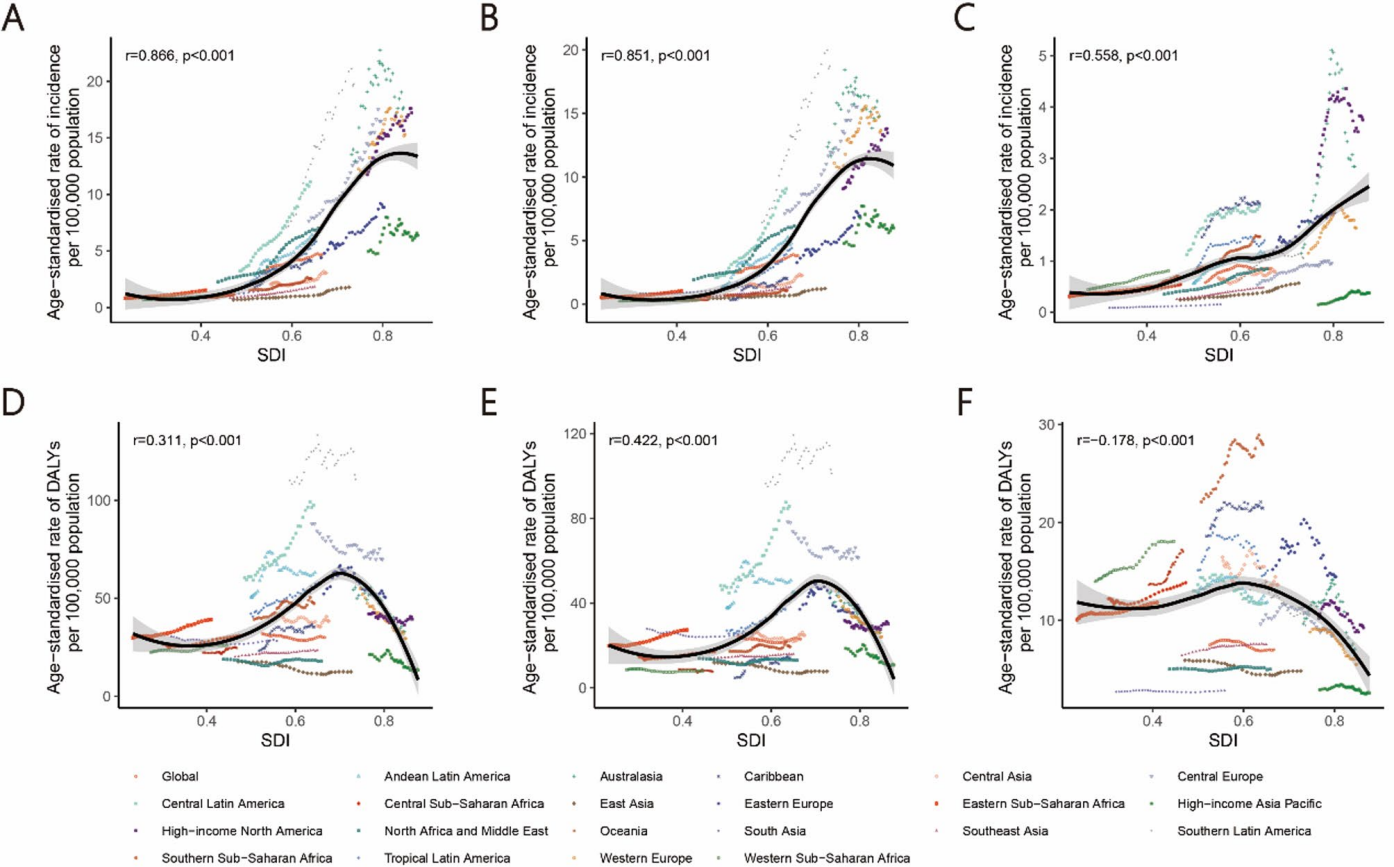
Age-standardized rates of incidence and DALYs of male reproductive system cancers in adolescent and young adult males (AYAMs), globally and for 21 GBD regions, by SDI (2021), from 1990 to 2021. Age-standardized incidence rates of overall male reproductive system cancers (**A**), testicular cancer (**B**), prostate cancer (**C**), by SDI. Age-standardized DALY rates of overall male reproductive system cancers (**D**), testicular cancer (**E**), and prostate cancer (**F**), by SDI. Expected values with 95% CI, based on SDI and disease rates in all locations, are shown as a solid line and shaded area; 32 points are plotted for each region and show the observed age-standardized incidence or DALY rates for each year from 1990 to 2021. Points above the solid line represent a higher-than-expected burden, and those below the line show a lower-than-expected burden. Male reproductive system cancers include testicular and prostate cancers. *DALY* disability-adjusted life-years, *GBD* global burden of diseases, injuries, and risk factors study. *SDI* socio-demographic index.

From 1990 to 2021, across the 21 regions, the overall age-standardized incidence rates of testicular and prostate cancers increased with rising SDI(*r* = 0.866, *p* < 0.01). In contrast, the age-standardized DALY rates for both cancers initially increased with SDI but began to decline at approximately SDI 0.7 for testicular cancer and SDI 0.6 for prostate cancer (Fig. 5).

The 2021 SDI reflected healthcare levels and resource availability across countries. In 2021, the overall age-standardized incidence rate of male reproductive system cancers increased with rising SDI across 204 countries and territories (*r* = 0.549, *p* < 0.01), with testicular (*r* = 0.504, *p* < 0.01) and prostate cancers (*r* = 0.449, *p* < 0.01) following similar trends. Conversely, the overall age-standardized DALY rate of male reproductive system cancers initially rose with increasing SDI but began to decline at approximately SDI 0.7, with testicular and prostate cancers showing comparable patterns (Figure S3–S5).

From 1990 to 2021, countries and territories with middle and high-middle SDI experienced a more rapid increase in the age-standardized incidence rates of overall, testicular, and prostate cancers. Conversely, those with high-middle and high SDI exhibited a more pronounced decline in age-standardized DALY rates for these cancers (Figure S3–S5).

### Data quality

This study used data from the GBD 2021 database, covering 204 countries and territories from 1990 to 2021. Data sources include cancer registries, hospital records, mortality databases, and published literature, ensuring comprehensive coverage of testicular and prostate cancers in adolescent and young adult males. To ensure comparability across regions and time periods, the GBD study employed a standardized modeling framework combining Bayesian meta-regression (DisMod-MR 2.1) and spatiotemporal Gaussian process regression. Age-standardized incidence rates (ASIR) and disability-adjusted life years (DALYs) were calculated using the WHO’s World Standard Population. Despite some regional data limitations, rigorous uncertainty quantification (95% UI) and cross-validation with external sources enhance the reliability of the estimates. While potential biases exist due to missing or variable national data, the GBD methodology mitigates these issues through the integration of multiple data sources and advanced statistical modeling.

## Discussion

This study systematically assessed the global incidence and DALYs of testicular and prostate cancer among adolescent and young adult males (AYAMs), analyzing long-term trends. Key findings include: (1) Between 1990 and 2021, age-standardized incidence of testicular and prostate cancer increased, while age-standardized DALY rates decreased; (2) Testicular cancer has higher incidence and DALY rates than prostate cancer, with notable regional variations; (3) As SDI increases, both testicular and prostate cancer incidence rise, while DALY rates initially increase and then decline; (4) Testicular cancer incidence declines after peaking at ages 25–29, whereas prostate cancer incidence continues to rise with age.

Our results show that from 1990 to 2021, the age-standardized incidence rates of male reproductive system cancers among AYAMs have been rising globally, a finding consistent with previous studies that highlight the growing cancer burden in this population. This study did not directly assess the role of genetic susceptibility in male reproductive system cancers. However, previous research indicates that genetic factors contribute substantially to their etiology^23^, accounting for approximately 50% of the overall risk. Individuals with a family history of testicular or prostate cancer, or those harboring relevant gene mutations, are at significantly elevated risk for disease development^24–26^. Testicular cancer incidence is notably higher in men of European descent, particularly in regions such as Southern Latin America (which has a large European immigrant population), Central Europe, and Western Europe, where the pattern of testicular cancer incidence aligns with genetic predisposition^27,28^. Prostate cancer genetic risk is especially pronounced among African descent populations, where the incidence and mortality rates for prostate cancer are significantly higher than in other racial groups^29^. Studies in the U.S. show that African American men have a prostate cancer incidence rate 1.7 times that of white men, with a lower survival rate^30,31^. Lifestyle factors play a significant role in the incidence patterns of male reproductive system cancers. Unhealthy habits, such as high-fat diets, excessive consumption of red meat and dairy products, and smoking, are closely associated with an increased risk of testicular and prostate cancers^32–34^. For testicular cancer, one of the major risk factors is cryptorchidism, with individuals remaining at higher risk even after corrective surgery^12,35^. Obesity is a known risk factor for prostate cancer, particularly for aggressive or advanced forms of the disease^36^. In contrast, regular moderate to high-intensity physical exercise has been shown to significantly reduce the risk of developing aggressive prostate cancer^37^. Environmental pollutants, particularly endocrine-disrupting chemicals (EDCs) and industrial chemicals, including polybrominated diphenyl ethers (PBDEs), phthalates, and heavy metals like lead, cadmium, and mercury, have been shown to be closely associated with increased incidence rates of testicular and prostate cancers^38–41^. EDCs are commonly found in everyday consumer products such as plastics, cleaning agents, cosmetics, and pesticides, and can enter the human body through various exposure pathways. These chemicals disrupt the normal functioning of the endocrine system, affecting hormone synthesis, secretion, metabolism, and receptor binding, thus increasing the risk of hormone-sensitive cancers like testicular and prostate cancer^39^. However, most existing evidence is derived from observational studies, which are inherently limited in their capacity to fully adjust for confounding factors such as genetic predisposition and lifestyle behaviors. Consequently, the causal role of EDCs in the development of testicular and prostate cancers remains uncertain and warrants further investigation through high-quality, well-controlled studies. In addition to the recognized risk factors for prostate cancer, such as genetic, environmental, and personal factors, complex screening and diagnostic strategies have played a crucial role in its management. PSA screening, the primary tool for early detection of prostate cancer^42^, has been widely utilized in several countries and regions, including the United States and Europe, since the 1990s. This approach has significantly improved the early diagnosis and treatment outcomes of localized prostate cancer, effectively reducing the incidence of advanced cases. As a result, the five-year survival rate for prostate cancer has significantly increased, approaching 100%^43–45^. However, the widespread use of PSA screening has contributed to overdiagnosis, particularly in the AYAMs population. The observed increase in prostate cancer incidence may not necessarily indicate a higher disease burden, but rather reflects the broader scope of screening efforts^46^.

Between 1990 and 2021, the incidence rates of testicular and prostate cancers among adolescents and young adult males (AYAM) exhibited a continuous upward trend. However, the overall age-standardized DALY rates declined, indicating a relative reduction in disease burden. This “rising incidence–declining DALY” pattern may reflect improvements in early detection, treatment efficacy, and patient quality of life. Notably, the relationship between DALY rates and sociodemographic development followed an “inverted U-shaped” pattern: DALY rates increased with SDI in regions below 0.7, but declined as SDI rose beyond this threshold. Although a significant correlation exists between SDI and cancer-related DALY rates, the ecological design of this study prevents causal inferences. Therefore, caution is required when interpreting these findings. The most significant age-standardized DALY rate increases occurred in middle and upper-middle SDI regions, likely reflecting disparities in healthcare accessibility, advancements in treatment, and regional differences in medical resources. In low-SDI regions, the cancer burden continued to rise, driven by limited healthcare infrastructure, low screening rates, poor health awareness, and delayed treatment. Studies suggest that financial constraints in low- and middle-income regions often hinder cancer care, leading to delayed diagnosis and treatment barriers^47,48^. Additionally, the absence of early diagnostic tools—such as PSA testing or ultrasound screening—frequently results in late-stage diagnoses of testicular and prostate cancer, exacerbating the disease burden. The rapid age-standardized DALY rate increase for testicular and prostate cancer in middle-SDI regions (e.g., parts of Latin America and Africa) underscores a growing cancer burden linked to urbanization, lifestyle shifts, environmental pollution, and disparities in medical resources. Despite healthcare improvements, inequities in medical resource distribution, insufficient cancer screening coverage, and limited treatment accessibility continue to hinder early diagnosis and timely intervention. In high-SDI regions, the decline in cancer-related DALY rates reflects the effectiveness of prevention and treatment strategies in reducing the disease burden. The most significant reduction in testicular cancer DALY rates was observed in the High-income Asia Pacific region, largely driven by treatment advancements. Countries such as Japan, South Korea, and Singapore have benefited from well-developed healthcare systems and the widespread use of cisplatin-based chemotherapy, leading to high cure rates^49^. Additionally, improved early diagnosis and accessible healthcare services have enabled timely treatment, further reducing mortality and disease burden. In Western Europe, prostate cancer DALY rates have also declined significantly, primarily due to widespread early screening and advancements in treatment^50^. The extensive use of PSA testing has improved early detection, allowing for timely intervention and reducing the risk of disease progression. Additionally, innovations in prostate cancer treatment—such as robotic-assisted surgery, advanced radiotherapy techniques, and precision-based hormonal and immunotherapies—have improved survival outcomes while minimizing treatment-related side effects, enhancing patients’ quality of life^51^. Despite these advancements, challenges remain. Over-screening may lead to overdiagnosis, particularly in prostate cancer, resulting in unnecessary treatment and increased healthcare costs. Future efforts should focus on optimizing screening strategies, advancing precision medicine, and strengthening long-term survivorship care to ensure continued reductions in cancer burden while improving patient outcomes and quality of life.

Our study also reveals that testicular cancer incidence peaks in the 25–29 age group before declining. While the overall DALY rate shows a downward trend, the long-term side effects of chemotherapy, such as infertility, nephrotoxicity, and neurotoxicity, remain a concern. Testicular cancer primarily affects young men, and with long life expectancy post-cure, it is essential to prioritize their quality of life. Prostate cancer incidence increases with age, highlighting the growing susceptibility as men age. Therefore, alongside enhancing current screening and treatment efforts, greater emphasis should be placed on personalized treatment to manage the expanding disease burden.

This study has several limitations. First, it is based on data from GBD 2021, which may be subject to publication bias. In low- and middle-income countries where cancer registry systems are incomplete, limited data availability and lower data quality may compromise the accuracy of estimates. Although the GBD employs imputation models, including extrapolation from neighboring or demographically similar regions, such methods may introduce bias and lead to discrepancies between estimated and actual values. Second, substantial cross-country variation in cancer screening practices may influence incidence estimates. In high-income countries, widespread screening may result in overestimation due to overdiagnosis, whereas in low-income settings, limited diagnostic capacity may lead to underestimation. Additionally, disability-adjusted life year (DALY) estimates are affected by factors such as disease stage and life expectancy, which may limit the comparability of results across regions. Third, this study focused exclusively on testicular and prostate cancers due to data constraints, excluding other male reproductive system malignancies (e.g., penile and epididymal cancers), thereby limiting the comprehensiveness of the overall burden assessment. Fourth, the EAPC assumes a linear trend over time, which may not adequately capture potential nonlinear dynamics in certain regions or periods. Moreover, as an ecological analysis, associations between the sociodemographic index (SDI) and cancer burden are derived from aggregated population-level data, which precludes individual-level causal inference and may introduce ecological fallacy. Finally, this study did not examine histological subtypes of tumors, limiting insight into cancer heterogeneity and its temporal evolution. Future research should incorporate higher-quality national registry data, individual-level information, and molecular subtyping to enhance the precision and interpretability of epidemiological trends.

## Conclusion

Male reproductive system cancers represent a significant global public health challenge among adolescent and young adult males (AYAMs), particularly testicular and prostate cancers, which have shown a continuous rise in incidence since 1990. Although the global burden of male reproductive system cancers, as measured by DALYs, has declined, substantial regional disparities persist. The cancer burden and mortality rates remain particularly high in low- and middle-income countries. As globalization accelerates, the influence of social factors on cancer risk has become increasingly pronounced. Healthcare providers must recognize and address these evolving health challenges to mitigate the growing cancer risk among AYAMs.

## Electronic supplementary material

Below is the link to the electronic supplementary material.

Supplementary Material 1

Supplementary Material 2

## Data Availability

The datasets analyzed during the current study are available in the the Global Burden of Disease (GBD) 2021 repository, https://vizhub.healthdata.org/gbd-results/.

## Abbreviations

AYAMs: Adolescents and young adult males
DALYs: Disability-adjusted life years
GBD: Global Burden of Disease
EAPC: Estimated annual percentage changes
SDI: Socio-demographic index
ASR: Age-standardized rate
EDCs: Endocrine-disrupting chemicals
PBDEs: Polybrominated diphenyl ethers
