# Supplementary material for: Global trends in testicular and prostate cancer among adolescents and young adult males aged 15–49 years, 1990–2021: insights from the GBD study
Source: Sci Rep. 2025 Jul 2;15:23388. doi: 10.1038/s41598-025-07361-3 (PMC12222491; doi:10.1038/s41598-025-07361-3)
Supplement: Supplementary file 1 — Supplementary Material 1 [file 41598_2025_7361_MOESM1_ESM.docx]

**Supplementary Appendix**

**Contents**

[Table S1. Age-standardized incidence rates and DALY rates in 1990 and 2021, and their estimated annual percentage changes from 1990 to 2021 for overall male cancers among adolescents and young adult males (15–49 years), by country. 2](#_Toc183632983)

[Table S2. Cases and age-standardized rates of incidence and DALYs in 1990 and 2021, and their estimated annual percentage changes from 1990 to 2021 for testicular cancer in adolescents and young adult males (15–49 years), globally and by 21 GBD regions. 11](#_Toc183632984)

[Table S3. Age-standardized rates of incidence and DALYs in 2021, and their estimated annual percentage changes from 1990 to 2021 for testicular cancer in adolescents and young adult males (15–49 years), by country. 14](#_Toc183632985)

[Table S4. Cases and age-standardized rates of incidence and DALYs in 1990 and 2021, and their estimated annual percentage changes from 1990 to 2021 for prostate cancer in adolescents and young adult males (15–49 years), globally and by 21 GBD regions. 23](#_Toc183632986)

[Table S5. Age-standardized rates of incidence and DALYs in 2021, and their estimated annual percentage changes from 1990 to 2021 for prostate cancer in adolescents and young adult males (15–49 years), by country. 26](#_Toc183632987)

[Figure S1. Age-standardized incidence and DALY rates in 2021, and their estimated annual percentage changes from 1990 to 2021 for testicular cancer, by country 35](#_Toc183632988)

[Figure S2. Age-standardized incidence and DALY rates in 2021, and their estimated annual percentage changes from 1990 to 2021 for prostate cancer, by country 36](#_Toc183632989)

[Figure S3. Age-standardized rates of incidence and DALYs in 2021, and their estimated annual percentage change (1990–2021) of overall male cancers, globally and for the 204 countries and territories, by SDI in 2021 37](#_Toc183632990)

[Figure S4. Age-standardized rates of incidence and DALYs in 2021, and their estimated annual percentage change (1990–2021) of testicular cancer, globally and for the 204 countries and territories, by SDI in 2021. 38](#_Toc183632991)

[Figure S5. Age-standardized rates of incidence and DALYs in 2021, and their estimated annual percentage change (1990–2021) of prostate cancer, globally and for the 204 countries and territories, by SDI in 2021.. 39](#_Toc183632992)

**Table S1. Age-standardized incidence rates and DALY rates in 1990 and 2021, and their estimated annual percentage changes from 1990 to 2021 for overall male cancers among adolescents and young adult males (15–49 years), by country.**

| Location name | Age-standardized incidence rate, 1990 | Age-standardized incidence rate, 2021 | EAPC of incidence rate | Age-standardized DALY rate, 1990 | Age-standardized DALY rate, 2021 | EAPC of DALY rate |
| --- | --- | --- | --- | --- | --- | --- |
| Afghanistan | 0.30 (0.14-0.58) | 0.80 (0.36-1.51) | 3.57 (3.24 to 3.9) | 7.25 (3.39-13.45) | 11.65 (5.45-21.75) | 1.89 (1.76 to 2.03) |
| Albania | 3.02 (1.83-4.74) | 8.45 (4.45-14.68) | 4.22 (3.72 to 4.73) | 50.98 (32.29-78.15) | 43.20 (24.28-70.56) | -0.18 (-0.36 to 0) |
| Algeria | 0.90 (0.48-1.56) | 2.19 (1.07-4.09) | 2.74 (2.51 to 2.97) | 6.88 (3.87-11.41) | 6.13 (3.16-10.93) | -0.43 (-0.65 to -0.22) |
| American Samoa | 1.67 (0.93-2.85) | 2.57 (1.43-4.41) | 1.86 (1.1 to 2.63) | 37.12 (20.63-62.91) | 45.49 (26.21-76.83) | 1.14 (0.42 to 1.88) |
| Andorra | 10.53 (5.10-19.18) | 15.00 (7.11-27.63) | 1.38 (1 to 1.76) | 37.88 (19.87-66.67) | 28.09 (13.81-50.73) | -0.64 (-0.78 to -0.5) |
| Angola | 0.74 (0.36-1.30) | 1.31 (0.66-2.37) | 2.07 (1.83 to 2.3) | 28.22 (13.83-50.13) | 34.72 (17.78-61.82) | 0.89 (0.73 to 1.04) |
| Antigua and Barbuda | 2.81 (2.09-3.69) | 4.93 (3.45-6.88) | 2.35 (2.03 to 2.68) | 32.86 (27.23-39.38) | 39.13 (30.43-50.12) | 1.11 (0.77 to 1.46) |
| Argentina | 6.07 (3.99-8.90) | 14.02 (9.40-20.03) | 2.91 (2.56 to 3.26) | 100.64 (70.02-138.45) | 101.96 (76.80-131.85) | 0.37 (0.16 to 0.57) |
| Armenia | 2.20 (1.64-2.95) | 4.30 (3.03-5.92) | 2.45 (1.9 to 3) | 40.45 (33.58-48.75) | 40.06 (32.01-49.17) | -0.03 (-0.55 to 0.49) |
| Australia | 14.14 (10.11-19.14) | 18.82 (12.44-26.99) | 0.83 (0.28 to 1.39) | 53.74 (42.25-67.09) | 32.63 (22.85-46.01) | -1.69 (-1.96 to -1.41) |
| Austria | 13.07 (8.93-18.63) | 13.26 (8.49-19.40) | 0.23 (-0.15 to 0.62) | 59.33 (45.10-76.28) | 27.10 (18.83-37.51) | -2.29 (-2.48 to -2.11) |
| Azerbaijan | 1.35 (0.85-2.04) | 2.10 (1.20-3.52) | 1.91 (1.52 to 2.29) | 33.87 (22.24-49.18) | 29.60 (17.79-47.55) | -0.26 (-0.45 to -0.08) |
| Bahamas | 3.40 (2.54-4.46) | 5.40 (3.69-7.62) | 1.49 (1.33 to 1.66) | 45.11 (37.52-54.38) | 54.64 (41.10-71.90) | 0.49 (0.4 to 0.58) |
| Bahrain | 1.80 (0.93-3.08) | 4.23 (2.10-7.68) | 3.15 (2.72 to 3.57) | 12.58 (7.08-20.69) | 11.04 (5.52-19.86) | -0.46 (-0.77 to -0.15) |
| Bangladesh | 0.76 (0.35-1.39) | 1.64 (0.77-3.06) | 2.44 (2.13 to 2.74) | 27.26 (12.54-49.85) | 24.81 (12.05-44.84) | -0.44 (-0.61 to -0.27) |
| Barbados | 2.59 (1.77-3.70) | 3.92 (2.38-6.08) | 1.39 (0.92 to 1.85) | 29.12 (21.64-38.74) | 30.08 (19.66-44.17) | 0.21 (-0.2 to 0.62) |
| Belarus | 3.28 (2.21-4.70) | 8.88 (5.57-13.33) | 3.08 (2.81 to 3.35) | 33.89 (24.83-45.30) | 42.12 (28.90-59.62) | 0.24 (-0.18 to 0.66) |
| Belgium | 8.63 (5.80-12.43) | 11.21 (7.24-16.63) | 0.8 (0.57 to 1.04) | 38.57 (28.77-50.47) | 23.66 (16.61-32.87) | -1.71 (-1.85 to -1.56) |
| Belize | 0.82 (0.63-1.06) | 3.51 (2.58-4.75) | 4.18 (3.5 to 4.86) | 12.99 (10.83-15.37) | 41.84 (33.88-51.41) | 3.14 (2.29 to 3.99) |
| Benin | 0.69 (0.39-1.15) | 0.94 (0.45-1.71) | 0.79 (0.67 to 0.91) | 24.18 (13.44-40.19) | 23.34 (11.46-41.88) | -0.33 (-0.47 to -0.19) |
| Bermuda | 2.86 (1.97-4.03) | 7.21 (4.48-10.89) | 2.97 (2.66 to 3.29) | 23.67 (17.55-30.74) | 24.48 (16.72-34.94) | 0.06 (-0.05 to 0.17) |
| Bhutan | 0.61 (0.26-1.21) | 1.31 (0.59-2.53) | 2.6 (2.37 to 2.83) | 21.94 (9.33-42.88) | 21.76 (9.96-41.53) | 0 (-0.1 to 0.11) |
| Bolivia (Plurinational State of) | 1.60 (0.86-2.76) | 4.18 (1.99-7.79) | 3.17 (3.01 to 3.33) | 54.93 (29.47-95.47) | 77.32 (37.72-140.83) | 1.07 (0.89 to 1.26) |
| Bosnia and Herzegovina | 3.18 (2.08-4.75) | 6.58 (3.74-10.35) | 2.99 (2.31 to 3.67) | 42.98 (29.95-60.51) | 34.21 (20.81-50.80) | -0.7 (-1.02 to -0.37) |
| Botswana | 1.43 (0.71-2.61) | 2.17 (1.05-3.91) | 1.16 (1.07 to 1.25) | 44.09 (21.89-80.03) | 45.90 (22.38-83.64) | -0.15 (-0.32 to 0.02) |
| Brazil | 2.05 (1.82-2.32) | 5.84 (5.04-6.75) | 3.34 (3.2 to 3.47) | 42.33 (38.26-46.82) | 58.24 (51.83-65.12) | 1.05 (0.98 to 1.11) |
| Brunei Darussalam | 1.97 (1.02-3.44) | 3.44 (1.85-5.83) | 1.93 (1.75 to 2.1) | 36.52 (19.78-62.51) | 31.16 (17.84-50.55) | -0.36 (-0.48 to -0.23) |
| Bulgaria | 9.57 (6.99-12.90) | 16.22 (10.66-24.06) | 2.09 (1.57 to 2.6) | 133.54 (105.68-164.38) | 115.66 (83.58-155.75) | -0.13 (-0.52 to 0.26) |
| Burkina Faso | 0.62 (0.32-1.09) | 0.84 (0.40-1.54) | 0.95 (0.82 to 1.09) | 21.34 (11.02-37.45) | 22.50 (10.75-40.64) | 0.07 (-0.13 to 0.27) |
| Burundi | 0.91 (0.40-1.66) | 1.05 (0.48-1.95) | 0.39 (0.21 to 0.57) | 34.45 (15.55-63.07) | 32.29 (14.87-60.51) | -0.3 (-0.46 to -0.15) |
| Cabo Verde | 0.66 (0.34-1.20) | 2.31 (1.18-4.11) | 4.35 (3.93 to 4.78) | 16.71 (9.10-29.99) | 31.62 (16.61-54.23) | 2 (1.57 to 2.43) |
| Cambodia | 0.54 (0.29-0.95) | 1.17 (0.57-2.14) | 2.67 (2.53 to 2.8) | 18.91 (10.13-33.19) | 23.80 (12.00-43.72) | 0.78 (0.75 to 0.8) |
| Cameroon | 0.83 (0.46-1.39) | 1.26 (0.57-2.29) | 1.2 (1.09 to 1.32) | 27.32 (15.21-45.36) | 29.66 (13.43-53.76) | 0.12 (0.03 to 0.21) |
| Canada | 11.33 (7.82-15.88) | 20.97 (13.96-29.13) | 2.11 (1.98 to 2.25) | 35.79 (27.94-45.06) | 36.31 (25.80-49.73) | -0.01 (-0.19 to 0.18) |
| Central African Republic | 0.72 (0.34-1.35) | 0.82 (0.37-1.59) | 0.34 (0.26 to 0.42) | 28.68 (13.32-54.06) | 29.71 (13.09-56.54) | 0.06 (-0.02 to 0.14) |
| Chad | 0.54 (0.28-0.95) | 0.81 (0.40-1.46) | 1.33 (1.22 to 1.43) | 19.76 (10.00-34.87) | 24.18 (11.94-43.42) | 0.62 (0.45 to 0.79) |
| Chile | 9.32 (6.65-12.75) | 33.55 (22.32-48.66) | 4.4 (3.97 to 4.83) | 134.55 (105.23-170.53) | 131.89 (97.70-172.05) | -0.02 (-0.29 to 0.26) |
| China | 0.65 (0.44-0.84) | 1.74 (1.24-2.37) | 3.18 (2.91 to 3.45) | 17.72 (12.10-22.88) | 12.36 (8.80-16.33) | -1.66 (-1.96 to -1.36) |
| Colombia | 3.32 (2.43-4.43) | 9.82 (6.45-14.37) | 3.87 (3.55 to 4.19) | 55.94 (44.24-69.51) | 59.70 (43.37-80.60) | 0.65 (0.41 to 0.88) |
| Comoros | 1.05 (0.39-2.00) | 1.70 (0.82-3.11) | 1.16 (0.74 to 1.58) | 36.39 (13.87-69.10) | 43.69 (21.35-79.97) | 0.09 (-0.39 to 0.57) |
| Congo | 0.99 (0.46-1.89) | 1.58 (0.75-2.86) | 1.55 (1.37 to 1.74) | 35.35 (16.68-66.79) | 38.26 (18.61-69.33) | 0.24 (0.12 to 0.36) |
| Cook Islands | 2.63 (1.44-4.37) | 5.80 (3.14-10.00) | 2.89 (2.75 to 3.02) | 48.04 (27.32-79.29) | 51.06 (28.89-85.64) | 0.49 (0.34 to 0.65) |
| Costa Rica | 4.08 (2.85-5.70) | 15.41 (9.83-22.79) | 4.27 (4.09 to 4.45) | 38.80 (29.56-49.99) | 75.14 (53.71-102.14) | 2.03 (1.81 to 2.25) |
| Croatia | 9.75 (6.69-13.89) | 14.57 (9.43-21.44) | 1.88 (1.47 to 2.28) | 69.03 (52.12-89.46) | 45.03 (32.32-61.31) | -1.04 (-1.38 to -0.69) |
| Cuba | 2.19 (1.51-3.04) | 6.70 (4.31-9.91) | 3.15 (2.64 to 3.67) | 20.37 (15.42-26.57) | 35.84 (25.16-49.49) | 1.41 (0.94 to 1.89) |
| Cyprus | 4.68 (2.42-8.29) | 13.11 (6.85-22.65) | 3.56 (2.98 to 4.15) | 31.76 (17.39-53.77) | 26.77 (14.63-45.27) | -0.87 (-1.1 to -0.64) |
| Czechia | 11.76 (8.40-16.11) | 20.98 (13.30-31.43) | 2.29 (2.02 to 2.57) | 98.93 (78.68-123.05) | 62.19 (44.48-85.47) | -0.9 (-1.24 to -0.56) |
| Côte d’Ivoire | 0.93 (0.52-1.55) | 1.53 (0.79-2.68) | 1.43 (1.3 to 1.57) | 30.12 (16.64-50.04) | 35.09 (18.38-60.51) | 0.3 (0.15 to 0.45) |
| Democratic People's Republic of Korea | 0.83 (0.41-1.54) | 1.19 (0.56-2.27) | 1.14 (0.91 to 1.36) | 17.23 (8.55-30.96) | 14.87 (7.42-27.69) | -0.64 (-0.75 to -0.52) |
| Democratic Republic of the Congo | 0.69 (0.35-1.20) | 1.04 (0.51-1.91) | 1.39 (1.1 to 1.69) | 25.09 (12.83-44.43) | 28.94 (14.29-53.29) | 0.58 (0.37 to 0.78) |
| Denmark | 13.58 (9.31-19.19) | 14.10 (9.29-20.09) | 0.39 (-0.09 to 0.87) | 57.07 (43.83-73.69) | 26.58 (18.65-37.34) | -2.54 (-2.8 to -2.29) |
| Djibouti | 1.27 (0.56-2.36) | 2.03 (0.93-3.84) | 1.44 (1.33 to 1.54) | 42.08 (18.95-78.59) | 48.95 (22.53-92.23) | 0.41 (0.28 to 0.54) |
| Dominica | 1.77 (0.99-2.89) | 3.20 (1.74-5.53) | 1.83 (1.65 to 2.01) | 27.05 (15.52-43.02) | 41.12 (23.52-69.30) | 1.46 (1.3 to 1.62) |
| Dominican Republic | 1.10 (0.67-1.71) | 1.89 (0.97-3.49) | 1.88 (1.55 to 2.2) | 20.94 (13.07-32.44) | 23.55 (12.44-41.71) | 0.69 (0.51 to 0.88) |
| Ecuador | 0.85 (0.62-1.14) | 5.46 (3.44-8.17) | 5.68 (4.1 to 7.28) | 20.30 (15.39-26.40) | 57.59 (39.39-81.01) | 3.04 (1.6 to 4.49) |
| Egypt | 0.62 (0.34-1.04) | 2.26 (1.16-3.94) | 3.94 (3.73 to 4.14) | 7.75 (4.53-12.55) | 10.41 (5.55-17.38) | 1.08 (0.95 to 1.21) |
| El Salvador | 2.06 (1.34-3.01) | 7.87 (4.52-12.56) | 4.61 (4.32 to 4.89) | 39.76 (27.37-55.28) | 58.66 (35.90-87.94) | 1.5 (1.4 to 1.61) |
| Equatorial Guinea | 0.79 (0.38-1.49) | 2.18 (1.03-4.12) | 3.8 (3.62 to 3.99) | 30.55 (14.73-56.94) | 38.65 (18.41-72.53) | 0.87 (0.81 to 0.94) |
| Eritrea | 0.98 (0.43-1.79) | 1.48 (0.68-2.75) | 1.22 (1.13 to 1.3) | 37.04 (16.63-68.26) | 44.86 (20.16-82.66) | 0.54 (0.45 to 0.62) |
| Estonia | 7.31 (4.98-10.35) | 9.94 (6.44-14.70) | 0.78 (0.43 to 1.13) | 68.08 (51.22-89.42) | 38.29 (27.21-52.76) | -2.62 (-2.87 to -2.37) |
| Eswatini | 1.16 (0.63-1.99) | 2.03 (0.92-3.85) | 1.81 (1.63 to 1.98) | 35.38 (18.78-60.49) | 49.23 (22.38-94.37) | 1.11 (0.81 to 1.4) |
| Ethiopia | 0.62 (0.27-1.10) | 1.17 (0.56-1.87) | 2.11 (1.85 to 2.38) | 25.73 (11.30-46.30) | 29.27 (13.92-46.30) | 0.41 (0.29 to 0.54) |
| Fiji | 1.85 (0.87-3.32) | 2.68 (1.23-4.92) | 1.35 (1.01 to 1.68) | 46.81 (22.54-82.85) | 54.70 (25.51-100.56) | 0.83 (0.51 to 1.15) |
| Finland | 6.11 (4.23-8.45) | 9.57 (6.22-13.95) | 2.11 (1.78 to 2.44) | 36.94 (28.08-47.22) | 21.53 (15.17-29.94) | -1.39 (-1.53 to -1.26) |
| France | 13.09 (9.33-17.88) | 22.58 (15.03-32.19) | 2.41 (2.1 to 2.73) | 58.42 (47.67-71.19) | 39.27 (27.53-54.48) | -0.91 (-1.09 to -0.74) |
| Gabon | 1.09 (0.53-1.99) | 2.06 (0.95-3.91) | 1.9 (1.76 to 2.04) | 35.66 (17.71-63.03) | 40.59 (19.14-75.64) | 0.25 (0.15 to 0.35) |
| Gambia | 0.68 (0.36-1.17) | 1.09 (0.53-2.02) | 1.13 (0.79 to 1.48) | 22.03 (11.70-37.02) | 25.85 (12.90-46.77) | 0.11 (-0.22 to 0.44) |
| Georgia | 6.85 (4.47-10.75) | 10.05 (7.26-13.74) | 0.88 (0.3 to 1.47) | 97.69 (71.49-140.16) | 108.05 (86.34-132.98) | 0.4 (-0.07 to 0.88) |
| Germany | 15.16 (11.24-20.02) | 17.85 (11.98-25.22) | 0.57 (0.18 to 0.97) | 78.70 (66.70-93.28) | 34.48 (25.25-46.11) | -2.29 (-2.47 to -2.1) |
| Ghana | 1.07 (0.56-1.91) | 1.48 (0.79-2.65) | 0.65 (0.42 to 0.88) | 33.86 (17.62-60.26) | 31.82 (17.14-54.83) | -0.49 (-0.68 to -0.31) |
| Greece | 14.91 (10.69-20.35) | 17.84 (12.78-24.54) | 0.72 (0.3 to 1.14) | 52.50 (43.12-63.67) | 39.60 (31.72-49.67) | -0.7 (-0.89 to -0.51) |
| Greenland | 6.67 (3.79-11.34) | 5.11 (2.51-9.04) | -0.32 (-0.93 to 0.29) | 104.80 (61.95-169.56) | 35.39 (17.96-60.36) | -3.21 (-3.79 to -2.62) |
| Grenada | 2.86 (2.00-4.01) | 6.05 (4.14-8.44) | 2.4 (1.87 to 2.94) | 47.43 (34.73-62.55) | 68.04 (50.95-88.84) | 1.39 (0.95 to 1.82) |
| Guam | 1.18 (0.74-1.82) | 2.87 (1.77-4.28) | 3.61 (3.15 to 4.07) | 17.45 (11.33-25.94) | 31.19 (20.52-44.18) | 2.41 (2.04 to 2.78) |
| Guatemala | 1.77 (1.41-2.20) | 5.09 (3.72-6.79) | 3.35 (2.74 to 3.97) | 47.53 (40.12-55.97) | 71.72 (57.58-87.70) | 1.45 (1.02 to 1.88) |
| Guinea | 0.94 (0.49-1.61) | 1.50 (0.77-2.65) | 1.37 (1.3 to 1.44) | 35.07 (18.35-60.79) | 44.57 (22.95-77.56) | 0.68 (0.63 to 0.74) |
| Guinea-Bissau | 0.73 (0.38-1.32) | 1.08 (0.51-2.01) | 1.26 (1.23 to 1.29) | 28.10 (14.62-50.88) | 32.23 (15.16-59.93) | 0.38 (0.31 to 0.44) |
| Guyana | 1.31 (0.90-1.82) | 3.81 (2.37-5.76) | 3.29 (2.83 to 3.75) | 29.51 (21.03-39.53) | 68.38 (44.17-100.91) | 2.77 (2.28 to 3.27) |
| Haiti | 0.99 (0.54-1.61) | 1.44 (0.75-2.49) | 1.38 (1.29 to 1.47) | 30.45 (16.70-49.75) | 37.48 (19.38-64.67) | 0.89 (0.8 to 0.99) |
| Honduras | 1.04 (0.58-1.75) | 1.71 (0.76-3.19) | 1.41 (1.25 to 1.57) | 24.84 (14.08-41.62) | 25.16 (11.54-47.10) | -0.1 (-0.19 to -0.01) |
| Hungary | 12.23 (8.79-16.82) | 21.52 (13.97-31.44) | 1.5 (1.2 to 1.81) | 127.52 (100.94-158.22) | 80.62 (58.98-107.50) | -1.63 (-1.88 to -1.38) |
| Iceland | 8.45 (5.70-12.14) | 10.05 (6.71-14.26) | 0.84 (0.51 to 1.16) | 29.30 (21.96-38.44) | 19.51 (13.60-27.27) | -1.15 (-1.32 to -0.98) |
| India | 0.88 (0.70-1.08) | 1.49 (1.21-1.82) | 1.78 (1.46 to 2.09) | 28.59 (22.45-35.48) | 23.42 (19.16-28.59) | -0.62 (-0.79 to -0.45) |
| Indonesia | 0.64 (0.45-0.88) | 1.34 (0.80-2.10) | 2.28 (2.2 to 2.37) | 18.72 (13.04-25.35) | 23.84 (14.38-37.18) | 0.79 (0.72 to 0.86) |
| Iran (Islamic Republic of) | 2.13 (1.10-3.52) | 10.13 (7.25-13.43) | 6.11 (5.56 to 6.66) | 12.79 (6.78-18.80) | 21.85 (14.14-29.06) | 2.66 (2.19 to 3.13) |
| Iraq | 1.48 (0.73-2.66) | 4.48 (2.18-8.19) | 4.07 (3.78 to 4.36) | 12.04 (6.33-20.67) | 13.76 (7.22-24.42) | 0.58 (0.51 to 0.64) |
| Ireland | 9.39 (6.23-13.64) | 15.79 (10.28-22.49) | 2.61 (2.16 to 3.06) | 45.01 (33.25-59.31) | 27.71 (19.05-38.58) | -1.08 (-1.3 to -0.86) |
| Israel | 3.68 (2.43-5.32) | 5.96 (3.87-8.74) | 1.81 (1.19 to 2.44) | 22.62 (16.51-30.22) | 14.18 (9.89-19.70) | -1.48 (-1.9 to -1.05) |
| Italy | 10.92 (8.07-14.44) | 20.51 (15.40-25.97) | 2.69 (2.28 to 3.1) | 42.45 (38.07-47.75) | 36.94 (30.96-44.65) | 0.17 (-0.1 to 0.44) |
| Jamaica | 1.15 (0.79-1.58) | 3.43 (2.02-5.40) | 2.86 (1.77 to 3.95) | 13.71 (10.28-17.97) | 31.12 (19.55-46.18) | 1.97 (0.94 to 3.01) |
| Japan | 6.94 (5.27-9.10) | 8.25 (6.26-10.36) | 0.31 (-0.23 to 0.85) | 26.06 (23.95-28.51) | 16.53 (14.52-19.01) | -1.71 (-2.06 to -1.36) |
| Jordan | 2.64 (1.36-4.76) | 10.22 (5.24-18.34) | 5.55 (5.05 to 6.05) | 16.15 (8.91-27.52) | 22.08 (11.91-37.95) | 1.59 (1.24 to 1.94) |
| Kazakhstan | 2.53 (1.80-3.50) | 4.55 (3.08-6.50) | 2.37 (2.02 to 2.72) | 50.25 (38.55-64.71) | 43.92 (33.44-56.23) | -0.47 (-0.99 to 0.05) |
| Kenya | 0.33 (0.18-0.51) | 0.61 (0.40-0.88) | 1.84 (1.76 to 1.93) | 9.08 (4.88-13.78) | 12.94 (8.52-18.34) | 1.21 (1.07 to 1.34) |
| Kiribati | 0.52 (0.26-0.90) | 0.70 (0.34-1.24) | 0.94 (0.92 to 0.95) | 18.63 (9.47-32.73) | 21.45 (10.53-38.32) | 0.45 (0.43 to 0.48) |
| Kuwait | 3.07 (1.97-4.57) | 6.52 (4.26-9.55) | 1.18 (-0.4 to 2.78) | 7.92 (5.46-11.07) | 12.75 (8.18-19.09) | 0.07 (-1.14 to 1.29) |
| Kyrgyzstan | 2.60 (1.71-3.77) | 3.11 (2.03-4.52) | 0.61 (-0.01 to 1.24) | 57.80 (38.92-81.73) | 39.51 (27.68-53.57) | -1.3 (-1.7 to -0.89) |
| Lao People's Democratic Republic | 0.48 (0.25-0.87) | 0.80 (0.40-1.44) | 1.71 (1.57 to 1.84) | 18.17 (9.44-33.43) | 19.31 (9.83-34.81) | 0.2 (0.18 to 0.23) |
| Latvia | 4.94 (3.38-6.97) | 10.83 (7.00-15.86) | 1.68 (1.22 to 2.14) | 65.14 (48.36-85.94) | 75.33 (53.86-102.24) | -0.39 (-0.74 to -0.04) |
| Lebanon | 2.66 (1.30-4.93) | 12.44 (6.48-21.19) | 5.63 (5.33 to 5.92) | 15.44 (8.04-27.39) | 23.62 (12.77-39.81) | 1.78 (1.61 to 1.95) |
| Lesotho | 0.90 (0.46-1.57) | 1.62 (0.79-3.02) | 1.94 (1.78 to 2.11) | 28.67 (14.52-50.02) | 46.33 (22.72-86.19) | 1.71 (1.51 to 1.91) |
| Liberia | 0.64 (0.34-1.11) | 1.04 (0.47-2.01) | 1.95 (1.76 to 2.13) | 23.12 (12.26-39.95) | 23.72 (10.51-45.90) | 0.38 (0.2 to 0.56) |
| Libya | 1.33 (0.65-2.40) | 2.87 (1.41-5.32) | 2.92 (2.57 to 3.28) | 9.80 (5.12-16.71) | 11.63 (6.10-20.72) | 0.73 (0.63 to 0.83) |
| Lithuania | 5.34 (3.67-7.50) | 12.43 (8.12-18.14) | 2.25 (1.92 to 2.57) | 41.38 (31.23-53.70) | 58.57 (41.90-78.09) | 0.83 (0.49 to 1.17) |
| Luxembourg | 12.39 (8.96-17.01) | 9.56 (6.59-13.17) | -0.67 (-1.08 to -0.26) | 64.06 (53.01-77.27) | 18.70 (14.22-24.19) | -3.85 (-4.04 to -3.67) |
| Madagascar | 0.86 (0.41-1.50) | 1.13 (0.55-2.09) | 0.71 (0.57 to 0.84) | 30.25 (14.54-52.97) | 31.76 (15.74-57.97) | 0.01 (-0.11 to 0.13) |
| Malawi | 1.19 (0.62-2.02) | 2.40 (1.25-4.24) | 2.35 (2.28 to 2.42) | 44.56 (22.92-75.59) | 65.34 (34.47-114.23) | 1.32 (1.29 to 1.36) |
| Malaysia | 1.23 (0.65-2.10) | 2.89 (1.51-5.01) | 2.73 (2.56 to 2.9) | 25.60 (13.91-42.97) | 26.65 (14.72-43.95) | -0.03 (-0.23 to 0.17) |
| Maldives | 0.35 (0.17-0.65) | 1.35 (0.69-2.50) | 5.25 (4.64 to 5.85) | 9.68 (4.71-17.52) | 10.29 (5.39-18.34) | 0.55 (0.19 to 0.9) |
| Mali | 0.78 (0.43-1.29) | 1.12 (0.57-2.02) | 1.23 (1.07 to 1.4) | 28.55 (15.96-46.73) | 30.71 (15.64-54.04) | 0.3 (0.13 to 0.47) |
| Malta | 8.89 (5.86-12.92) | 15.12 (9.68-22.69) | 2.19 (1.75 to 2.63) | 44.49 (32.37-59.50) | 31.10 (21.44-43.84) | -0.77 (-1.03 to -0.52) |
| Marshall Islands | 0.76 (0.42-1.28) | 1.17 (0.59-2.05) | 1.5 (1.43 to 1.58) | 23.66 (12.81-39.74) | 29.55 (15.00-50.23) | 0.83 (0.76 to 0.89) |
| Mauritania | 0.88 (0.45-1.56) | 1.57 (0.71-2.96) | 1.48 (1.34 to 1.61) | 30.18 (15.42-52.37) | 28.37 (13.05-53.25) | -0.52 (-0.62 to -0.42) |
| Mauritius | 1.20 (0.92-1.55) | 4.33 (3.20-5.72) | 3.7 (3.21 to 4.2) | 20.27 (17.03-24.04) | 41.36 (34.30-49.19) | 2.05 (1.5 to 2.6) |
| Mexico | 3.85 (3.56-4.15) | 14.27 (12.38-16.33) | 4.24 (4.08 to 4.41) | 77.76 (73.37-82.43) | 136.14 (120.35-153.16) | 2.04 (1.79 to 2.29) |
| Micronesia (Federated States of) | 0.83 (0.45-1.41) | 1.32 (0.67-2.32) | 1.63 (1.57 to 1.69) | 26.04 (13.77-43.94) | 30.35 (15.21-53.05) | 0.55 (0.52 to 0.58) |
| Monaco | 47.88 (23.54-86.37) | 79.35 (36.34-151.67) | 1.76 (1.59 to 1.93) | 144.09 (74.79-246.72) | 141.23 (67.08-260.42) | -0.01 (-0.05 to 0.03) |
| Mongolia | 0.74 (0.39-1.27) | 1.81 (1.00-3.01) | 3.5 (3.18 to 3.81) | 24.48 (13.12-41.95) | 30.19 (17.54-48.59) | 0.87 (0.76 to 0.99) |
| Montenegro | 10.85 (6.61-16.84) | 23.13 (13.54-37.22) | 2.98 (2.71 to 3.25) | 65.39 (43.68-93.99) | 85.66 (55.04-125.93) | 0.85 (0.58 to 1.13) |
| Morocco | 0.32 (0.17-0.56) | 0.68 (0.32-1.32) | 2.34 (2.22 to 2.46) | 3.88 (2.14-6.66) | 3.70 (1.90-6.90) | -0.13 (-0.21 to -0.04) |
| Mozambique | 0.60 (0.29-1.10) | 1.14 (0.54-2.16) | 2.29 (2.21 to 2.38) | 23.07 (11.11-41.27) | 35.41 (16.75-67.11) | 1.75 (1.64 to 1.86) |
| Myanmar | 0.45 (0.23-0.80) | 0.80 (0.40-1.45) | 1.85 (1.65 to 2.04) | 15.77 (8.12-27.53) | 16.39 (8.46-29.10) | 0.06 (0.01 to 0.11) |
| Namibia | 1.61 (0.81-2.87) | 2.99 (1.44-5.43) | 1.78 (1.65 to 1.9) | 50.68 (25.65-89.98) | 58.57 (28.69-104.97) | 0.2 (0.02 to 0.39) |
| Nauru | 0.94 (0.45-1.87) | 1.36 (0.61-2.87) | 1.2 (1.04 to 1.36) | 26.68 (12.96-53.39) | 30.50 (13.86-62.85) | 0.49 (0.45 to 0.54) |
| Nepal | 0.56 (0.24-1.06) | 1.01 (0.46-1.89) | 1.89 (1.66 to 2.12) | 20.71 (8.80-39.83) | 19.28 (8.97-35.27) | -0.22 (-0.42 to -0.02) |
| Netherlands | 9.71 (6.79-13.46) | 14.74 (9.77-21.27) | 1.85 (1.59 to 2.11) | 40.74 (31.36-51.88) | 27.78 (19.49-38.18) | -1.14 (-1.27 to -1.01) |
| New Zealand | 10.31 (7.17-14.57) | 12.11 (8.41-16.94) | 0.09 (-0.33 to 0.51) | 56.95 (44.09-72.49) | 35.34 (26.65-46.36) | -2.01 (-2.42 to -1.6) |
| Nicaragua | 1.67 (0.98-2.69) | 4.73 (2.53-8.13) | 3.79 (3.6 to 3.99) | 30.11 (18.55-47.22) | 42.62 (23.79-70.66) | 1.41 (1.22 to 1.59) |
| Niger | 0.51 (0.25-0.93) | 0.66 (0.27-1.31) | 0.83 (0.77 to 0.89) | 19.09 (9.39-34.90) | 18.87 (7.61-37.99) | -0.2 (-0.33 to -0.07) |
| Nigeria | 0.55 (0.29-0.84) | 1.01 (0.43-1.64) | 2.19 (2 to 2.37) | 17.67 (8.94-26.41) | 22.05 (9.67-34.54) | 0.86 (0.72 to 1) |
| Niue | 1.06 (0.56-1.82) | 1.77 (0.91-3.18) | 1.71 (1.65 to 1.77) | 23.91 (12.66-40.98) | 26.53 (14.25-46.01) | 0.35 (0.31 to 0.39) |
| North Macedonia | 6.85 (4.43-10.26) | 13.94 (8.42-21.89) | 3.04 (2.65 to 3.44) | 95.99 (66.02-136.95) | 74.58 (48.26-108.56) | -0.54 (-0.71 to -0.36) |
| Northern Mariana Islands | 1.19 (0.61-2.14) | 1.97 (1.13-3.33) | 1.49 (0.92 to 2.05) | 18.49 (9.94-33.13) | 22.76 (13.76-36.62) | 0.87 (0.45 to 1.3) |
| Norway | 12.56 (9.32-16.51) | 13.07 (10.85-15.39) | -0.39 (-1.4 to 0.63) | 40.22 (35.44-45.84) | 22.01 (18.29-26.49) | -2.37 (-3.07 to -1.65) |
| Oman | 0.53 (0.25-0.99) | 1.68 (0.83-3.04) | 4.08 (3.64 to 4.51) | 3.56 (1.76-6.48) | 3.75 (1.87-6.91) | 0.71 (0.45 to 0.97) |
| Pakistan | 1.55 (1.00-2.25) | 2.70 (1.61-4.22) | 1.65 (1.6 to 1.7) | 53.32 (34.75-77.58) | 64.75 (39.69-99.66) | 0.48 (0.36 to 0.6) |
| Palau | 1.55 (0.86-2.70) | 2.19 (1.18-3.75) | 1.04 (0.96 to 1.12) | 35.10 (19.56-61.40) | 35.32 (19.25-60.23) | 0.03 (0 to 0.07) |
| Palestine | 2.47 (1.26-4.52) | 5.64 (2.95-9.96) | 2.52 (2.16 to 2.88) | 19.98 (10.67-35.08) | 19.81 (11.35-33.49) | -0.07 (-0.24 to 0.1) |
| Panama | 2.14 (1.53-2.94) | 7.85 (5.20-11.32) | 4.05 (3.56 to 4.53) | 24.86 (20.12-30.71) | 46.47 (34.48-60.81) | 2.09 (1.65 to 2.54) |
| Papua New Guinea | 0.54 (0.23-1.04) | 0.78 (0.34-1.46) | 1.19 (1.07 to 1.32) | 15.94 (6.85-30.98) | 20.57 (8.72-38.69) | 0.87 (0.78 to 0.97) |
| Paraguay | 1.74 (1.02-2.82) | 5.00 (2.57-8.92) | 3.77 (3.53 to 4.01) | 34.82 (21.25-55.00) | 54.40 (29.28-93.26) | 1.85 (1.57 to 2.13) |
| Peru | 2.48 (1.45-4.04) | 6.73 (3.61-11.46) | 3.53 (3.28 to 3.78) | 63.57 (38.10-102.42) | 50.24 (28.43-81.74) | -0.77 (-0.95 to -0.6) |
| Philippines | 1.21 (0.89-1.60) | 1.57 (1.11-2.14) | 0.51 (0.31 to 0.71) | 28.17 (21.56-34.56) | 28.15 (20.96-36.15) | -0.1 (-0.18 to -0.02) |
| Poland | 7.01 (5.62-8.66) | 17.98 (13.39-23.64) | 3.29 (3.12 to 3.46) | 91.83 (85.83-98.38) | 80.37 (70.93-90.62) | -0.32 (-0.51 to -0.12) |
| Portugal | 6.40 (4.43-9.07) | 10.37 (6.65-15.43) | 1.66 (1.33 to 1.98) | 53.95 (41.18-70.21) | 26.78 (18.90-37.04) | -2.32 (-2.52 to -2.13) |
| Puerto Rico | 2.79 (1.93-3.91) | 14.98 (9.38-22.61) | 4.99 (4.48 to 5.49) | 25.49 (19.07-33.14) | 60.06 (41.44-83.83) | 2.01 (1.54 to 2.48) |
| Qatar | 1.06 (0.53-1.89) | 4.05 (2.08-7.25) | 5.71 (5.14 to 6.29) | 7.98 (4.11-13.76) | 9.27 (4.65-16.59) | 0.96 (0.69 to 1.23) |
| Republic of Korea | 0.88 (0.54-1.34) | 2.93 (1.56-5.00) | 3.88 (3.63 to 4.12) | 12.15 (7.67-17.89) | 6.95 (3.83-11.57) | -2.14 (-2.47 to -1.81) |
| Republic of Moldova | 3.46 (2.60-4.53) | 5.30 (3.79-7.17) | 1.51 (1.12 to 1.89) | 48.75 (40.44-57.93) | 36.19 (29.53-43.61) | -0.72 (-0.96 to -0.47) |
| Romania | 4.81 (3.52-6.45) | 10.74 (7.04-15.79) | 3.23 (2.93 to 3.52) | 73.13 (58.53-90.56) | 55.27 (40.37-75.04) | -0.6 (-0.77 to -0.43) |
| Russian Federation | 4.59 (4.26-4.96) | 9.83 (8.70-10.90) | 2.29 (2.04 to 2.54) | 47.72 (44.59-51.05) | 44.65 (39.67-49.50) | -0.87 (-1.28 to -0.46) |
| Rwanda | 1.03 (0.47-1.88) | 1.61 (0.78-2.97) | 1.25 (1.02 to 1.48) | 39.97 (18.16-73.41) | 40.35 (19.81-73.40) | -0.27 (-0.42 to -0.12) |
| Saint Kitts and Nevis | 2.30 (1.79-2.91) | 5.14 (3.10-7.99) | 1.77 (1.31 to 2.22) | 44.10 (36.94-52.01) | 55.07 (35.32-83.11) | -0.18 (-0.7 to 0.34) |
| Saint Lucia | 2.72 (2.08-3.53) | 7.59 (5.37-10.47) | 3.07 (2.58 to 3.55) | 43.80 (36.93-51.37) | 78.01 (60.40-99.13) | 1.64 (1.23 to 2.04) |
| Saint Vincent and the Grenadines | 2.40 (1.83-3.16) | 5.92 (4.35-7.93) | 2.65 (2.36 to 2.93) | 37.45 (30.97-44.98) | 71.22 (57.59-86.68) | 1.83 (1.52 to 2.15) |
| Samoa | 2.64 (1.33-4.74) | 4.19 (2.01-7.73) | 1.41 (1.35 to 1.48) | 62.89 (32.45-111.08) | 67.31 (32.91-119.56) | 0.29 (0.22 to 0.37) |
| San Marino | 8.54 (4.44-15.07) | 9.15 (4.08-17.67) | 0.87 (0.43 to 1.32) | 26.31 (14.48-43.54) | 17.42 (7.88-32.91) | -0.62 (-0.9 to -0.33) |
| Sao Tome and Principe | 0.65 (0.34-1.10) | 1.17 (0.58-2.12) | 1.71 (1.55 to 1.87) | 19.41 (10.04-33.13) | 20.88 (10.74-36.88) | -0.04 (-0.24 to 0.17) |
| Saudi Arabia | 0.60 (0.29-1.17) | 3.35 (1.51-6.70) | 6.23 (5.96 to 6.5) | 7.38 (3.67-13.74) | 10.31 (4.87-20.06) | 1.32 (1.16 to 1.48) |
| Senegal | 0.83 (0.45-1.43) | 1.36 (0.67-2.46) | 1.54 (1.4 to 1.68) | 27.91 (15.28-47.53) | 31.67 (15.73-56.30) | 0.46 (0.32 to 0.6) |
| Serbia | 7.12 (3.79-12.45) | 14.92 (7.99-25.34) | 2.81 (2.55 to 3.08) | 69.84 (39.83-116.70) | 54.06 (31.82-84.82) | -0.72 (-0.97 to -0.47) |
| Seychelles | 2.61 (1.44-4.52) | 4.61 (2.52-7.91) | 2.05 (1.8 to 2.31) | 54.40 (30.41-91.17) | 55.07 (31.67-91.55) | 0.25 (0.05 to 0.45) |
| Sierra Leone | 0.59 (0.31-1.02) | 0.91 (0.43-1.68) | 1.44 (1.22 to 1.67) | 20.29 (10.76-34.48) | 23.11 (10.77-41.56) | 0.49 (0.33 to 0.65) |
| Singapore | 2.12 (1.43-3.03) | 5.49 (3.45-8.25) | 2.58 (2.09 to 3.08) | 16.86 (12.44-22.34) | 11.82 (7.99-16.81) | -1.85 (-2.44 to -1.26) |
| Slovakia | 10.23 (6.03-16.39) | 21.56 (11.29-37.51) | 2.77 (2.63 to 2.9) | 85.64 (53.42-130.58) | 72.03 (40.85-118.49) | -0.31 (-0.41 to -0.21) |
| Slovenia | 9.68 (6.64-13.80) | 18.32 (11.57-27.45) | 2.29 (1.93 to 2.65) | 63.28 (48.23-82.31) | 41.26 (28.63-58.19) | -1.67 (-2.06 to -1.28) |
| Solomon Islands | 0.64 (0.31-1.16) | 1.05 (0.55-1.82) | 1.69 (1.64 to 1.75) | 20.46 (9.99-37.21) | 27.96 (14.70-50.35) | 1.11 (1.06 to 1.17) |
| Somalia | 0.80 (0.31-1.61) | 0.84 (0.33-1.76) | 0.1 (0.06 to 0.15) | 30.53 (11.66-62.14) | 29.77 (11.07-62.74) | -0.16 (-0.2 to -0.11) |
| South Africa | 1.62 (1.21-2.12) | 2.64 (1.97-3.84) | 1.79 (1.6 to 1.98) | 40.87 (30.56-53.47) | 44.00 (32.92-63.15) | 0.32 (0.19 to 0.44) |
| South Sudan | 0.84 (0.36-1.61) | 1.13 (0.51-2.14) | 0.95 (0.77 to 1.14) | 29.05 (12.63-55.19) | 31.44 (13.94-60.83) | 0.22 (0.04 to 0.4) |
| Spain | 6.62 (4.66-9.06) | 7.91 (5.11-11.69) | 0.48 (0.16 to 0.81) | 39.98 (31.48-50.12) | 17.90 (12.77-24.96) | -2.66 (-2.79 to -2.54) |
| Sri Lanka | 0.81 (0.44-1.41) | 2.03 (0.99-3.67) | 2.92 (2.8 to 3.03) | 16.11 (8.97-27.03) | 14.19 (7.30-24.61) | -0.72 (-0.89 to -0.54) |
| Sudan | 0.46 (0.23-0.83) | 1.90 (0.86-3.67) | 4.93 (4.78 to 5.09) | 7.87 (4.04-13.87) | 13.37 (6.14-24.96) | 2.09 (1.94 to 2.24) |
| Suriname | 1.28 (0.78-1.98) | 2.92 (1.55-5.03) | 2.68 (2.3 to 3.06) | 25.72 (16.21-38.89) | 44.90 (24.56-75.84) | 1.71 (1.29 to 2.13) |
| Sweden | 9.44 (6.65-13.02) | 8.93 (6.12-12.57) | 0.97 (0.29 to 1.66) | 33.20 (25.65-42.32) | 16.24 (11.47-22.47) | -1.23 (-1.69 to -0.76) |
| Switzerland | 11.87 (8.15-16.91) | 8.16 (5.19-12.38) | -1.52 (-1.97 to -1.06) | 54.04 (40.81-69.75) | 18.44 (12.65-26.00) | -3.69 (-3.84 to -3.55) |
| Syrian Arab Republic | 1.21 (0.60-2.11) | 3.32 (1.65-6.01) | 3.22 (3.01 to 3.42) | 12.04 (6.17-19.57) | 11.68 (5.87-19.37) | -0.25 (-0.37 to -0.13) |
| Taiwan (Province of China) | 2.29 (1.60-3.17) | 5.51 (3.60-8.13) | 2.61 (2.23 to 3.01) | 18.59 (14.22-23.86) | 19.44 (13.96-26.27) | -0.14 (-0.56 to 0.27) |
| Tajikistan | 0.41 (0.25-0.64) | 0.52 (0.23-0.99) | 0.48 (0.14 to 0.83) | 12.12 (7.44-18.68) | 12.56 (5.70-24.26) | -0.15 (-0.42 to 0.12) |
| Thailand | 1.31 (0.68-2.19) | 5.19 (2.54-8.94) | 4.44 (4.27 to 4.61) | 22.96 (12.36-37.48) | 34.99 (16.91-59.15) | 1.19 (1.02 to 1.35) |
| Timor-Leste | 0.44 (0.20-0.81) | 0.76 (0.36-1.39) | 1.88 (1.53 to 2.22) | 14.90 (7.01-28.01) | 17.46 (8.71-31.81) | 0.42 (0.07 to 0.76) |
| Togo | 0.79 (0.43-1.33) | 1.21 (0.56-2.18) | 1.14 (1.04 to 1.25) | 25.87 (14.32-43.52) | 28.63 (13.29-51.08) | 0.11 (0 to 0.22) |
| Tokelau | 0.85 (0.42-1.56) | 1.73 (0.90-3.07) | 2.14 (2.04 to 2.23) | 23.31 (11.25-42.81) | 28.26 (15.31-49.88) | 0.48 (0.4 to 0.55) |
| Tonga | 1.15 (0.66-1.90) | 1.91 (1.04-3.29) | 1.58 (1.51 to 1.64) | 26.08 (15.43-42.38) | 32.02 (17.95-53.26) | 0.7 (0.63 to 0.77) |
| Trinidad and Tobago | 1.83 (1.44-2.33) | 5.24 (3.36-7.74) | 3.33 (2.89 to 3.77) | 28.35 (24.17-33.13) | 53.35 (37.58-73.30) | 1.71 (1.18 to 2.23) |
| Tunisia | 1.45 (0.74-2.56) | 4.34 (2.10-7.97) | 3.45 (3.36 to 3.54) | 8.25 (4.44-14.01) | 10.05 (5.04-17.80) | 0.6 (0.57 to 0.63) |
| Turkey | 7.61 (3.83-13.65) | 26.86 (15.02-44.67) | 4.7 (4.33 to 5.08) | 62.86 (32.94-109.06) | 55.02 (31.90-88.24) | -0.2 (-0.48 to 0.08) |
| Turkmenistan | 1.48 (1.08-1.98) | 5.33 (3.17-8.59) | 3.01 (2.34 to 3.68) | 35.71 (27.68-45.44) | 78.94 (51.67-122.21) | 1.45 (0.77 to 2.13) |
| Tuvalu | 0.69 (0.37-1.16) | 1.22 (0.65-2.12) | 1.83 (1.77 to 1.88) | 21.90 (11.84-37.10) | 25.95 (14.03-43.72) | 0.59 (0.56 to 0.62) |
| Uganda | 1.36 (0.75-2.25) | 2.42 (1.27-4.22) | 1.35 (1.2 to 1.5) | 44.79 (25.15-74.13) | 58.55 (31.48-101.99) | 0.31 (0.11 to 0.51) |
| Ukraine | 3.98 (2.63-5.74) | 5.68 (3.39-8.82) | 0.99 (0.66 to 1.32) | 69.60 (48.45-95.92) | 71.68 (44.68-106.38) | -0.21 (-0.51 to 0.1) |
| United Arab Emirates | 2.91 (1.40-5.55) | 7.00 (3.37-12.53) | 2.92 (2.69 to 3.15) | 22.70 (11.61-41.79) | 21.36 (10.85-37.72) | 0.09 (-0.15 to 0.32) |
| United Kingdom | 15.78 (14.35-17.33) | 12.39 (11.24-13.62) | -0.38 (-0.63 to -0.13) | 55.54 (52.25-59.47) | 25.97 (23.31-29.33) | -2.17 (-2.38 to -1.96) |
| United Republic of Tanzania | 1.20 (0.54-2.22) | 1.73 (0.82-3.16) | 1.1 (0.99 to 1.21) | 39.86 (18.13-73.36) | 42.09 (20.65-75.60) | 0.2 (0.12 to 0.27) |
| United States of America | 11.79 (11.03-12.64) | 16.82 (15.49-18.27) | 1 (0.83 to 1.16) | 42.76 (40.03-45.99) | 39.76 (36.03-44.15) | -0.32 (-0.43 to -0.2) |
| United States Virgin Islands | 2.60 (1.37-4.40) | 4.78 (2.18-9.38) | 2.32 (2.16 to 2.47) | 29.85 (16.51-50.07) | 40.98 (19.56-79.78) | 1.66 (1.49 to 1.83) |
| Uruguay | 6.89 (4.54-10.16) | 18.78 (12.13-27.72) | 3.16 (2.91 to 3.4) | 87.91 (62.05-123.29) | 108.71 (76.89-147.50) | 0.6 (0.4 to 0.79) |
| Uzbekistan | 0.94 (0.63-1.39) | 2.25 (1.50-3.26) | 2.9 (2.32 to 3.49) | 20.66 (14.50-29.23) | 32.86 (23.54-45.00) | 1.41 (1.01 to 1.82) |
| Vanuatu | 0.63 (0.32-1.10) | 0.93 (0.50-1.56) | 1.2 (1.15 to 1.24) | 18.64 (9.58-32.65) | 24.44 (13.30-41.22) | 0.8 (0.75 to 0.85) |
| Venezuela (Bolivarian Republic of) | 1.90 (1.46-2.47) | 7.21 (4.67-10.69) | 4.26 (3.67 to 4.86) | 25.93 (21.94-30.35) | 59.33 (41.85-81.59) | 2.58 (2.12 to 3.05) |
| Viet Nam | 0.72 (0.37-1.26) | 1.95 (0.92-3.69) | 3.04 (2.87 to 3.22) | 16.08 (8.66-27.22) | 15.88 (7.97-28.63) | -0.26 (-0.39 to -0.13) |
| Yemen | 0.41 (0.17-0.78) | 1.16 (0.49-2.23) | 4.01 (3.68 to 4.35) | 6.93 (3.09-12.91) | 11.09 (4.80-21.00) | 1.85 (1.62 to 2.08) |
| Zambia | 0.99 (0.47-1.75) | 4.96 (1.94-9.78) | 6.63 (5.58 to 7.69) | 35.53 (17.21-63.02) | 125.55 (50.76-242.90) | 5.43 (4.53 to 6.33) |
| Zimbabwe | 1.12 (0.65-1.81) | 2.29 (1.13-4.03) | 2.13 (1.54 to 2.73) | 31.54 (18.09-51.10) | 62.08 (30.30-110.83) | 2.37 (1.83 to 2.91) |

**Table S2. Cases and age-standardized rates of incidence and DALYs in 1990 and 2021, and their estimated annual percentage changes from 1990 to 2021 for testicular cancer in adolescents and young adult males (15–49 years), globally and by 21 GBD regions.**

| **Characteristics** | **Incidence** | | | | | **DALYs** | | | | |
| --- | --- | --- | --- | --- | --- | --- | --- | --- | --- | --- |
|  | **Number of cases, 1990** | **Age-standardized rate per 100,000 population, 1990** | **Number of cases, 2021** | **Age-standardized rate per 100,000 population, 2021** | **Estimated annual percentage change, 1990–2021** | **Number of cases, 1990** | **Age-standardized rate per 100,000 population, 1990** | **Number of cases, 2021** | **Age-standardized rate per 100,000 population, 2021** | **Estimated annual percentage change, 1990–2021** |
| Andean Latin America | 122 (89-163) | 1.34 (0.85-2.08) | 851 (642-1107) | 4.79 (3.13-7.05) | 4.33 (3.76 to 4.9) | 3484 (2532-4610) | 37.89 (24.67-57.12) | 8032 (6146-10248) | 45.21 (31.51-62.84) | 0.58 (0.12 to 1.04) |
| Australasia | 674 (597-765) | 12.35 (9.20-16.34) | 1099 (932-1265) | 14.81 (10.21-20.56) | 0.34 (-0.07 to 0.75) | 2431 (2206-2646) | 44.62 (36.35-53.94) | 1937 (1637-2302) | 26.07 (19.23-35.45) | -1.94 (-2.15 to -1.74) |
| Caribbean | 28 (25-32) | 0.31 (0.25-0.40) | 239 (201-281) | 2.00 (1.51-2.61) | 5.29 (4.1 to 6.48) | 413 (369-476) | 4.59 (3.76-5.61) | 1662 (1402-1965) | 13.90 (10.94-17.59) | 3.06 (2.15 to 3.98) |
| Central Asia | 226 (189-279) | 1.39 (1.10-1.77) | 571 (484-687) | 2.26 (1.85-2.79) | 1.4 (0.98 to 1.83) | 3843 (3232-4624) | 23.57 (19.54-28.63) | 6249 (5311-7428) | 24.83 (20.87-29.77) | -0.16 (-0.37 to 0.04) |
| Central Europe | 2243 (2079-2464) | 7.12 (6.31-8.10) | 4136 (3714-4630) | 15.53 (13.21-18.24) | 2.88 (2.74 to 3.03) | 24669 (23182-26532) | 78.38 (72.41-85.47) | 16558 (14920-18375) | 61.44 (54.58-69.00) | -0.52 (-0.69 to -0.34) |
| Central Latin America | 857 (815-897) | 2.10 (1.94-2.28) | 5898 (5364-6460) | 9.06 (8.01-10.23) | 5.05 (4.88 to 5.23) | 19838 (19061-20628) | 48.21 (45.41-51.20) | 55885 (50879-60851) | 85.80 (77.03-94.83) | 2.21 (2.01 to 2.4) |
| Central Sub-Saharan Africa | 41 (29-56) | 0.37 (0.22-0.58) | 187 (128-266) | 0.60 (0.35-0.97) | 1.72 (1.42 to 2.01) | 1675 (1153-2242) | 14.44 (8.58-22.53) | 5361 (3682-7621) | 17.09 (9.78-28.04) | 0.71 (0.54 to 0.89) |
| East Asia | 1520 (1284-1776) | 0.44 (0.36-0.53) | 4512 (3628-5593) | 1.22 (0.93-1.62) | 3.29 (2.98 to 3.61) | 41841 (34836-49171) | 11.82 (9.52-14.50) | 27975 (22083-34709) | 7.71 (5.92-9.73) | -1.94 (-2.29 to -1.6) |
| Eastern Europe | 1861 (1712-2002) | 3.26 (2.93-3.67) | 3557 (3256-3850) | 6.92 (6.18-7.70) | 2.22 (1.97 to 2.46) | 21107 (19127-22933) | 37.13 (32.79-42.00) | 18412 (16252-20495) | 36.08 (31.22-41.74) | -0.75 (-1.09 to -0.41) |
| Eastern Sub-Saharan Africa | 190 (135-246) | 0.52 (0.35-0.70) | 982 (772-1213) | 1.02 (0.76-1.33) | 2.28 (2 to 2.55) | 7526 (5244-9971) | 19.80 (13.20-27.09) | 26595 (20661-33205) | 27.33 (20.27-35.66) | 1.13 (0.95 to 1.31) |
| High-income Asia Pacific | 2190 (1939-2487) | 4.79 (3.67-6.22) | 2383 (2139-2637) | 6.01 (4.68-7.38) | 0.43 (-0.08 to 0.94) | 8500 (7934-9100) | 18.55 (16.91-20.49) | 4294 (3816-4875) | 10.72 (9.22-12.65) | -2.07 (-2.38 to -1.76) |
| High-income North America | 7026 (6771-7303) | 9.06 (8.41-9.79) | 11491 (10945-12089) | 13.46 (12.29-14.78) | 1.31 (1.21 to 1.41) | 24608 (23328-26313) | 31.85 (29.82-34.31) | 25747 (23569-28360) | 30.15 (27.26-33.65) | -0.1 (-0.27 to 0.07) |
| North Africa and Middle East | 1586 (1231-2027) | 1.93 (1.26-3.00) | 10781 (9208-12631) | 6.16 (4.62-8.40) | 4.3 (4.01 to 4.59) | 11276 (8985-14123) | 13.89 (9.04-21.26) | 22976 (19713-27293) | 13.09 (10.10-17.06) | 0.15 (-0.13 to 0.43) |
| Oceania | 5 (4-7) | 0.33 (0.21-0.50) | 12 (10-16) | 0.35 (0.24-0.50) | 0.06 (-0.14 to 0.25) | 142 (98-194) | 8.66 (5.33-13.01) | 275 (211-355) | 7.61 (5.07-11.03) | -0.33 (-0.5 to -0.15) |
| South Asia | 2204 (1821-2592) | 0.84 (0.67-1.02) | 7538 (6495-8673) | 1.48 (1.22-1.77) | 1.89 (1.61 to 2.18) | 74483 (60712-87914) | 27.91 (22.21-34.39) | 130525 (111336-149867) | 25.53 (20.95-30.99) | -0.28 (-0.42 to -0.14) |
| Southeast Asia | 661 (576-765) | 0.58 (0.47-0.72) | 2556 (2066-3117) | 1.35 (1.04-1.75) | 2.6 (2.51 to 2.7) | 15772 (13837-18231) | 13.81 (11.47-16.73) | 30114 (24489-38256) | 15.96 (12.58-20.70) | 0.37 (0.31 to 0.43) |
| Southern Latin America | 773 (655-907) | 6.42 (4.86-8.38) | 3257 (2813-3780) | 18.72 (14.19-24.36) | 3.6 (3.25 to 3.96) | 11639 (10114-13276) | 96.51 (76.06-121.64) | 17627 (15475-19785) | 101.27 (81.40-123.37) | 0.36 (0.15 to 0.56) |
| Southern Sub-Saharan Africa | 80 (64-94) | 0.68 (0.53-0.85) | 231 (197-271) | 1.07 (0.85-1.32) | 1.63 (1.4 to 1.86) | 2034 (1644-2379) | 17.19 (13.30-21.29) | 4180 (3551-4903) | 19.39 (15.44-24.10) | 0.52 (0.34 to 0.7) |
| Tropical Latin America | 482 (449-518) | 1.25 (1.10-1.42) | 2603 (2356-2834) | 4.41 (3.78-5.13) | 4.32 (4.19 to 4.46) | 10493 (9869-11118) | 27.02 (24.30-30.04) | 25366 (23314-27200) | 42.99 (38.08-48.32) | 1.73 (1.58 to 1.89) |
| Western Europe | 10992 (10471-11621) | 11.08 (9.94-12.37) | 13160 (12194-14291) | 13.67 (11.85-15.82) | 1.01 (0.74 to 1.28) | 45841 (43253-48826) | 46.20 (42.76-50.34) | 23328 (20580-26738) | 24.08 (20.60-28.51) | -1.7 (-1.85 to -1.56) |
| Western Sub-Saharan Africa | 96 (78-115) | 0.24 (0.19-0.30) | 319 (244-396) | 0.31 (0.23-0.40) | 0.5 (0.3 to 0.7) | 3510 (2840-4210) | 8.55 (6.65-10.78) | 8267 (6465-10272) | 7.90 (5.97-10.25) | -0.52 (-0.68 to -0.35) |

**Table S3. Age-standardized rates of incidence and DALYs in 2021, and their estimated annual percentage changes from 1990 to 2021 for testicular cancer in adolescents and young adult males (15–49 years), by country.**

| Location name | Age-standardized incidence rate, 1990 | Age-standardized incidence rate, 2021 | EAPC of incidence rate | Age-standardized DALY rate, 1990 | Age-standardized DALY rate, 2021 | EAPC of DALY rate |
| --- | --- | --- | --- | --- | --- | --- |
| Afghanistan | 0.11 (0.05-0.22) | 0.45 (0.21-0.84) | 5.24 (4.85 to 5.63) | 2.47 (1.12-4.65) | 5.67 (2.75-10.25) | 3.44 (3.16 to 3.72) |
| Albania | 2.77 (1.69-4.29) | 7.87 (4.12-13.67) | 4.29 (3.78 to 4.8) | 45.16 (28.99-67.98) | 37.36 (20.84-60.89) | -0.24 (-0.43 to -0.06) |
| Algeria | 0.82 (0.43-1.44) | 2.04 (0.98-3.83) | 2.78 (2.54 to 3.02) | 5.91 (3.29-9.88) | 5.21 (2.64-9.40) | -0.45 (-0.69 to -0.2) |
| American Samoa | 0.03 (0.01-0.05) | 0.08 (0.04-0.15) | 4.67 (3.56 to 5.79) | 0.50 (0.27-0.86) | 1.21 (0.64-2.11) | 4.03 (2.89 to 5.18) |
| Andorra | 9.32 (4.43-17.11) | 13.01 (6.14-24.06) | 1.28 (0.9 to 1.65) | 29.41 (14.89-52.80) | 21.36 (10.48-39.25) | -0.75 (-0.87 to -0.63) |
| Angola | 0.39 (0.19-0.69) | 0.71 (0.34-1.31) | 2.29 (2 to 2.57) | 15.74 (7.70-28.54) | 19.67 (9.87-35.67) | 1.04 (0.84 to 1.23) |
| Antigua and Barbuda | 0.23 (0.17-0.30) | 1.95 (1.33-2.81) | 7.39 (5.93 to 8.88) | 3.37 (2.74-4.14) | 15.43 (11.54-20.25) | 5.21 (3.82 to 6.61) |
| Argentina | 5.42 (3.50-8.05) | 13.11 (8.76-18.75) | 3.04 (2.69 to 3.38) | 87.18 (59.43-121.56) | 92.53 (69.78-119.56) | 0.51 (0.3 to 0.72) |
| Armenia | 1.06 (0.69-1.56) | 2.50 (1.65-3.63) | 2.76 (2.3 to 3.23) | 15.22 (10.60-21.16) | 17.07 (12.18-22.89) | 0.07 (-0.34 to 0.48) |
| Australia | 13.13 (9.36-17.82) | 15.92 (10.49-22.84) | 0.4 (-0.04 to 0.84) | 43.95 (34.50-54.94) | 25.57 (17.71-36.42) | -1.88 (-2.09 to -1.67) |
| Austria | 10.89 (7.41-15.54) | 11.21 (7.10-16.51) | 0.32 (-0.07 to 0.71) | 48.84 (37.08-62.92) | 21.99 (15.15-30.73) | -2.28 (-2.5 to -2.06) |
| Azerbaijan | 0.55 (0.30-0.96) | 1.13 (0.59-2.02) | 3.08 (2.66 to 3.5) | 11.71 (6.43-19.93) | 12.26 (6.57-21.26) | 0.61 (0.44 to 0.79) |
| Bahamas | 0.04 (0.03-0.06) | 0.43 (0.29-0.61) | 7.7 (6.32 to 9.1) | 0.81 (0.65-0.99) | 4.75 (3.53-6.42) | 5.76 (4.63 to 6.89) |
| Bahrain | 1.21 (0.60-2.12) | 2.84 (1.43-5.05) | 3.37 (2.71 to 4.02) | 6.19 (3.29-10.46) | 4.93 (2.47-8.75) | -0.36 (-1.04 to 0.33) |
| Bangladesh | 0.67 (0.31-1.24) | 1.50 (0.71-2.75) | 2.55 (2.23 to 2.88) | 24.45 (11.17-45.05) | 22.41 (11.18-39.88) | -0.4 (-0.58 to -0.22) |
| Barbados | 0.18 (0.13-0.24) | 0.91 (0.59-1.35) | 4.66 (2.84 to 6.51) | 2.54 (2.09-3.03) | 6.94 (4.95-9.51) | 2.68 (1.04 to 4.35) |
| Belarus | 2.40 (1.59-3.51) | 5.97 (3.69-9.02) | 2.79 (2.58 to 3.01) | 23.59 (16.85-32.24) | 22.70 (15.36-32.34) | -0.66 (-1.1 to -0.22) |
| Belgium | 7.50 (4.99-10.87) | 9.60 (6.17-14.29) | 0.84 (0.62 to 1.07) | 29.32 (21.62-38.64) | 17.62 (12.18-24.89) | -1.6 (-1.79 to -1.41) |
| Belize | 0.10 (0.07-0.13) | 1.37 (0.99-1.86) | 8.33 (6.88 to 9.81) | 2.06 (1.68-2.51) | 17.76 (14.12-22.04) | 6.46 (4.88 to 8.06) |
| Benin | 0.41 (0.22-0.72) | 0.36 (0.18-0.65) | -1.06 (-1.29 to -0.83) | 15.14 (7.94-25.92) | 9.44 (4.80-16.95) | -2.05 (-2.29 to -1.81) |
| Bermuda | 0.19 (0.12-0.27) | 2.52 (1.54-3.91) | 8.46 (7.08 to 9.86) | 1.76 (1.26-2.38) | 6.99 (4.55-10.34) | 4.27 (3.3 to 5.24) |
| Bhutan | 0.54 (0.22-1.07) | 1.18 (0.54-2.25) | 2.69 (2.44 to 2.94) | 19.56 (8.29-38.55) | 19.50 (9.13-36.61) | 0.03 (-0.09 to 0.14) |
| Bolivia (Plurinational State of) | 1.06 (0.54-1.88) | 3.27 (1.52-6.18) | 3.79 (3.56 to 4.02) | 39.60 (20.23-70.74) | 62.24 (29.90-113.79) | 1.46 (1.21 to 1.7) |
| Bosnia and Herzegovina | 2.85 (1.87-4.28) | 6.00 (3.42-9.43) | 3.02 (2.31 to 3.73) | 36.70 (25.50-51.71) | 28.43 (17.44-42.28) | -0.82 (-1.18 to -0.46) |
| Botswana | 0.66 (0.31-1.25) | 1.02 (0.46-1.91) | 1.28 (1 to 1.55) | 20.99 (9.79-38.73) | 21.70 (10.05-40.08) | -0.08 (-0.21 to 0.04) |
| Brazil | 1.25 (1.10-1.43) | 4.43 (3.79-5.16) | 4.32 (4.18 to 4.47) | 27.05 (24.31-30.14) | 43.05 (37.99-48.45) | 1.72 (1.56 to 1.89) |
| Brunei Darussalam | 1.78 (0.91-3.11) | 3.09 (1.65-5.27) | 1.92 (1.73 to 2.11) | 30.34 (16.29-52.03) | 24.95 (14.14-40.60) | -0.45 (-0.59 to -0.32) |
| Bulgaria | 9.18 (6.67-12.40) | 15.47 (10.09-23.09) | 2.14 (1.59 to 2.7) | 125.54 (98.79-155.23) | 106.11 (75.71-144.49) | -0.09 (-0.54 to 0.35) |
| Burkina Faso | 0.36 (0.17-0.66) | 0.30 (0.15-0.55) | -0.98 (-1.22 to -0.73) | 12.97 (6.24-23.64) | 8.77 (4.41-15.71) | -1.68 (-1.97 to -1.4) |
| Burundi | 0.49 (0.24-0.89) | 0.63 (0.30-1.17) | 0.89 (0.67 to 1.1) | 20.02 (10.08-36.54) | 20.82 (9.84-38.67) | 0.16 (-0.02 to 0.34) |
| Cabo Verde | 0.08 (0.04-0.15) | 0.18 (0.08-0.37) | 2.83 (2.65 to 3.01) | 2.01 (1.05-3.55) | 2.14 (0.99-4.05) | 0.13 (0.08 to 0.17) |
| Cambodia | 0.33 (0.17-0.60) | 0.76 (0.36-1.42) | 2.85 (2.69 to 3.01) | 12.01 (6.13-21.62) | 15.33 (7.57-28.49) | 0.77 (0.74 to 0.8) |
| Cameroon | 0.49 (0.25-0.87) | 0.46 (0.22-0.86) | -0.72 (-0.95 to -0.49) | 16.86 (8.67-29.40) | 11.38 (5.44-20.68) | -1.7 (-1.89 to -1.51) |
| Canada | 9.70 (6.67-13.60) | 19.20 (12.75-26.61) | 2.49 (2.32 to 2.66) | 27.28 (21.16-34.55) | 30.35 (21.31-41.93) | 0.55 (0.27 to 0.84) |
| Central African Republic | 0.34 (0.16-0.63) | 0.38 (0.17-0.73) | 0.27 (0.16 to 0.39) | 14.77 (7.05-27.23) | 15.22 (6.98-28.96) | 0.07 (-0.03 to 0.18) |
| Chad | 0.33 (0.16-0.60) | 0.31 (0.15-0.55) | -0.48 (-0.66 to -0.3) | 12.74 (6.22-23.21) | 10.23 (5.17-18.55) | -0.98 (-1.22 to -0.73) |
| Chile | 8.61 (6.14-11.80) | 32.27 (21.46-46.83) | 4.51 (4.07 to 4.96) | 120.65 (94.57-152.47) | 123.07 (91.20-160.64) | 0.1 (-0.2 to 0.39) |
| China | 0.41 (0.33-0.51) | 1.19 (0.88-1.59) | 3.39 (3.05 to 3.73) | 11.82 (9.46-14.56) | 7.64 (5.78-9.74) | -1.99 (-2.33 to -1.64) |
| Colombia | 1.92 (1.43-2.52) | 7.69 (5.09-11.17) | 5.38 (5 to 5.76) | 41.23 (33.03-50.83) | 50.23 (36.75-67.28) | 1.37 (1.05 to 1.69) |
| Comoros | 0.61 (0.26-1.14) | 1.11 (0.54-2.00) | 1.44 (0.9 to 1.98) | 22.35 (9.42-41.62) | 29.53 (14.75-53.76) | 0.33 (-0.26 to 0.93) |
| Congo | 0.49 (0.25-0.87) | 0.88 (0.41-1.63) | 2.04 (1.85 to 2.23) | 18.60 (9.33-32.52) | 21.78 (10.67-40.28) | 0.63 (0.5 to 0.75) |
| Cook Islands | 0.27 (0.12-0.52) | 0.79 (0.36-1.52) | 3.62 (3.53 to 3.71) | 3.90 (1.82-7.19) | 4.46 (2.15-8.33) | 0.68 (0.54 to 0.82) |
| Costa Rica | 2.74 (1.91-3.85) | 11.26 (7.20-16.77) | 4.72 (4.48 to 4.95) | 30.72 (23.29-39.63) | 59.48 (42.29-80.96) | 2.12 (1.85 to 2.39) |
| Croatia | 8.94 (6.11-12.78) | 13.39 (8.62-19.75) | 1.95 (1.53 to 2.37) | 59.44 (44.67-77.20) | 37.49 (26.78-51.29) | -1.04 (-1.4 to -0.67) |
| Cuba | 0.47 (0.32-0.68) | 4.18 (2.64-6.28) | 6.26 (5.01 to 7.52) | 5.03 (3.65-6.74) | 21.37 (14.91-29.86) | 3.93 (2.82 to 5.06) |
| Cyprus | 4.17 (2.13-7.44) | 11.52 (6.00-19.86) | 3.47 (2.87 to 4.08) | 26.41 (14.22-45.20) | 20.90 (11.28-35.72) | -1.17 (-1.43 to -0.91) |
| Czechia | 11.03 (7.85-15.15) | 19.79 (12.49-29.76) | 2.32 (2.04 to 2.6) | 89.77 (71.27-111.78) | 55.20 (39.33-76.27) | -0.93 (-1.31 to -0.55) |
| Côte d’Ivoire | 0.30 (0.15-0.53) | 0.48 (0.23-0.86) | 1.24 (0.99 to 1.5) | 10.40 (5.28-18.36) | 11.55 (5.78-20.18) | 0.09 (-0.13 to 0.32) |
| Democratic People's Republic of Korea | 0.57 (0.27-1.07) | 0.75 (0.34-1.47) | 0.77 (0.48 to 1.06) | 11.26 (5.39-20.65) | 8.35 (3.96-16.02) | -1.23 (-1.4 to -1.07) |
| Democratic Republic of the Congo | 0.35 (0.17-0.63) | 0.54 (0.25-1.03) | 1.46 (1.11 to 1.81) | 13.61 (6.73-24.13) | 15.89 (7.26-30.28) | 0.65 (0.42 to 0.88) |
| Denmark | 12.78 (8.73-18.11) | 12.90 (8.47-18.36) | 0.3 (-0.18 to 0.78) | 46.68 (35.63-60.62) | 20.59 (14.11-29.42) | -2.64 (-2.9 to -2.38) |
| Djibouti | 0.80 (0.38-1.44) | 1.36 (0.63-2.57) | 1.7 (1.55 to 1.84) | 27.39 (13.29-49.90) | 33.70 (15.86-63.22) | 0.65 (0.5 to 0.79) |
| Dominica | 0.09 (0.05-0.14) | 0.59 (0.30-1.04) | 6.32 (5.63 to 7.03) | 1.93 (1.17-2.93) | 8.89 (4.73-15.29) | 5.26 (4.7 to 5.81) |
| Dominican Republic | 0.10 (0.06-0.16) | 0.19 (0.10-0.34) | 2.25 (1.42 to 3.07) | 2.68 (1.60-4.15) | 2.85 (1.47-4.85) | 0.49 (-0.07 to 1.05) |
| Ecuador | 0.32 (0.23-0.43) | 4.39 (2.78-6.53) | 8.12 (5.79 to 10.49) | 8.63 (6.43-11.29) | 45.63 (31.51-63.83) | 4.96 (2.9 to 7.06) |
| Egypt | 0.43 (0.23-0.74) | 1.73 (0.92-3.00) | 4.2 (3.97 to 4.44) | 4.57 (2.63-7.50) | 6.33 (3.55-10.36) | 1.23 (1.07 to 1.39) |
| El Salvador | 0.89 (0.59-1.28) | 4.27 (2.52-6.74) | 5.67 (5.32 to 6.01) | 24.48 (16.92-34.25) | 38.82 (24.68-58.18) | 1.88 (1.76 to 2.01) |
| Equatorial Guinea | 0.39 (0.19-0.73) | 1.27 (0.60-2.45) | 4.56 (4.34 to 4.78) | 16.10 (7.92-29.74) | 21.86 (10.50-41.53) | 1.21 (1.11 to 1.31) |
| Eritrea | 0.52 (0.27-0.94) | 0.89 (0.41-1.65) | 1.63 (1.54 to 1.71) | 21.36 (10.89-38.14) | 28.63 (13.26-52.26) | 0.92 (0.84 to 1) |
| Estonia | 5.28 (3.52-7.56) | 5.92 (3.78-8.81) | 0.32 (-0.16 to 0.79) | 47.58 (35.26-63.26) | 18.32 (12.63-25.47) | -3.67 (-3.97 to -3.37) |
| Eswatini | 0.45 (0.23-0.81) | 0.73 (0.34-1.36) | 1.33 (1.22 to 1.45) | 14.27 (7.27-24.76) | 18.53 (8.71-33.56) | 0.74 (0.57 to 0.91) |
| Ethiopia | 0.49 (0.21-0.90) | 1.00 (0.48-1.59) | 2.37 (2.1 to 2.65) | 21.32 (8.93-39.11) | 25.37 (12.08-39.74) | 0.59 (0.47 to 0.72) |
| Fiji | 1.31 (0.67-2.30) | 1.98 (1.00-3.52) | 1.41 (1 to 1.83) | 32.73 (17.26-56.53) | 39.65 (20.54-69.49) | 0.9 (0.51 to 1.29) |
| Finland | 4.84 (3.31-6.74) | 7.42 (4.78-10.93) | 2.16 (1.87 to 2.45) | 25.95 (19.60-33.49) | 14.84 (10.28-20.88) | -1.2 (-1.42 to -0.99) |
| France | 11.62 (8.28-15.82) | 19.98 (13.27-28.49) | 2.49 (2.17 to 2.8) | 47.86 (39.23-57.85) | 32.33 (22.56-45.01) | -0.75 (-0.97 to -0.54) |
| Gabon | 0.56 (0.27-1.02) | 1.13 (0.53-2.16) | 2.07 (1.9 to 2.24) | 18.87 (9.33-33.99) | 22.02 (10.73-41.39) | 0.31 (0.19 to 0.43) |
| Gambia | 0.59 (0.31-1.02) | 0.93 (0.44-1.73) | 1.01 (0.63 to 1.39) | 19.34 (10.11-32.71) | 22.12 (10.81-40.29) | 0.01 (-0.35 to 0.36) |
| Georgia | 5.35 (3.26-8.88) | 7.73 (5.46-10.75) | 0.61 (-0.09 to 1.32) | 66.90 (44.66-104.61) | 72.11 (56.06-91.04) | 0.09 (-0.5 to 0.69) |
| Germany | 14.09 (10.46-18.60) | 16.27 (10.88-23.01) | 0.52 (0.14 to 0.91) | 69.93 (59.49-82.29) | 28.92 (21.01-38.83) | -2.34 (-2.56 to -2.11) |
| Ghana | 0.45 (0.22-0.85) | 0.52 (0.24-1.01) | -0.17 (-0.43 to 0.1) | 14.88 (7.43-27.90) | 11.40 (5.43-21.58) | -1.28 (-1.47 to -1.09) |
| Greece | 13.88 (9.93-18.99) | 16.57 (11.86-22.83) | 0.73 (0.31 to 1.16) | 45.04 (36.73-54.91) | 34.27 (27.30-43.27) | -0.6 (-0.81 to -0.4) |
| Greenland | 6.22 (3.52-10.64) | 4.65 (2.26-8.28) | -0.39 (-1.04 to 0.27) | 96.56 (56.84-157.13) | 31.10 (15.43-53.45) | -3.36 (-3.99 to -2.73) |
| Grenada | 0.25 (0.19-0.32) | 2.85 (2.03-3.86) | 7.71 (6.58 to 8.86) | 5.78 (4.70-7.01) | 34.29 (27.05-42.77) | 5.82 (4.87 to 6.78) |
| Guam | 0.27 (0.16-0.41) | 0.84 (0.51-1.31) | 5.08 (4.47 to 5.7) | 2.80 (1.85-4.02) | 6.96 (4.63-10.07) | 4.15 (3.53 to 4.77) |
| Guatemala | 0.89 (0.73-1.10) | 3.38 (2.49-4.48) | 4.8 (4.22 to 5.39) | 32.10 (26.87-38.09) | 56.79 (45.74-69.43) | 2.28 (1.84 to 2.72) |
| Guinea | 0.73 (0.37-1.28) | 1.13 (0.56-2.04) | 1.2 (1.09 to 1.31) | 28.13 (14.39-49.89) | 34.78 (17.37-61.66) | 0.55 (0.48 to 0.63) |
| Guinea-Bissau | 0.39 (0.21-0.70) | 0.38 (0.19-0.69) | -0.55 (-0.72 to -0.39) | 16.51 (8.61-29.42) | 12.54 (6.26-22.93) | -1.26 (-1.44 to -1.09) |
| Guyana | 0.11 (0.08-0.16) | 1.17 (0.73-1.75) | 7.31 (5.88 to 8.76) | 3.63 (2.54-5.04) | 25.28 (16.37-36.99) | 6.2 (4.83 to 7.59) |
| Haiti | 0.14 (0.07-0.25) | 0.31 (0.15-0.58) | 3.03 (2.79 to 3.28) | 5.96 (2.87-10.87) | 10.61 (4.96-19.71) | 2.3 (2.07 to 2.53) |
| Honduras | 0.49 (0.26-0.84) | 0.82 (0.34-1.60) | 1.54 (1.41 to 1.67) | 16.16 (8.83-27.63) | 16.42 (7.14-31.67) | -0.06 (-0.14 to 0.03) |
| Hungary | 11.41 (8.18-15.70) | 20.44 (13.24-29.90) | 1.62 (1.32 to 1.91) | 113.74 (90.40-140.60) | 71.94 (52.55-96.05) | -1.51 (-1.81 to -1.22) |
| Iceland | 7.09 (4.73-10.22) | 8.05 (5.41-11.35) | 0.89 (0.52 to 1.26) | 20.24 (14.90-26.90) | 12.10 (8.17-17.35) | -1.11 (-1.38 to -0.84) |
| India | 0.80 (0.64-0.98) | 1.34 (1.10-1.61) | 1.77 (1.44 to 2.11) | 26.10 (20.89-32.24) | 20.93 (17.24-25.12) | -0.67 (-0.85 to -0.48) |
| Indonesia | 0.43 (0.31-0.59) | 0.92 (0.56-1.45) | 2.35 (2.22 to 2.47) | 12.65 (9.22-17.10) | 15.97 (9.84-25.11) | 0.75 (0.69 to 0.81) |
| Iran (Islamic Republic of) | 1.58 (0.87-2.68) | 8.81 (6.69-11.41) | 6.84 (6.19 to 7.49) | 5.53 (3.60-8.51) | 14.05 (10.87-18.17) | 4.6 (3.8 to 5.41) |
| Iraq | 1.26 (0.61-2.27) | 3.96 (1.90-7.30) | 4.22 (3.91 to 4.52) | 9.12 (4.67-15.70) | 10.62 (5.48-19.05) | 0.66 (0.59 to 0.72) |
| Ireland | 8.49 (5.60-12.42) | 14.34 (9.30-20.42) | 2.66 (2.22 to 3.11) | 37.16 (27.31-49.16) | 22.91 (15.56-32.13) | -1 (-1.23 to -0.77) |
| Israel | 3.27 (2.15-4.74) | 5.31 (3.43-7.83) | 1.86 (1.27 to 2.46) | 18.25 (13.20-24.49) | 11.39 (7.84-16.03) | -1.41 (-1.81 to -1.01) |
| Italy | 9.71 (7.13-12.85) | 19.28 (14.50-24.32) | 2.94 (2.52 to 3.37) | 34.70 (31.01-39.18) | 32.90 (27.49-39.89) | 0.58 (0.26 to 0.9) |
| Jamaica | 0.13 (0.08-0.18) | 0.98 (0.57-1.57) | 5.37 (3.77 to 6.99) | 1.92 (1.39-2.58) | 9.43 (5.92-13.95) | 3.85 (2.4 to 5.32) |
| Japan | 6.78 (5.13-8.91) | 7.85 (5.95-9.85) | 0.2 (-0.34 to 0.74) | 23.33 (21.34-25.64) | 13.77 (11.93-16.04) | -1.94 (-2.31 to -1.58) |
| Jordan | 2.30 (1.17-4.20) | 9.35 (4.78-16.82) | 5.74 (5.22 to 6.26) | 12.50 (6.77-21.77) | 17.89 (9.51-31.11) | 1.81 (1.41 to 2.22) |
| Kazakhstan | 1.68 (1.09-2.47) | 3.43 (2.21-5.07) | 2.73 (2.32 to 3.13) | 29.24 (19.87-41.24) | 27.97 (19.42-38.33) | -0.36 (-0.67 to -0.05) |
| Kenya | 0.14 (0.08-0.20) | 0.23 (0.16-0.32) | 1.52 (1.29 to 1.76) | 3.61 (2.05-5.19) | 5.00 (3.60-6.76) | 1.15 (1.07 to 1.24) |
| Kiribati | 0.14 (0.07-0.24) | 0.20 (0.10-0.36) | 1.1 (1 to 1.19) | 5.47 (2.68-9.66) | 6.70 (3.27-12.02) | 0.59 (0.52 to 0.67) |
| Kuwait | 2.74 (1.75-4.11) | 4.78 (3.16-6.94) | 0.66 (-1.45 to 2.81) | 6.04 (4.03-8.60) | 6.97 (4.33-10.75) | -0.74 (-2.86 to 1.43) |
| Kyrgyzstan | 1.94 (1.24-2.88) | 2.13 (1.37-3.16) | 0.26 (-0.52 to 1.04) | 40.05 (25.95-58.35) | 22.79 (15.83-31.16) | -1.98 (-2.53 to -1.42) |
| Lao People's Democratic Republic | 0.29 (0.14-0.54) | 0.51 (0.25-0.93) | 1.93 (1.73 to 2.13) | 11.47 (5.75-21.79) | 12.44 (6.23-22.80) | 0.27 (0.22 to 0.33) |
| Latvia | 3.60 (2.45-5.13) | 6.75 (4.37-10.03) | 1.01 (0.53 to 1.49) | 50.09 (36.62-67.07) | 46.00 (32.84-63.40) | -1.31 (-1.68 to -0.93) |
| Lebanon | 2.09 (1.01-3.85) | 10.89 (5.62-18.61) | 5.95 (5.62 to 6.28) | 9.78 (5.03-17.17) | 17.66 (9.37-30.26) | 2.31 (2.11 to 2.51) |
| Lesotho | 0.36 (0.18-0.65) | 0.59 (0.28-1.06) | 1.66 (1.55 to 1.77) | 12.10 (5.89-21.36) | 18.31 (8.80-33.85) | 1.62 (1.52 to 1.73) |
| Liberia | 0.40 (0.21-0.71) | 0.41 (0.19-0.78) | 0.34 (0.16 to 0.52) | 15.07 (7.77-26.39) | 9.66 (4.44-18.09) | -1.24 (-1.42 to -1.06) |
| Libya | 0.84 (0.40-1.56) | 1.85 (0.89-3.48) | 2.95 (2.58 to 3.33) | 4.54 (2.30-7.98) | 5.27 (2.67-9.65) | 0.71 (0.6 to 0.83) |
| Lithuania | 3.25 (2.19-4.63) | 6.04 (3.86-8.95) | 1.31 (0.92 to 1.7) | 26.12 (19.44-34.54) | 26.71 (18.70-36.33) | -0.4 (-0.78 to -0.01) |
| Luxembourg | 11.33 (8.16-15.63) | 8.56 (5.89-11.81) | -0.71 (-1.11 to -0.31) | 54.14 (44.58-65.49) | 15.11 (11.37-19.68) | -3.92 (-4.12 to -3.72) |
| Madagascar | 0.49 (0.26-0.87) | 0.74 (0.36-1.34) | 1.08 (0.92 to 1.25) | 18.47 (9.77-32.40) | 21.71 (10.99-38.87) | 0.38 (0.24 to 0.51) |
| Malawi | 0.93 (0.47-1.60) | 1.89 (0.96-3.38) | 2.42 (2.3 to 2.53) | 35.87 (17.94-61.93) | 52.70 (27.07-93.82) | 1.37 (1.32 to 1.42) |
| Malaysia | 1.06 (0.56-1.81) | 2.51 (1.30-4.37) | 2.71 (2.5 to 2.92) | 21.72 (11.71-36.31) | 22.06 (12.07-36.48) | -0.17 (-0.41 to 0.08) |
| Maldives | 0.22 (0.10-0.41) | 1.02 (0.52-1.81) | 6.06 (5.37 to 6.75) | 5.97 (2.88-10.97) | 6.79 (3.61-11.49) | 0.88 (0.47 to 1.29) |
| Mali | 0.63 (0.35-1.06) | 0.89 (0.44-1.63) | 1.13 (0.95 to 1.32) | 23.78 (13.03-39.39) | 25.03 (12.49-44.83) | 0.22 (0.03 to 0.4) |
| Malta | 8.41 (5.52-12.25) | 14.12 (8.99-21.23) | 2.18 (1.73 to 2.64) | 40.31 (29.19-54.10) | 27.29 (18.66-38.66) | -0.81 (-1.09 to -0.53) |
| Marshall Islands | 0.15 (0.07-0.26) | 0.21 (0.10-0.37) | 1.07 (0.96 to 1.17) | 4.86 (2.49-8.59) | 5.41 (2.71-9.47) | 0.37 (0.34 to 0.4) |
| Mauritania | 0.58 (0.28-1.07) | 0.73 (0.34-1.36) | 0.06 (-0.16 to 0.28) | 20.41 (9.78-36.87) | 12.96 (6.14-23.73) | -2.02 (-2.2 to -1.83) |
| Mauritius | 0.79 (0.59-1.03) | 3.10 (2.27-4.14) | 4.54 (3.68 to 5.41) | 12.21 (10.20-14.63) | 26.57 (21.86-31.85) | 2.94 (1.97 to 3.92) |
| Mexico | 2.85 (2.64-3.08) | 12.56 (10.97-14.30) | 5.02 (4.84 to 5.2) | 66.64 (62.85-70.66) | 125.90 (111.75-141.00) | 2.4 (2.11 to 2.68) |
| Micronesia (Federated States of) | 0.17 (0.08-0.29) | 0.25 (0.12-0.47) | 1.39 (1.35 to 1.44) | 5.60 (2.80-9.82) | 5.80 (2.97-10.33) | 0.09 (0.07 to 0.12) |
| Monaco | 46.62 (22.85-84.29) | 76.74 (34.93-147.15) | 1.74 (1.57 to 1.9) | 135.72 (70.03-233.52) | 131.75 (61.92-244.53) | -0.03 (-0.08 to 0.01) |
| Mongolia | 0.58 (0.31-0.99) | 1.40 (0.76-2.35) | 3.47 (3.1 to 3.85) | 18.94 (10.31-32.17) | 21.64 (12.43-34.74) | 0.59 (0.43 to 0.76) |
| Montenegro | 9.88 (6.00-15.38) | 21.61 (12.63-34.83) | 3.08 (2.81 to 3.35) | 54.13 (35.96-78.54) | 73.65 (47.30-107.58) | 0.99 (0.69 to 1.29) |
| Morocco | 0.22 (0.11-0.40) | 0.47 (0.21-0.94) | 2.3 (2.16 to 2.43) | 2.31 (1.22-4.07) | 2.03 (0.98-3.91) | -0.33 (-0.45 to -0.2) |
| Mozambique | 0.50 (0.24-0.92) | 0.94 (0.44-1.78) | 2.25 (2.15 to 2.35) | 19.61 (9.31-34.91) | 29.93 (14.02-56.85) | 1.73 (1.61 to 1.85) |
| Myanmar | 0.27 (0.14-0.50) | 0.51 (0.24-0.94) | 2.04 (1.75 to 2.32) | 9.98 (5.05-17.78) | 10.28 (5.12-18.60) | 0.04 (-0.05 to 0.13) |
| Namibia | 1.02 (0.51-1.82) | 1.87 (0.85-3.54) | 1.7 (1.55 to 1.85) | 32.93 (16.35-58.61) | 36.41 (17.32-67.34) | 0.06 (-0.09 to 0.2) |
| Nauru | 0.19 (0.09-0.33) | 0.24 (0.12-0.45) | 0.7 (0.35 to 1.06) | 5.41 (2.77-9.80) | 5.30 (2.71-9.37) | -0.07 (-0.1 to -0.04) |
| Nepal | 0.49 (0.20-0.94) | 0.89 (0.41-1.65) | 1.88 (1.64 to 2.12) | 18.54 (7.67-36.05) | 17.01 (8.02-30.78) | -0.27 (-0.48 to -0.07) |
| Netherlands | 8.87 (6.18-12.32) | 13.59 (8.97-19.65) | 1.9 (1.66 to 2.15) | 33.83 (25.90-43.18) | 23.37 (16.10-32.44) | -1.05 (-1.18 to -0.92) |
| New Zealand | 8.44 (5.85-11.94) | 9.60 (6.63-13.45) | -0.1 (-0.6 to 0.4) | 47.90 (36.96-61.17) | 28.60 (21.41-37.59) | -2.19 (-2.66 to -1.71) |
| Nicaragua | 0.85 (0.50-1.38) | 3.09 (1.62-5.42) | 4.84 (4.62 to 5.07) | 20.69 (12.70-32.84) | 32.54 (17.83-54.83) | 1.87 (1.67 to 2.07) |
| Niger | 0.31 (0.14-0.58) | 0.25 (0.11-0.48) | -1.11 (-1.3 to -0.93) | 12.13 (5.63-22.64) | 7.73 (3.37-15.21) | -1.97 (-2.22 to -1.73) |
| Nigeria | 0.04 (0.02-0.05) | 0.08 (0.05-0.11) | 2.55 (2.26 to 2.85) | 1.32 (0.85-1.87) | 1.70 (1.14-2.37) | 1.05 (0.86 to 1.25) |
| Niue | 0.25 (0.12-0.47) | 0.42 (0.20-0.81) | 1.61 (1.56 to 1.65) | 5.17 (2.58-9.31) | 5.30 (2.59-9.92) | 0.03 (0 to 0.06) |
| North Macedonia | 6.53 (4.21-9.80) | 13.42 (8.11-21.09) | 3.09 (2.69 to 3.49) | 89.61 (61.48-127.71) | 69.19 (44.97-100.37) | -0.54 (-0.7 to -0.37) |
| Northern Mariana Islands | 0.25 (0.12-0.48) | 0.44 (0.23-0.78) | 1.74 (0.64 to 2.84) | 2.98 (1.44-5.54) | 3.87 (2.14-6.46) | 1.31 (0.34 to 2.29) |
| Norway | 11.42 (8.43-15.07) | 11.66 (9.81-13.52) | -0.49 (-1.56 to 0.58) | 31.03 (27.13-35.77) | 17.47 (14.45-21.14) | -2.32 (-3.13 to -1.49) |
| Oman | 0.39 (0.18-0.73) | 1.36 (0.68-2.43) | 4.36 (3.83 to 4.89) | 2.08 (1.00-3.81) | 2.39 (1.17-4.31) | 1.07 (0.66 to 1.48) |
| Pakistan | 1.41 (0.91-2.04) | 2.45 (1.46-3.84) | 1.65 (1.59 to 1.7) | 48.86 (31.90-70.85) | 59.17 (36.18-91.30) | 0.47 (0.35 to 0.6) |
| Palau | 0.00 (0.00-0.00) | 0.00 (0.00-0.00) | 1.15 (1.04 to 1.26) | 0.01 (0.00-0.01) | 0.01 (0.00-0.01) | 0.07 (0.05 to 0.09) |
| Palestine | 1.73 (0.85-3.23) | 4.21 (2.18-7.55) | 2.74 (2.26 to 3.22) | 10.73 (5.50-19.44) | 11.04 (6.12-18.79) | 0.13 (-0.22 to 0.48) |
| Panama | 0.95 (0.72-1.26) | 5.02 (3.48-7.04) | 5.24 (4.7 to 5.77) | 15.96 (13.27-19.34) | 34.77 (26.55-44.36) | 2.61 (2.15 to 3.06) |
| Papua New Guinea | 0.11 (0.04-0.23) | 0.15 (0.07-0.29) | 0.84 (0.63 to 1.05) | 3.43 (1.21-7.00) | 4.25 (1.99-7.81) | 0.62 (0.48 to 0.76) |
| Paraguay | 1.23 (0.73-1.97) | 3.84 (1.93-6.99) | 4.17 (3.93 to 4.42) | 25.79 (15.77-40.25) | 41.62 (21.92-71.88) | 2.08 (1.8 to 2.37) |
| Peru | 1.90 (1.10-3.15) | 5.47 (2.90-9.30) | 3.78 (3.49 to 4.07) | 50.99 (30.26-83.20) | 39.46 (22.06-63.81) | -0.82 (-1.03 to -0.61) |
| Philippines | 0.74 (0.57-0.97) | 0.94 (0.68-1.26) | 0.4 (0.11 to 0.69) | 16.71 (13.72-20.13) | 16.25 (12.79-20.39) | -0.15 (-0.28 to -0.02) |
| Poland | 6.50 (5.18-8.06) | 17.09 (12.71-22.52) | 3.36 (3.18 to 3.55) | 80.09 (74.79-85.89) | 70.75 (62.46-79.83) | -0.26 (-0.51 to 0) |
| Portugal | 5.06 (3.50-7.19) | 8.26 (5.24-12.29) | 1.7 (1.41 to 2) | 42.66 (32.49-55.61) | 20.09 (14.00-28.19) | -2.49 (-2.74 to -2.23) |
| Puerto Rico | 0.72 (0.49-1.03) | 11.85 (7.40-17.93) | 8.37 (7.22 to 9.54) | 7.28 (5.34-9.67) | 44.92 (30.96-62.72) | 4.77 (3.75 to 5.79) |
| Qatar | 0.32 (0.15-0.58) | 2.24 (1.19-3.84) | 9.25 (7.98 to 10.54) | 1.20 (0.62-2.06) | 3.31 (1.60-5.90) | 5.67 (4.58 to 6.77) |
| Republic of Korea | 0.75 (0.46-1.14) | 2.60 (1.37-4.45) | 3.97 (3.73 to 4.21) | 8.72 (5.77-12.59) | 4.74 (2.53-8.00) | -2.36 (-2.84 to -1.88) |
| Republic of Moldova | 2.85 (2.12-3.76) | 4.13 (2.92-5.64) | 1.19 (0.74 to 1.65) | 39.53 (32.55-47.32) | 25.56 (20.73-31.11) | -1.3 (-1.59 to -1.01) |
| Romania | 4.45 (3.25-5.97) | 9.73 (6.34-14.37) | 3.25 (2.94 to 3.57) | 65.96 (52.88-81.43) | 46.43 (33.60-63.54) | -0.69 (-0.9 to -0.47) |
| Russian Federation | 3.45 (3.20-3.72) | 7.82 (6.96-8.66) | 2.44 (2.15 to 2.73) | 35.57 (33.30-38.00) | 32.82 (29.25-36.31) | -0.99 (-1.45 to -0.53) |
| Rwanda | 0.55 (0.28-1.01) | 1.02 (0.50-1.87) | 2.07 (1.79 to 2.36) | 23.11 (11.46-42.55) | 26.27 (13.15-46.98) | 0.36 (0.21 to 0.51) |
| Saint Kitts and Nevis | 0.21 (0.17-0.27) | 2.26 (1.26-3.76) | 6.54 (5.24 to 7.86) | 5.75 (4.67-6.96) | 25.85 (15.40-41.99) | 3.59 (2.44 to 4.75) |
| Saint Lucia | 0.30 (0.23-0.39) | 3.58 (2.49-4.95) | 7.67 (6.24 to 9.11) | 6.63 (5.45-8.00) | 38.50 (29.66-48.80) | 5.29 (4.08 to 6.51) |
| Saint Vincent and the Grenadines | 0.17 (0.13-0.22) | 1.77 (1.29-2.38) | 6.86 (5.66 to 8.08) | 3.64 (2.94-4.45) | 24.02 (19.29-29.50) | 5.34 (4.18 to 6.52) |
| Samoa | 2.31 (1.15-4.15) | 3.71 (1.76-6.89) | 1.44 (1.37 to 1.51) | 54.92 (28.24-97.29) | 59.06 (28.67-104.96) | 0.31 (0.24 to 0.38) |
| San Marino | 7.51 (3.83-13.37) | 8.05 (3.61-15.59) | 0.84 (0.42 to 1.26) | 19.89 (10.68-33.46) | 13.54 (6.12-25.58) | -0.55 (-0.82 to -0.29) |
| Sao Tome and Principe | 0.39 (0.18-0.71) | 0.59 (0.26-1.15) | 0.9 (0.65 to 1.14) | 11.93 (5.56-21.42) | 10.32 (4.77-19.57) | -0.91 (-1.19 to -0.63) |
| Saudi Arabia | 0.45 (0.22-0.82) | 2.75 (1.26-5.27) | 6.61 (6.25 to 6.96) | 5.67 (2.93-9.76) | 7.85 (3.81-14.22) | 1.31 (1.09 to 1.53) |
| Senegal | 0.52 (0.27-0.93) | 0.57 (0.28-1.03) | -0.13 (-0.31 to 0.06) | 18.25 (9.50-32.27) | 13.76 (7.00-24.96) | -1.12 (-1.28 to -0.96) |
| Serbia | 6.67 (3.52-11.72) | 14.16 (7.56-24.12) | 2.88 (2.61 to 3.16) | 62.08 (35.06-104.42) | 47.71 (28.02-75.05) | -0.69 (-0.95 to -0.43) |
| Seychelles | 1.25 (0.66-2.17) | 1.99 (1.01-3.55) | 1.92 (1.69 to 2.16) | 24.16 (13.06-40.49) | 19.80 (10.53-33.66) | -0.11 (-0.34 to 0.11) |
| Sierra Leone | 0.34 (0.17-0.62) | 0.35 (0.17-0.63) | -0.33 (-0.63 to -0.03) | 12.46 (6.14-22.02) | 9.43 (4.55-16.84) | -1.15 (-1.35 to -0.95) |
| Singapore | 1.99 (1.33-2.85) | 5.25 (3.29-7.89) | 2.63 (2.1 to 3.17) | 14.21 (10.41-18.90) | 10.01 (6.67-14.37) | -1.82 (-2.51 to -1.12) |
| Slovakia | 9.52 (5.59-15.29) | 20.56 (10.73-35.87) | 2.86 (2.71 to 3) | 75.17 (46.65-114.58) | 63.86 (35.99-105.35) | -0.25 (-0.37 to -0.12) |
| Slovenia | 8.41 (5.73-12.07) | 16.36 (10.31-24.54) | 2.46 (2.06 to 2.87) | 52.01 (39.45-68.14) | 33.68 (23.14-47.81) | -1.62 (-2.07 to -1.17) |
| Solomon Islands | 0.12 (0.04-0.24) | 0.19 (0.09-0.36) | 1.48 (1.32 to 1.65) | 4.19 (1.57-8.62) | 5.37 (2.49-9.83) | 0.87 (0.77 to 0.97) |
| Somalia | 0.42 (0.17-0.85) | 0.48 (0.17-1.02) | 0.41 (0.34 to 0.49) | 17.56 (6.72-35.63) | 18.50 (6.41-39.37) | 0.2 (0.14 to 0.26) |
| South Africa | 0.75 (0.56-0.95) | 1.13 (0.88-1.42) | 1.57 (1.27 to 1.87) | 18.07 (13.55-22.92) | 17.84 (14.13-22.22) | 0.08 (-0.09 to 0.25) |
| South Sudan | 0.47 (0.22-0.90) | 0.67 (0.29-1.31) | 1.19 (0.97 to 1.41) | 17.16 (7.92-32.47) | 19.54 (8.38-38.82) | 0.46 (0.25 to 0.67) |
| Spain | 5.66 (3.96-7.72) | 6.70 (4.30-9.94) | 0.46 (0.15 to 0.78) | 30.83 (24.26-38.75) | 13.49 (9.46-19.12) | -2.67 (-2.83 to -2.51) |
| Sri Lanka | 0.60 (0.31-1.05) | 1.67 (0.81-3.04) | 3.36 (3.23 to 3.49) | 11.30 (6.14-19.25) | 10.62 (5.46-18.57) | -0.44 (-0.59 to -0.29) |
| Sudan | 0.23 (0.11-0.42) | 1.32 (0.61-2.52) | 6.21 (6.03 to 6.39) | 3.22 (1.60-5.76) | 7.43 (3.52-13.48) | 3.38 (3.11 to 3.64) |
| Suriname | 0.27 (0.16-0.44) | 1.30 (0.67-2.30) | 4.83 (3.96 to 5.71) | 7.36 (4.24-11.35) | 22.62 (12.06-38.33) | 3.3 (2.47 to 4.14) |
| Sweden | 8.40 (5.88-11.69) | 7.77 (5.33-10.94) | 1 (0.3 to 1.7) | 25.66 (19.50-33.19) | 12.47 (8.69-17.43) | -1 (-1.53 to -0.46) |
| Switzerland | 10.31 (7.04-14.73) | 6.74 (4.24-10.34) | -1.75 (-2.22 to -1.28) | 44.26 (33.20-57.34) | 13.65 (9.22-19.49) | -4.1 (-4.28 to -3.93) |
| Syrian Arab Republic | 0.65 (0.31-1.20) | 2.10 (1.00-4.02) | 3.88 (3.58 to 4.17) | 4.60 (2.39-8.19) | 4.83 (2.31-8.91) | 0.25 (0.15 to 0.35) |
| Taiwan (Province of China) | 1.75 (1.22-2.44) | 4.02 (2.60-5.99) | 2.57 (2.19 to 2.94) | 12.32 (9.39-15.87) | 10.86 (7.53-14.94) | -0.51 (-0.92 to -0.1) |
| Tajikistan | 0.03 (0.02-0.06) | 0.05 (0.03-0.09) | 1.19 (0.76 to 1.62) | 0.84 (0.46-1.44) | 0.92 (0.48-1.53) | 0.4 (0.13 to 0.67) |
| Thailand | 0.90 (0.50-1.47) | 3.90 (2.16-6.50) | 4.74 (4.56 to 4.92) | 14.70 (8.79-23.21) | 22.59 (13.26-36.01) | 1.2 (1.03 to 1.37) |
| Timor-Leste | 0.29 (0.14-0.55) | 0.50 (0.24-0.93) | 1.83 (1.41 to 2.25) | 10.35 (4.94-19.60) | 11.65 (5.88-21.38) | 0.26 (-0.15 to 0.68) |
| Togo | 0.49 (0.25-0.87) | 0.46 (0.22-0.86) | -0.86 (-1.13 to -0.59) | 16.63 (8.70-29.35) | 11.50 (5.41-20.82) | -1.77 (-2 to -1.53) |
| Tokelau | 0.20 (0.10-0.38) | 0.47 (0.23-0.86) | 2.34 (2.13 to 2.55) | 5.44 (2.56-10.26) | 6.56 (3.35-11.77) | 0.27 (0.11 to 0.42) |
| Tonga | 0.20 (0.10-0.35) | 0.36 (0.17-0.68) | 1.8 (1.72 to 1.88) | 3.99 (2.14-7.05) | 5.16 (2.58-9.41) | 0.85 (0.74 to 0.96) |
| Trinidad and Tobago | 0.18 (0.14-0.23) | 1.87 (1.22-2.79) | 7.83 (6.35 to 9.32) | 3.78 (3.13-4.49) | 20.09 (14.25-27.40) | 5.16 (3.71 to 6.62) |
| Tunisia | 1.23 (0.62-2.19) | 3.78 (1.81-6.99) | 3.51 (3.41 to 3.61) | 6.03 (3.16-10.36) | 7.41 (3.63-13.33) | 0.66 (0.6 to 0.71) |
| Turkey | 6.92 (3.47-12.45) | 25.21 (14.10-41.86) | 4.83 (4.44 to 5.22) | 53.17 (27.60-92.66) | 47.16 (27.34-75.36) | -0.11 (-0.41 to 0.19) |
| Turkmenistan | 1.13 (0.78-1.57) | 4.49 (2.54-7.49) | 2.94 (2.12 to 3.76) | 25.72 (18.66-34.44) | 61.16 (37.50-99.72) | 1.2 (0.35 to 2.07) |
| Tuvalu | 0.13 (0.07-0.24) | 0.24 (0.11-0.43) | 1.69 (1.62 to 1.76) | 4.52 (2.31-8.20) | 4.89 (2.40-8.88) | 0.21 (0.19 to 0.22) |
| Uganda | 0.58 (0.31-1.00) | 1.13 (0.57-2.02) | 1.69 (1.51 to 1.86) | 20.43 (10.87-34.55) | 28.75 (14.96-50.55) | 0.6 (0.41 to 0.79) |
| Ukraine | 2.84 (1.75-4.29) | 4.51 (2.71-7.03) | 1.25 (0.98 to 1.52) | 44.39 (28.72-64.34) | 51.18 (32.32-74.59) | -0.01 (-0.23 to 0.2) |
| United Arab Emirates | 2.27 (1.07-4.41) | 6.08 (2.93-10.79) | 3.2 (2.93 to 3.47) | 14.51 (7.20-27.11) | 15.43 (7.98-26.75) | 0.42 (0.15 to 0.69) |
| United Kingdom | 14.74 (13.39-16.21) | 10.46 (9.48-11.52) | -0.7 (-0.97 to -0.44) | 47.62 (44.68-51.19) | 18.80 (16.66-21.50) | -2.67 (-2.93 to -2.41) |
| United Republic of Tanzania | 0.72 (0.36-1.28) | 1.12 (0.57-2.01) | 1.48 (1.3 to 1.66) | 24.77 (12.58-44.30) | 28.17 (14.68-49.08) | 0.57 (0.44 to 0.71) |
| United States of America | 9.00 (8.40-9.66) | 12.83 (11.81-13.95) | 1.15 (1.03 to 1.27) | 32.34 (30.31-34.73) | 30.13 (27.41-33.43) | -0.16 (-0.33 to 0) |
| United States Virgin Islands | 0.11 (0.06-0.18) | 0.80 (0.37-1.49) | 8.43 (7.82 to 9.05) | 1.59 (0.88-2.60) | 6.43 (3.13-11.39) | 6.52 (5.92 to 7.13) |
| Uruguay | 6.07 (3.95-9.05) | 17.19 (11.05-25.47) | 3.32 (3.07 to 3.58) | 73.47 (51.04-104.52) | 94.51 (66.49-128.75) | 0.79 (0.58 to 1.01) |
| Uzbekistan | 0.68 (0.44-1.03) | 1.86 (1.24-2.70) | 3.23 (2.64 to 3.82) | 13.71 (9.37-20.17) | 25.06 (18.13-34.06) | 1.7 (1.29 to 2.12) |
| Vanuatu | 0.11 (0.05-0.23) | 0.15 (0.07-0.28) | 0.69 (0.59 to 0.8) | 3.55 (1.54-6.93) | 4.24 (2.10-7.58) | 0.36 (0.28 to 0.45) |
| Venezuela (Bolivarian Republic of) | 0.55 (0.43-0.70) | 3.76 (2.49-5.51) | 6.85 (5.87 to 7.84) | 11.95 (9.98-14.16) | 38.32 (27.38-52.48) | 4.09 (3.36 to 4.82) |
| Viet Nam | 0.67 (0.35-1.18) | 1.83 (0.85-3.48) | 3.04 (2.85 to 3.23) | 14.88 (7.96-25.26) | 14.50 (7.20-26.32) | -0.33 (-0.48 to -0.19) |
| Yemen | 0.18 (0.07-0.37) | 0.70 (0.30-1.36) | 5.24 (4.76 to 5.72) | 2.50 (0.99-5.08) | 5.44 (2.44-10.42) | 3.13 (2.73 to 3.52) |
| Zambia | 0.59 (0.30-1.04) | 3.48 (1.59-6.61) | 7.43 (6.2 to 8.67) | 22.40 (11.53-39.33) | 90.48 (42.23-169.49) | 6.11 (5.07 to 7.15) |
| Zimbabwe | 0.40 (0.21-0.66) | 0.79 (0.39-1.44) | 2.07 (1.47 to 2.67) | 11.19 (6.16-18.56) | 23.22 (11.49-40.90) | 2.73 (2.18 to 3.29) |

**Table S4. Cases and age-standardized rates of incidence and DALYs in 1990 and 2021, and their estimated annual percentage changes from 1990 to 2021 for prostate cancer in adolescents and young adult males (15–49 years), globally and by 21 GBD regions.**

| **Characteristics** | **Incidence** | | | | | **DALYs** | | | | |
| --- | --- | --- | --- | --- | --- | --- | --- | --- | --- | --- |
|  | **Number of cases, 1990** | **Age-standardized rate per 100,000 population, 1990** | **Number of cases, 2021** | **Age-standardized rate per 100,000 population, 2021** | **Estimated annual percentage change, 1990–2021** | **Number of cases, 1990** | **Age-standardized rate per 100,000 population, 1990** | **Number of cases, 2021** | **Age-standardized rate per 100,000 population, 2021** | **Estimated annual percentage change, 1990–2021** |
| Andean Latin America | 39 (30-49) | 0.56 (0.40-0.76) | 191 (138-266) | 1.15 (0.78-1.71) | 2.19 (1.9 to 2.48) | 917 (689-1144) | 12.79 (9.20-17.14) | 1980 (1516-2595) | 11.84 (8.41-16.93) | -0.4 (-0.63 to -0.17) |
| Australasia | 62 (50-76) | 1.15 (0.90-1.43) | 243 (174-333) | 2.84 (2.02-3.89) | 3.1 (1.84 to 4.37) | 526 (446-623) | 9.67 (7.86-11.72) | 594 (455-785) | 7.00 (5.33-9.30) | -1.11 (-1.83 to -0.39) |
| Caribbean | 106 (91-123) | 1.46 (1.22-1.75) | 259 (206-325) | 2.11 (1.63-2.71) | 1 (0.77 to 1.23) | 1363 (1186-1591) | 18.60 (15.51-22.42) | 2626 (2068-3285) | 21.46 (16.38-28.00) | 0.35 (0.22 to 0.48) |
| Central Asia | 90 (81-99) | 0.67 (0.60-0.75) | 197 (170-228) | 0.79 (0.68-0.94) | 0.96 (0.68 to 1.23) | 2308 (2136-2480) | 16.47 (15.06-17.96) | 3393 (2943-3945) | 13.64 (11.75-16.10) | -0.42 (-0.65 to -0.19) |
| Central Europe | 168 (153-186) | 0.54 (0.49-0.60) | 356 (312-406) | 0.96 (0.83-1.10) | 1.78 (1.53 to 2.04) | 3003 (2805-3234) | 9.62 (8.88-10.49) | 3094 (2782-3424) | 8.45 (7.57-9.46) | -0.76 (-1.03 to -0.49) |
| Central Latin America | 329 (301-357) | 1.12 (1.01-1.25) | 1336 (1123-1557) | 2.06 (1.72-2.45) | 1.44 (1.05 to 1.82) | 3716 (3520-3913) | 12.34 (11.36-13.46) | 7618 (6642-8757) | 11.77 (10.10-13.70) | -0.58 (-0.79 to -0.37) |
| Central Sub-Saharan Africa | 28 (15-40) | 0.36 (0.19-0.55) | 130 (73-192) | 0.54 (0.30-0.85) | 1.41 (1.2 to 1.61) | 986 (531-1389) | 12.18 (6.39-19.22) | 3394 (1901-4950) | 13.85 (7.67-22.16) | 0.45 (0.32 to 0.59) |
| East Asia | 733 (350-1001) | 0.24 (0.12-0.34) | 2537 (1749-3421) | 0.57 (0.38-0.79) | 2.75 (2.61 to 2.88) | 18120 (8202-24913) | 5.90 (2.71-8.26) | 20992 (14081-28081) | 4.82 (3.12-6.65) | -1.01 (-1.24 to -0.79) |
| Eastern Europe | 574 (532-615) | 1.14 (1.04-1.24) | 1158 (1027-1281) | 1.94 (1.70-2.18) | 1.69 (1.45 to 1.94) | 7779 (7143-8374) | 15.21 (13.66-16.89) | 8439 (7348-9623) | 14.44 (12.32-17.07) | -0.39 (-0.74 to -0.04) |
| Eastern Sub-Saharan Africa | 82 (47-111) | 0.31 (0.17-0.43) | 349 (215-488) | 0.49 (0.29-0.70) | 1.45 (1.38 to 1.53) | 2697 (1537-3703) | 10.05 (5.56-14.10) | 8433 (5146-11746) | 11.82 (7.05-16.84) | 0.43 (0.37 to 0.48) |
| High-income Asia Pacific | 76 (66-89) | 0.15 (0.13-0.18) | 214 (171-262) | 0.37 (0.29-0.47) | 3.15 (2.56 to 3.73) | 1443 (1271-1594) | 2.87 (2.49-3.34) | 1403 (1248-1603) | 2.55 (2.24-3.02) | -0.7 (-0.97 to -0.43) |
| High-income North America | 1922 (1819-2043) | 2.68 (2.52-2.85) | 3606 (3344-3906) | 3.75 (3.47-4.07) | 0.5 (0.09 to 0.91) | 7402 (6928-7955) | 10.22 (9.52-11.02) | 8822 (7903-9829) | 9.24 (8.26-10.32) | -0.84 (-1.06 to -0.62) |
| North Africa and Middle East | 215 (135-269) | 0.36 (0.22-0.47) | 1509 (1028-1875) | 0.85 (0.56-1.12) | 3.07 (2.94 to 3.19) | 3097 (1871-3793) | 5.03 (3.04-6.66) | 8940 (5781-10784) | 5.00 (3.16-6.40) | 0.11 (0.03 to 0.19) |
| Oceania | 6 (4-9) | 0.50 (0.31-0.79) | 22 (13-33) | 0.70 (0.40-1.15) | 1.21 (1.16 to 1.25) | 175 (110-261) | 13.65 (7.98-22.30) | 548 (322-835) | 17.00 (9.24-29.25) | 0.85 (0.77 to 0.93) |
| South Asia | 190 (128-235) | 0.09 (0.06-0.11) | 727 (570-1030) | 0.16 (0.12-0.22) | 1.82 (1.73 to 1.91) | 5881 (4006-7308) | 2.68 (1.79-3.40) | 13004 (10240-18304) | 2.80 (2.19-4.01) | -0.05 (-0.13 to 0.04) |
| Southeast Asia | 221 (142-270) | 0.25 (0.16-0.32) | 948 (587-1229) | 0.48 (0.29-0.64) | 2.05 (1.97 to 2.13) | 5677 (3742-6787) | 6.41 (4.09-7.94) | 14775 (9403-19112) | 7.53 (4.62-9.99) | 0.45 (0.38 to 0.53) |
| Southern Latin America | 77 (64-91) | 0.68 (0.54-0.85) | 193 (150-241) | 1.05 (0.78-1.39) | 1.77 (1.37 to 2.17) | 1549 (1341-1781) | 13.67 (11.08-16.78) | 1732 (1452-2066) | 9.49 (7.30-12.03) | -0.93 (-1.15 to -0.71) |
| Southern Sub-Saharan Africa | 73 (57-92) | 0.83 (0.64-1.10) | 282 (217-404) | 1.48 (1.11-2.16) | 1.97 (1.87 to 2.08) | 1970 (1553-2511) | 22.13 (16.97-29.01) | 5369 (4111-7238) | 27.97 (20.70-39.24) | 0.78 (0.62 to 0.95) |
| Tropical Latin America | 243 (223-267) | 0.80 (0.72-0.89) | 890 (805-987) | 1.40 (1.25-1.58) | 1.45 (1.04 to 1.87) | 4709 (4378-5001) | 15.13 (13.83-16.48) | 9550 (8871-10222) | 15.11 (13.78-16.57) | -0.26 (-0.58 to 0.06) |
| Western Europe | 1193 (1093-1317) | 1.16 (1.04-1.29) | 2088 (1863-2357) | 1.64 (1.43-1.89) | 1.14 (0.67 to 1.6) | 9058 (8552-9611) | 8.80 (8.15-9.54) | 6826 (6100-7670) | 5.47 (4.82-6.26) | -1.74 (-1.94 to -1.54) |
| Western Sub-Saharan Africa | 142 (86-190) | 0.44 (0.26-0.60) | 631 (334-893) | 0.81 (0.42-1.14) | 2.11 (2 to 2.22) | 4506 (2712-6017) | 13.97 (8.29-19.11) | 14233 (7472-19725) | 18.08 (9.28-24.96) | 0.93 (0.84 to 1.02) |

**Table S5. Age-standardized rates of incidence and DALYs in 2021, and their estimated annual percentage changes from 1990 to 2021 for prostate cancer in adolescents and young adult males (15–49 years), by country.**

| **Location name** | **Age-standardized incidence rate, 1990** | **Age-standardized incidence rate, 2021** | **EAPC of incidence rate** | **Age-standardized DALY rate, 1990** | **Age-standardized DALY rate, 2021** | **EAPC of DALY rate** |
| --- | --- | --- | --- | --- | --- | --- |
| Afghanistan | 0.19 (0.09-0.36) | 0.35 (0.16-0.68) | 2.17 (1.88 to 2.45) | 4.78 (2.27-8.80) | 5.98 (2.70-11.50) | 0.81 (0.7 to 0.92) |
| Albania | 0.25 (0.14-0.45) | 0.59 (0.33-1.01) | 3.45 (3.07 to 3.83) | 5.82 (3.30-10.16) | 5.84 (3.44-9.67) | 0.24 (0.05 to 0.42) |
| Algeria | 0.07 (0.04-0.12) | 0.15 (0.08-0.27) | 2.28 (2.21 to 2.36) | 0.97 (0.58-1.53) | 0.92 (0.52-1.52) | -0.35 (-0.43 to -0.27) |
| American Samoa | 1.64 (0.91-2.80) | 2.48 (1.39-4.26) | 1.79 (1.03 to 2.55) | 36.62 (20.36-62.04) | 44.28 (25.56-74.72) | 1.08 (0.36 to 1.81) |
| Andorra | 1.21 (0.67-2.07) | 1.98 (0.97-3.57) | 2.1 (1.67 to 2.53) | 8.47 (4.98-13.87) | 6.72 (3.33-11.48) | -0.3 (-0.52 to -0.09) |
| Angola | 0.35 (0.18-0.60) | 0.60 (0.31-1.06) | 1.83 (1.64 to 2.01) | 12.48 (6.13-21.59) | 15.06 (7.91-26.15) | 0.7 (0.58 to 0.81) |
| Antigua and Barbuda | 2.58 (1.93-3.39) | 2.99 (2.13-4.07) | 1.02 (0.68 to 1.36) | 29.49 (24.49-35.24) | 23.70 (18.89-29.87) | -0.09 (-0.38 to 0.21) |
| Argentina | 0.65 (0.49-0.85) | 0.91 (0.63-1.28) | 1.48 (1.05 to 1.91) | 13.45 (10.59-16.89) | 9.44 (7.02-12.29) | -0.8 (-1.07 to -0.53) |
| Armenia | 1.15 (0.96-1.38) | 1.80 (1.38-2.29) | 2.07 (1.38 to 2.77) | 25.23 (22.98-27.58) | 22.99 (19.83-26.28) | -0.09 (-0.72 to 0.55) |
| Australia | 1.01 (0.76-1.32) | 2.91 (1.96-4.15) | 3.51 (2.05 to 5) | 9.79 (7.75-12.15) | 7.06 (5.14-9.59) | -1.12 (-1.94 to -0.29) |
| Austria | 2.17 (1.53-3.09) | 2.05 (1.39-2.89) | -0.21 (-0.63 to 0.22) | 10.49 (8.02-13.36) | 5.10 (3.67-6.78) | -2.34 (-2.5 to -2.18) |
| Azerbaijan | 0.80 (0.55-1.08) | 0.97 (0.61-1.50) | 0.9 (0.55 to 1.25) | 22.15 (15.81-29.25) | 17.34 (11.22-26.29) | -0.78 (-0.99 to -0.58) |
| Bahamas | 3.35 (2.51-4.40) | 4.97 (3.40-7.01) | 1.25 (1.1 to 1.39) | 44.30 (36.87-53.38) | 49.89 (37.57-65.48) | 0.24 (0.15 to 0.34) |
| Bahrain | 0.59 (0.33-0.96) | 1.38 (0.67-2.63) | 2.75 (2.61 to 2.89) | 6.39 (3.80-10.23) | 6.11 (3.06-11.11) | -0.51 (-0.71 to -0.3) |
| Bangladesh | 0.09 (0.04-0.15) | 0.14 (0.05-0.31) | 1.47 (1.34 to 1.59) | 2.80 (1.36-4.80) | 2.41 (0.88-4.96) | -0.77 (-0.87 to -0.67) |
| Barbados | 2.42 (1.64-3.46) | 3.01 (1.79-4.73) | 0.84 (0.6 to 1.08) | 26.58 (19.55-35.71) | 23.14 (14.71-34.66) | -0.23 (-0.4 to -0.06) |
| Belarus | 0.88 (0.63-1.19) | 2.92 (1.88-4.32) | 3.7 (3.01 to 4.39) | 10.30 (7.98-13.06) | 19.42 (13.54-27.28) | 1.58 (0.85 to 2.31) |
| Belgium | 1.13 (0.81-1.56) | 1.61 (1.08-2.34) | 0.59 (-0.03 to 1.21) | 9.25 (7.14-11.83) | 6.05 (4.43-7.98) | -2.01 (-2.44 to -1.58) |
| Belize | 0.72 (0.55-0.93) | 2.14 (1.59-2.89) | 2.82 (2.25 to 3.38) | 10.93 (9.14-12.86) | 24.08 (19.76-29.37) | 1.78 (1.11 to 2.46) |
| Benin | 0.28 (0.17-0.44) | 0.58 (0.27-1.06) | 2.6 (2.5 to 2.7) | 9.04 (5.51-14.27) | 13.91 (6.66-24.93) | 1.58 (1.49 to 1.68) |
| Bermuda | 2.68 (1.85-3.76) | 4.68 (2.94-6.98) | 1.75 (1.48 to 2.02) | 21.92 (16.29-28.36) | 17.50 (12.18-24.60) | -0.76 (-0.88 to -0.64) |
| Bhutan | 0.07 (0.03-0.14) | 0.13 (0.05-0.28) | 1.85 (1.73 to 1.96) | 2.38 (1.04-4.33) | 2.26 (0.83-4.92) | -0.21 (-0.27 to -0.16) |
| Bolivia (Plurinational State of) | 0.55 (0.32-0.88) | 0.91 (0.47-1.61) | 1.5 (1.41 to 1.6) | 15.33 (9.24-24.73) | 15.07 (7.82-27.05) | -0.23 (-0.31 to -0.14) |
| Bosnia and Herzegovina | 0.32 (0.22-0.47) | 0.59 (0.32-0.92) | 2.77 (2.39 to 3.14) | 6.28 (4.45-8.81) | 5.78 (3.37-8.52) | 0.01 (-0.15 to 0.17) |
| Botswana | 0.76 (0.40-1.37) | 1.15 (0.59-2.00) | 1.07 (0.88 to 1.25) | 23.09 (12.10-41.30) | 24.19 (12.33-43.55) | -0.21 (-0.49 to 0.08) |
| Brazil | 0.80 (0.72-0.90) | 1.41 (1.25-1.59) | 1.43 (1.01 to 1.85) | 15.28 (13.95-16.69) | 15.18 (13.84-16.67) | -0.28 (-0.61 to 0.04) |
| Brunei Darussalam | 0.20 (0.11-0.33) | 0.34 (0.20-0.56) | 2.04 (1.96 to 2.12) | 6.18 (3.49-10.48) | 6.20 (3.70-9.94) | 0.08 (-0.07 to 0.23) |
| Bulgaria | 0.39 (0.31-0.49) | 0.75 (0.57-0.97) | 1.3 (0.86 to 1.74) | 8.00 (6.89-9.15) | 9.54 (7.87-11.26) | -0.39 (-0.89 to 0.12) |
| Burkina Faso | 0.26 (0.15-0.44) | 0.54 (0.25-0.99) | 2.71 (2.6 to 2.81) | 8.36 (4.78-13.81) | 13.73 (6.34-24.94) | 1.88 (1.76 to 2) |
| Burundi | 0.42 (0.16-0.77) | 0.42 (0.18-0.79) | -0.26 (-0.41 to -0.1) | 14.43 (5.47-26.53) | 11.48 (5.04-21.83) | -1.03 (-1.19 to -0.87) |
| Cabo Verde | 0.57 (0.30-1.05) | 2.13 (1.10-3.75) | 4.51 (4.03 to 4.98) | 14.70 (8.05-26.44) | 29.49 (15.62-50.17) | 2.17 (1.7 to 2.65) |
| Cambodia | 0.21 (0.12-0.35) | 0.41 (0.21-0.72) | 2.36 (2.25 to 2.46) | 6.90 (4.00-11.57) | 8.47 (4.43-15.23) | 0.79 (0.74 to 0.84) |
| Cameroon | 0.34 (0.21-0.51) | 0.80 (0.36-1.43) | 2.99 (2.89 to 3.1) | 10.46 (6.54-15.96) | 18.28 (7.99-33.08) | 2.01 (1.88 to 2.14) |
| Canada | 1.63 (1.15-2.28) | 1.78 (1.21-2.52) | -0.53 (-1.07 to 0.01) | 8.50 (6.78-10.51) | 5.96 (4.49-7.81) | -1.98 (-2.4 to -1.56) |
| Central African Republic | 0.38 (0.17-0.72) | 0.44 (0.19-0.85) | 0.4 (0.34 to 0.46) | 13.92 (6.26-26.83) | 14.49 (6.11-27.57) | 0.04 (-0.03 to 0.11) |
| Chad | 0.21 (0.11-0.35) | 0.50 (0.24-0.91) | 3.14 (2.97 to 3.32) | 7.02 (3.78-11.66) | 13.95 (6.77-24.86) | 2.51 (2.31 to 2.72) |
| Chile | 0.71 (0.52-0.95) | 1.29 (0.86-1.82) | 2.38 (2.01 to 2.76) | 13.90 (10.65-18.06) | 8.82 (6.50-11.41) | -1.26 (-1.44 to -1.07) |
| China | 0.24 (0.11-0.34) | 0.55 (0.36-0.78) | 2.78 (2.62 to 2.94) | 5.90 (2.64-8.32) | 4.72 (3.02-6.59) | -1.07 (-1.3 to -0.85) |
| Colombia | 1.40 (1.00-1.90) | 2.13 (1.36-3.20) | 0.64 (0.12 to 1.18) | 14.71 (11.21-18.68) | 9.48 (6.62-13.32) | -2.01 (-2.39 to -1.62) |
| Comoros | 0.43 (0.13-0.86) | 0.59 (0.27-1.11) | 0.7 (0.47 to 0.94) | 14.04 (4.45-27.48) | 14.17 (6.60-26.22) | -0.37 (-0.67 to -0.07) |
| Congo | 0.50 (0.22-1.03) | 0.70 (0.34-1.23) | 1.01 (0.83 to 1.2) | 16.75 (7.36-34.26) | 16.48 (7.93-29.05) | -0.24 (-0.39 to -0.09) |
| Cook Islands | 2.36 (1.32-3.85) | 5.01 (2.77-8.48) | 2.79 (2.64 to 2.93) | 44.14 (25.50-72.10) | 46.60 (26.74-77.31) | 0.48 (0.32 to 0.64) |
| Costa Rica | 1.33 (0.94-1.85) | 4.15 (2.63-6.03) | 3.24 (3.02 to 3.47) | 8.08 (6.27-10.36) | 15.66 (11.42-21.18) | 1.68 (1.47 to 1.9) |
| Croatia | 0.81 (0.58-1.11) | 1.19 (0.81-1.69) | 1.15 (0.78 to 1.53) | 9.58 (7.45-12.26) | 7.54 (5.54-10.02) | -1.01 (-1.37 to -0.65) |
| Cuba | 1.71 (1.20-2.36) | 2.53 (1.67-3.63) | 0.89 (0.69 to 1.1) | 15.33 (11.77-19.83) | 14.47 (10.24-19.62) | -0.45 (-0.6 to -0.29) |
| Cyprus | 0.51 (0.29-0.85) | 1.59 (0.85-2.79) | 4.28 (3.82 to 4.74) | 5.35 (3.17-8.57) | 5.87 (3.35-9.55) | 0.34 (0.19 to 0.49) |
| Czechia | 0.73 (0.55-0.96) | 1.19 (0.80-1.67) | 1.85 (1.48 to 2.22) | 9.16 (7.41-11.27) | 6.99 (5.15-9.20) | -0.62 (-0.86 to -0.37) |
| Côte d’Ivoire | 0.63 (0.37-1.02) | 1.05 (0.55-1.82) | 1.52 (1.43 to 1.6) | 19.72 (11.36-31.68) | 23.54 (12.59-40.33) | 0.41 (0.28 to 0.53) |
| Democratic People's Republic of Korea | 0.26 (0.14-0.46) | 0.44 (0.22-0.79) | 1.82 (1.7 to 1.94) | 5.96 (3.15-10.31) | 6.52 (3.46-11.66) | 0.28 (0.25 to 0.32) |
| Democratic Republic of the Congo | 0.34 (0.18-0.57) | 0.50 (0.27-0.88) | 1.33 (1.08 to 1.57) | 11.47 (6.09-20.30) | 13.05 (7.03-23.01) | 0.49 (0.31 to 0.66) |
| Denmark | 0.80 (0.59-1.08) | 1.21 (0.82-1.73) | 1.51 (0.97 to 2.04) | 10.39 (8.20-13.07) | 6.00 (4.54-7.92) | -2.17 (-2.51 to -1.83) |
| Djibouti | 0.47 (0.18-0.92) | 0.66 (0.31-1.27) | 0.97 (0.9 to 1.03) | 14.69 (5.66-28.68) | 15.24 (6.67-29.01) | -0.06 (-0.18 to 0.07) |
| Dominica | 1.68 (0.94-2.75) | 2.61 (1.44-4.48) | 1.25 (1.06 to 1.44) | 25.12 (14.35-40.09) | 32.23 (18.78-54.00) | 0.83 (0.64 to 1.02) |
| Dominican Republic | 1.00 (0.61-1.56) | 1.70 (0.88-3.15) | 1.83 (1.57 to 2.1) | 18.26 (11.47-28.29) | 20.70 (10.96-36.86) | 0.73 (0.6 to 0.86) |
| Ecuador | 0.53 (0.39-0.71) | 1.07 (0.67-1.63) | 1.83 (1.06 to 2.6) | 11.67 (8.95-15.11) | 11.96 (7.89-17.18) | -0.19 (-0.86 to 0.48) |
| Egypt | 0.19 (0.11-0.31) | 0.53 (0.25-0.94) | 3.21 (3.06 to 3.36) | 3.18 (1.90-5.05) | 4.08 (2.00-7.02) | 0.86 (0.76 to 0.97) |
| El Salvador | 1.18 (0.76-1.73) | 3.59 (2.00-5.82) | 3.62 (3.34 to 3.9) | 15.28 (10.45-21.04) | 19.84 (11.22-29.76) | 0.84 (0.71 to 0.97) |
| Equatorial Guinea | 0.41 (0.19-0.76) | 0.91 (0.43-1.67) | 2.94 (2.76 to 3.11) | 14.45 (6.81-27.20) | 16.79 (7.91-31.00) | 0.46 (0.34 to 0.59) |
| Eritrea | 0.45 (0.17-0.85) | 0.59 (0.27-1.10) | 0.69 (0.58 to 0.79) | 15.67 (5.74-30.13) | 16.23 (6.90-30.41) | -0.06 (-0.16 to 0.04) |
| Estonia | 2.03 (1.46-2.79) | 4.03 (2.67-5.89) | 1.64 (1.29 to 1.99) | 20.50 (15.96-26.17) | 19.97 (14.58-27.29) | -1.12 (-1.55 to -0.69) |
| Eswatini | 0.70 (0.40-1.18) | 1.30 (0.57-2.49) | 2.08 (1.8 to 2.37) | 21.11 (11.51-35.73) | 30.70 (13.67-60.81) | 1.34 (0.97 to 1.72) |
| Ethiopia | 0.12 (0.06-0.20) | 0.17 (0.08-0.28) | 0.84 (0.64 to 1.05) | 4.42 (2.37-7.20) | 3.89 (1.84-6.57) | -0.59 (-0.72 to -0.46) |
| Fiji | 0.54 (0.20-1.01) | 0.70 (0.23-1.41) | 1.16 (0.88 to 1.45) | 14.09 (5.28-26.32) | 15.06 (4.97-31.07) | 0.64 (0.33 to 0.95) |
| Finland | 1.27 (0.91-1.72) | 2.15 (1.44-3.01) | 1.96 (1.38 to 2.53) | 10.99 (8.48-13.73) | 6.68 (4.90-9.06) | -1.76 (-2.04 to -1.47) |
| France | 1.47 (1.05-2.06) | 2.60 (1.76-3.70) | 1.94 (1.48 to 2.4) | 10.57 (8.44-13.34) | 6.94 (4.97-9.47) | -1.55 (-1.79 to -1.32) |
| Gabon | 0.54 (0.26-0.97) | 0.93 (0.42-1.74) | 1.7 (1.59 to 1.81) | 16.79 (8.38-29.04) | 18.56 (8.40-34.26) | 0.18 (0.1 to 0.27) |
| Gambia | 0.09 (0.05-0.14) | 0.17 (0.09-0.29) | 1.82 (1.62 to 2.02) | 2.69 (1.59-4.30) | 3.73 (2.09-6.48) | 0.79 (0.57 to 1) |
| Georgia | 1.51 (1.21-1.87) | 2.32 (1.80-2.98) | 1.98 (1.66 to 2.3) | 30.79 (26.83-35.54) | 35.94 (30.28-41.94) | 1.13 (0.77 to 1.49) |
| Germany | 1.07 (0.78-1.42) | 1.59 (1.10-2.22) | 1.08 (0.46 to 1.69) | 8.76 (7.20-10.99) | 5.56 (4.25-7.27) | -1.97 (-2.25 to -1.68) |
| Ghana | 0.62 (0.34-1.06) | 0.96 (0.55-1.64) | 1.16 (0.95 to 1.37) | 18.98 (10.19-32.36) | 20.42 (11.72-33.25) | 0.04 (-0.15 to 0.23) |
| Greece | 1.02 (0.76-1.36) | 1.27 (0.92-1.71) | 0.53 (0.08 to 0.98) | 7.46 (6.38-8.76) | 5.33 (4.42-6.40) | -1.27 (-1.45 to -1.09) |
| Greenland | 0.45 (0.26-0.71) | 0.45 (0.25-0.76) | 0.48 (0.26 to 0.69) | 8.24 (5.11-12.44) | 4.29 (2.53-6.90) | -1.86 (-2.1 to -1.63) |
| Grenada | 2.61 (1.81-3.69) | 3.19 (2.10-4.58) | 0.8 (0.21 to 1.39) | 41.64 (30.03-55.54) | 33.75 (23.90-46.07) | -0.29 (-0.78 to 0.21) |
| Guam | 0.92 (0.58-1.41) | 2.03 (1.25-2.97) | 3.09 (2.62 to 3.56) | 14.66 (9.48-21.92) | 24.22 (15.89-34.11) | 2 (1.63 to 2.36) |
| Guatemala | 0.87 (0.69-1.11) | 1.71 (1.23-2.31) | 1.46 (0.72 to 2.21) | 15.42 (13.24-17.88) | 14.93 (11.84-18.27) | -0.72 (-1.26 to -0.17) |
| Guinea | 0.21 (0.12-0.33) | 0.37 (0.21-0.61) | 1.88 (1.8 to 1.97) | 6.93 (3.96-10.90) | 9.80 (5.58-15.90) | 1.17 (1.09 to 1.26) |
| Guinea-Bissau | 0.33 (0.17-0.62) | 0.70 (0.32-1.32) | 2.78 (2.65 to 2.9) | 11.58 (6.01-21.46) | 19.69 (8.90-37.00) | 2.03 (1.91 to 2.16) |
| Guyana | 1.20 (0.83-1.66) | 2.65 (1.64-4.00) | 2.4 (2.09 to 2.72) | 25.87 (18.48-34.49) | 43.10 (27.80-63.92) | 1.71 (1.4 to 2.02) |
| Haiti | 0.85 (0.47-1.36) | 1.13 (0.60-1.91) | 1.02 (0.95 to 1.1) | 24.49 (13.84-38.87) | 26.87 (14.41-44.96) | 0.45 (0.39 to 0.52) |
| Honduras | 0.55 (0.32-0.91) | 0.89 (0.42-1.60) | 1.3 (1.11 to 1.49) | 8.68 (5.25-13.98) | 8.74 (4.40-15.43) | -0.17 (-0.29 to -0.06) |
| Hungary | 0.82 (0.60-1.12) | 1.08 (0.73-1.54) | -0.01 (-0.62 to 0.6) | 13.78 (10.53-17.62) | 8.67 (6.43-11.45) | -2.36 (-2.81 to -1.91) |
| Iceland | 1.36 (0.96-1.92) | 2.00 (1.30-2.92) | 0.59 (0.26 to 0.91) | 9.06 (7.05-11.54) | 7.41 (5.42-9.92) | -1.25 (-1.47 to -1.02) |
| India | 0.08 (0.05-0.11) | 0.15 (0.11-0.20) | 1.82 (1.7 to 1.93) | 2.49 (1.57-3.24) | 2.49 (1.92-3.47) | -0.21 (-0.31 to -0.11) |
| Indonesia | 0.21 (0.13-0.29) | 0.42 (0.24-0.64) | 2.15 (2.1 to 2.2) | 6.08 (3.81-8.25) | 7.87 (4.53-12.07) | 0.86 (0.76 to 0.96) |
| Iran (Islamic Republic of) | 0.55 (0.23-0.84) | 1.33 (0.56-2.03) | 2.94 (2.8 to 3.09) | 7.26 (3.17-10.30) | 7.80 (3.27-10.90) | 0.48 (0.36 to 0.61) |
| Iraq | 0.23 (0.12-0.39) | 0.53 (0.27-0.90) | 3.14 (2.94 to 3.34) | 2.92 (1.66-4.98) | 3.14 (1.74-5.37) | 0.32 (0.21 to 0.43) |
| Ireland | 0.90 (0.63-1.22) | 1.44 (0.98-2.07) | 2.09 (1.52 to 2.67) | 7.85 (5.95-10.14) | 4.81 (3.49-6.45) | -1.46 (-1.78 to -1.14) |
| Israel | 0.42 (0.29-0.58) | 0.65 (0.44-0.91) | 1.43 (0.6 to 2.27) | 4.37 (3.31-5.73) | 2.79 (2.05-3.67) | -1.7 (-2.27 to -1.13) |
| Italy | 1.21 (0.93-1.58) | 1.23 (0.90-1.65) | 0.05 (-0.41 to 0.51) | 7.76 (7.06-8.57) | 4.04 (3.47-4.76) | -2.05 (-2.3 to -1.81) |
| Jamaica | 1.03 (0.71-1.40) | 2.44 (1.45-3.83) | 2.25 (1.23 to 3.28) | 11.78 (8.89-15.38) | 21.69 (13.63-32.23) | 1.44 (0.48 to 2.4) |
| Japan | 0.16 (0.14-0.19) | 0.40 (0.31-0.51) | 3.28 (2.61 to 3.96) | 2.73 (2.61-2.87) | 2.75 (2.59-2.97) | -0.26 (-0.61 to 0.1) |
| Jordan | 0.33 (0.18-0.55) | 0.87 (0.46-1.52) | 3.97 (3.62 to 4.33) | 3.65 (2.14-5.75) | 4.20 (2.41-6.84) | 0.77 (0.59 to 0.95) |
| Kazakhstan | 0.85 (0.71-1.03) | 1.11 (0.87-1.43) | 1.49 (0.86 to 2.12) | 21.01 (18.67-23.46) | 15.95 (14.03-17.90) | -0.66 (-1.5 to 0.18) |
| Kenya | 0.20 (0.10-0.31) | 0.38 (0.24-0.55) | 2.04 (1.87 to 2.21) | 5.47 (2.83-8.59) | 7.93 (4.92-11.57) | 1.24 (1.02 to 1.47) |
| Kiribati | 0.38 (0.20-0.65) | 0.50 (0.25-0.88) | 0.88 (0.83 to 0.92) | 13.16 (6.79-23.08) | 14.76 (7.26-26.30) | 0.39 (0.33 to 0.45) |
| Kuwait | 0.33 (0.23-0.46) | 1.73 (1.10-2.60) | 4.35 (3.46 to 5.24) | 1.88 (1.43-2.47) | 5.78 (3.84-8.34) | 2.11 (1.2 to 3.03) |
| Kyrgyzstan | 0.66 (0.47-0.89) | 0.97 (0.66-1.37) | 1.43 (1.09 to 1.77) | 17.75 (12.97-23.38) | 16.72 (11.84-22.41) | -0.19 (-0.42 to 0.05) |
| Lao People's Democratic Republic | 0.19 (0.11-0.33) | 0.29 (0.15-0.52) | 1.36 (1.31 to 1.4) | 6.70 (3.69-11.64) | 6.88 (3.60-12.01) | 0.08 (0.04 to 0.13) |
| Latvia | 1.34 (0.94-1.84) | 4.08 (2.62-5.83) | 3.05 (2.53 to 3.58) | 15.05 (11.74-18.86) | 29.33 (21.02-38.84) | 1.66 (1.22 to 2.1) |
| Lebanon | 0.57 (0.29-1.08) | 1.55 (0.85-2.59) | 3.9 (3.68 to 4.13) | 5.66 (3.01-10.22) | 5.96 (3.40-9.55) | 0.54 (0.4 to 0.68) |
| Lesotho | 0.53 (0.28-0.92) | 1.03 (0.51-1.96) | 2.12 (1.88 to 2.36) | 16.58 (8.62-28.66) | 28.01 (13.92-52.34) | 1.77 (1.5 to 2.04) |
| Liberia | 0.24 (0.14-0.41) | 0.63 (0.28-1.22) | 3.6 (3.39 to 3.81) | 8.05 (4.49-13.56) | 14.06 (6.07-27.81) | 2.22 (2.04 to 2.4) |
| Libya | 0.49 (0.24-0.83) | 1.02 (0.53-1.84) | 2.86 (2.53 to 3.2) | 5.26 (2.82-8.73) | 6.37 (3.44-11.07) | 0.74 (0.63 to 0.86) |
| Lithuania | 2.09 (1.48-2.87) | 6.39 (4.26-9.19) | 3.53 (3.16 to 3.9) | 15.26 (11.79-19.16) | 31.85 (23.21-41.77) | 2.47 (2.04 to 2.9) |
| Luxembourg | 1.06 (0.80-1.39) | 1.00 (0.71-1.36) | -0.25 (-0.8 to 0.31) | 9.92 (8.43-11.77) | 3.58 (2.85-4.51) | -3.53 (-3.79 to -3.28) |
| Madagascar | 0.36 (0.15-0.64) | 0.39 (0.19-0.75) | 0.1 (0.01 to 0.2) | 11.78 (4.77-20.57) | 10.06 (4.75-19.10) | -0.68 (-0.77 to -0.58) |
| Malawi | 0.26 (0.15-0.42) | 0.51 (0.29-0.85) | 2.14 (2.03 to 2.26) | 8.69 (4.99-13.66) | 12.65 (7.40-20.41) | 1.14 (0.99 to 1.28) |
| Malaysia | 0.17 (0.10-0.30) | 0.38 (0.21-0.64) | 2.83 (2.72 to 2.95) | 3.88 (2.20-6.65) | 4.59 (2.66-7.47) | 0.62 (0.55 to 0.69) |
| Maldives | 0.14 (0.07-0.24) | 0.34 (0.16-0.69) | 3.43 (2.98 to 3.89) | 3.70 (1.83-6.55) | 3.49 (1.79-6.85) | -0.04 (-0.32 to 0.23) |
| Mali | 0.15 (0.09-0.23) | 0.23 (0.13-0.39) | 1.62 (1.54 to 1.71) | 4.76 (2.92-7.34) | 5.68 (3.15-9.21) | 0.69 (0.58 to 0.8) |
| Malta | 0.48 (0.34-0.66) | 1.00 (0.68-1.46) | 2.28 (2.01 to 2.56) | 4.17 (3.18-5.40) | 3.81 (2.78-5.18) | -0.45 (-0.67 to -0.23) |
| Marshall Islands | 0.61 (0.34-1.02) | 0.96 (0.49-1.68) | 1.6 (1.52 to 1.68) | 18.81 (10.32-31.14) | 24.14 (12.29-40.75) | 0.94 (0.86 to 1.01) |
| Mauritania | 0.31 (0.17-0.50) | 0.84 (0.37-1.60) | 3.36 (3.24 to 3.48) | 9.76 (5.63-15.50) | 15.41 (6.90-29.53) | 1.59 (1.47 to 1.7) |
| Mauritius | 0.42 (0.33-0.52) | 1.22 (0.93-1.58) | 2.43 (2.03 to 2.82) | 8.06 (6.84-9.41) | 14.79 (12.44-17.33) | 1.05 (0.66 to 1.45) |
| Mexico | 1.00 (0.92-1.07) | 1.71 (1.41-2.03) | 1.14 (0.82 to 1.46) | 11.12 (10.52-11.77) | 10.24 (8.60-12.16) | -0.68 (-0.83 to -0.53) |
| Micronesia (Federated States of) | 0.66 (0.37-1.11) | 1.06 (0.55-1.86) | 1.68 (1.62 to 1.75) | 20.45 (10.97-34.12) | 24.55 (12.25-42.72) | 0.66 (0.62 to 0.7) |
| Monaco | 1.25 (0.69-2.08) | 2.61 (1.41-4.52) | 2.5 (2.19 to 2.8) | 8.37 (4.76-13.20) | 9.48 (5.16-15.90) | 0.38 (0.26 to 0.49) |
| Mongolia | 0.16 (0.08-0.28) | 0.41 (0.24-0.66) | 3.58 (3.36 to 3.79) | 5.54 (2.81-9.78) | 8.55 (5.11-13.84) | 1.66 (1.5 to 1.82) |
| Montenegro | 0.97 (0.61-1.46) | 1.52 (0.90-2.39) | 1.84 (1.53 to 2.14) | 11.26 (7.72-15.45) | 12.01 (7.74-18.35) | 0.12 (-0.09 to 0.33) |
| Morocco | 0.10 (0.05-0.16) | 0.21 (0.10-0.38) | 2.43 (2.33 to 2.53) | 1.57 (0.93-2.59) | 1.67 (0.92-2.98) | 0.14 (0.09 to 0.19) |
| Mozambique | 0.10 (0.05-0.18) | 0.20 (0.10-0.38) | 2.5 (2.4 to 2.6) | 3.46 (1.80-6.36) | 5.49 (2.73-10.25) | 1.88 (1.76 to 2) |
| Myanmar | 0.18 (0.10-0.30) | 0.29 (0.16-0.50) | 1.55 (1.49 to 1.61) | 5.79 (3.07-9.75) | 6.11 (3.35-10.50) | 0.09 (0.03 to 0.15) |
| Namibia | 0.59 (0.31-1.05) | 1.12 (0.59-1.89) | 1.91 (1.72 to 2.1) | 17.74 (9.31-31.36) | 22.16 (11.38-37.62) | 0.46 (0.2 to 0.73) |
| Nauru | 0.75 (0.36-1.53) | 1.12 (0.50-2.42) | 1.31 (1.19 to 1.43) | 21.27 (10.19-43.59) | 25.20 (11.15-53.49) | 0.62 (0.56 to 0.69) |
| Nepal | 0.07 (0.03-0.12) | 0.12 (0.05-0.24) | 1.91 (1.73 to 2.09) | 2.17 (1.13-3.78) | 2.28 (0.95-4.49) | 0.18 (0 to 0.36) |
| Netherlands | 0.84 (0.61-1.14) | 1.15 (0.80-1.63) | 1.27 (0.8 to 1.74) | 6.91 (5.46-8.69) | 4.41 (3.39-5.75) | -1.56 (-1.85 to -1.26) |
| New Zealand | 1.87 (1.32-2.63) | 2.52 (1.78-3.48) | 0.93 (0.79 to 1.07) | 9.05 (7.14-11.31) | 6.74 (5.24-8.77) | -1.12 (-1.26 to -0.97) |
| Nicaragua | 0.82 (0.48-1.32) | 1.64 (0.91-2.71) | 2.36 (2.17 to 2.56) | 9.43 (5.86-14.38) | 10.08 (5.96-15.84) | 0.2 (0.02 to 0.38) |
| Niger | 0.21 (0.11-0.36) | 0.42 (0.16-0.83) | 2.74 (2.59 to 2.9) | 6.96 (3.76-12.27) | 11.14 (4.23-22.78) | 1.85 (1.73 to 1.97) |
| Nigeria | 0.52 (0.26-0.78) | 0.94 (0.38-1.53) | 2.16 (1.98 to 2.34) | 16.35 (8.09-24.54) | 20.35 (8.53-32.16) | 0.85 (0.71 to 0.98) |
| Niue | 0.80 (0.44-1.35) | 1.34 (0.71-2.37) | 1.74 (1.66 to 1.82) | 18.74 (10.09-31.67) | 21.24 (11.66-36.09) | 0.43 (0.38 to 0.48) |
| North Macedonia | 0.32 (0.21-0.47) | 0.52 (0.30-0.80) | 2.04 (1.62 to 2.45) | 6.38 (4.54-9.24) | 5.39 (3.29-8.20) | -0.53 (-0.81 to -0.25) |
| Northern Mariana Islands | 0.94 (0.49-1.67) | 1.53 (0.90-2.54) | 1.41 (1.01 to 1.82) | 15.51 (8.50-27.59) | 18.89 (11.62-30.16) | 0.78 (0.44 to 1.11) |
| Norway | 1.14 (0.89-1.43) | 1.41 (1.04-1.87) | 0.58 (0.01 to 1.15) | 9.19 (8.32-10.07) | 4.54 (3.84-5.35) | -2.59 (-2.92 to -2.27) |
| Oman | 0.14 (0.07-0.25) | 0.32 (0.15-0.60) | 3.07 (2.74 to 3.41) | 1.48 (0.75-2.67) | 1.36 (0.69-2.60) | 0.15 (-0.12 to 0.42) |
| Pakistan | 0.14 (0.09-0.21) | 0.25 (0.15-0.38) | 1.66 (1.58 to 1.74) | 4.46 (2.85-6.73) | 5.58 (3.51-8.36) | 0.54 (0.39 to 0.69) |
| Palau | 1.55 (0.86-2.70) | 2.19 (1.18-3.75) | 1.04 (0.96 to 1.12) | 35.09 (19.56-61.39) | 35.32 (19.25-60.22) | 0.03 (0 to 0.07) |
| Palestine | 0.75 (0.41-1.29) | 1.43 (0.77-2.41) | 1.97 (1.84 to 2.1) | 9.25 (5.18-15.64) | 8.77 (5.24-14.70) | -0.3 (-0.38 to -0.22) |
| Panama | 1.19 (0.82-1.69) | 2.83 (1.72-4.28) | 2.59 (2.08 to 3.11) | 8.91 (6.84-11.37) | 11.70 (7.93-16.45) | 0.87 (0.38 to 1.36) |
| Papua New Guinea | 0.42 (0.19-0.81) | 0.63 (0.27-1.18) | 1.28 (1.17 to 1.39) | 12.51 (5.64-23.98) | 16.32 (6.73-30.88) | 0.94 (0.85 to 1.04) |
| Paraguay | 0.51 (0.30-0.85) | 1.16 (0.64-1.94) | 2.62 (2.33 to 2.91) | 9.02 (5.48-14.75) | 12.79 (7.36-21.38) | 1.14 (0.84 to 1.44) |
| Peru | 0.58 (0.36-0.90) | 1.26 (0.70-2.16) | 2.55 (2.4 to 2.7) | 12.58 (7.84-19.23) | 10.79 (6.37-17.93) | -0.6 (-0.71 to -0.49) |
| Philippines | 0.47 (0.32-0.63) | 0.63 (0.42-0.88) | 0.67 (0.55 to 0.78) | 11.46 (7.84-14.43) | 11.89 (8.17-15.76) | -0.03 (-0.11 to 0.04) |
| Poland | 0.51 (0.44-0.60) | 0.89 (0.68-1.12) | 2.15 (1.84 to 2.46) | 11.74 (11.04-12.49) | 9.62 (8.48-10.79) | -0.68 (-1.04 to -0.32) |
| Portugal | 1.34 (0.93-1.88) | 2.10 (1.42-3.14) | 1.5 (0.96 to 2.05) | 11.29 (8.70-14.60) | 6.69 (4.90-8.85) | -1.83 (-2.14 to -1.51) |
| Puerto Rico | 2.07 (1.44-2.88) | 3.13 (1.99-4.68) | 1.03 (0.69 to 1.37) | 18.21 (13.72-23.47) | 15.13 (10.48-21.10) | -1.02 (-1.28 to -0.77) |
| Qatar | 0.74 (0.37-1.31) | 1.81 (0.90-3.40) | 3.17 (2.89 to 3.46) | 6.79 (3.48-11.71) | 5.96 (3.05-10.69) | -0.44 (-0.68 to -0.2) |
| Republic of Korea | 0.13 (0.07-0.21) | 0.33 (0.19-0.55) | 3.25 (2.83 to 3.67) | 3.43 (1.90-5.30) | 2.21 (1.29-3.58) | -1.68 (-1.78 to -1.58) |
| Republic of Moldova | 0.61 (0.47-0.77) | 1.16 (0.86-1.53) | 2.72 (2.43 to 3.01) | 9.21 (7.90-10.61) | 10.64 (8.80-12.50) | 1.1 (0.81 to 1.39) |
| Romania | 0.36 (0.27-0.48) | 1.01 (0.70-1.41) | 2.95 (2.61 to 3.29) | 7.17 (5.64-9.14) | 8.84 (6.77-11.50) | -0.02 (-0.33 to 0.29) |
| Russian Federation | 1.14 (1.06-1.23) | 2.01 (1.74-2.24) | 1.78 (1.55 to 2.02) | 12.15 (11.28-13.05) | 11.83 (10.41-13.19) | -0.51 (-0.82 to -0.19) |
| Rwanda | 0.48 (0.19-0.87) | 0.60 (0.28-1.11) | 0.18 (-0.03 to 0.39) | 16.87 (6.70-30.86) | 14.08 (6.66-26.41) | -1.23 (-1.44 to -1.01) |
| Saint Kitts and Nevis | 2.09 (1.62-2.63) | 2.88 (1.85-4.22) | 0.31 (-0.02 to 0.65) | 38.35 (32.27-45.05) | 29.22 (19.92-41.11) | -1.62 (-2.02 to -1.21) |
| Saint Lucia | 2.42 (1.84-3.14) | 4.01 (2.88-5.52) | 1.31 (0.97 to 1.66) | 37.17 (31.48-43.37) | 39.52 (30.74-50.32) | -0.04 (-0.27 to 0.18) |
| Saint Vincent and the Grenadines | 2.23 (1.70-2.94) | 4.15 (3.07-5.54) | 1.77 (1.54 to 2.01) | 33.81 (28.03-40.53) | 47.19 (38.30-57.18) | 0.9 (0.7 to 1.11) |
| Samoa | 0.33 (0.17-0.58) | 0.48 (0.25-0.84) | 1.19 (1.14 to 1.25) | 7.97 (4.21-13.79) | 8.25 (4.24-14.60) | 0.2 (0.07 to 0.33) |
| San Marino | 1.03 (0.60-1.70) | 1.10 (0.48-2.08) | 1.11 (0.53 to 1.69) | 6.42 (3.79-10.08) | 3.88 (1.76-7.34) | -0.82 (-1.18 to -0.46) |
| Sao Tome and Principe | 0.26 (0.15-0.40) | 0.58 (0.32-0.97) | 2.74 (2.67 to 2.81) | 7.48 (4.49-11.70) | 10.57 (5.97-17.30) | 1.11 (0.99 to 1.23) |
| Saudi Arabia | 0.15 (0.06-0.35) | 0.60 (0.25-1.43) | 4.92 (4.65 to 5.2) | 1.71 (0.74-3.98) | 2.46 (1.06-5.84) | 1.36 (1.06 to 1.66) |
| Senegal | 0.31 (0.18-0.50) | 0.79 (0.39-1.43) | 3.42 (3.2 to 3.64) | 9.67 (5.78-15.26) | 17.92 (8.73-31.34) | 2.42 (2.21 to 2.64) |
| Serbia | 0.45 (0.27-0.73) | 0.76 (0.42-1.22) | 1.63 (1.46 to 1.8) | 7.76 (4.77-12.28) | 6.35 (3.79-9.77) | -0.98 (-1.19 to -0.77) |
| Seychelles | 1.36 (0.77-2.35) | 2.63 (1.51-4.36) | 2.14 (1.76 to 2.52) | 30.25 (17.34-50.68) | 35.27 (21.14-57.89) | 0.48 (0.18 to 0.78) |
| Sierra Leone | 0.25 (0.14-0.40) | 0.56 (0.25-1.05) | 3.15 (2.94 to 3.36) | 7.83 (4.61-12.46) | 13.69 (6.22-24.71) | 2.29 (2.09 to 2.48) |
| Singapore | 0.13 (0.09-0.18) | 0.25 (0.16-0.36) | 1.67 (1.27 to 2.07) | 2.66 (2.03-3.44) | 1.81 (1.32-2.44) | -2.01 (-2.41 to -1.6) |
| Slovakia | 0.71 (0.44-1.11) | 1.00 (0.56-1.65) | 1.3 (1.06 to 1.54) | 10.48 (6.77-16.00) | 8.17 (4.86-13.14) | -0.75 (-0.93 to -0.58) |
| Slovenia | 1.28 (0.91-1.74) | 1.97 (1.26-2.91) | 1.2 (0.84 to 1.56) | 11.26 (8.79-14.17) | 7.58 (5.49-10.38) | -1.86 (-2.21 to -1.51) |
| Solomon Islands | 0.52 (0.26-0.92) | 0.86 (0.46-1.46) | 1.74 (1.68 to 1.8) | 16.26 (8.43-28.59) | 22.59 (12.21-40.52) | 1.17 (1.11 to 1.23) |
| Somalia | 0.37 (0.14-0.76) | 0.36 (0.15-0.74) | -0.27 (-0.37 to -0.17) | 12.97 (4.94-26.51) | 11.27 (4.66-23.38) | -0.67 (-0.77 to -0.57) |
| South Africa | 0.87 (0.66-1.17) | 1.51 (1.09-2.41) | 1.96 (1.84 to 2.08) | 22.80 (17.01-30.55) | 26.15 (18.79-40.92) | 0.49 (0.37 to 0.6) |
| South Sudan | 0.37 (0.15-0.71) | 0.47 (0.22-0.83) | 0.63 (0.48 to 0.79) | 11.89 (4.72-22.71) | 11.90 (5.56-22.01) | -0.17 (-0.33 to -0.01) |
| Spain | 0.97 (0.69-1.33) | 1.21 (0.82-1.75) | 0.58 (0.14 to 1.02) | 9.15 (7.21-11.37) | 4.41 (3.31-5.84) | -2.62 (-2.8 to -2.44) |
| Sri Lanka | 0.22 (0.13-0.35) | 0.36 (0.18-0.63) | 1.41 (1.19 to 1.63) | 4.82 (2.83-7.77) | 3.58 (1.84-6.04) | -1.41 (-1.7 to -1.12) |
| Sudan | 0.23 (0.12-0.41) | 0.58 (0.26-1.14) | 3.08 (2.96 to 3.19) | 4.66 (2.44-8.11) | 5.94 (2.61-11.48) | 0.91 (0.85 to 0.96) |
| Suriname | 1.00 (0.63-1.54) | 1.62 (0.88-2.73) | 1.55 (1.36 to 1.74) | 18.36 (11.97-27.54) | 22.29 (12.50-37.50) | 0.58 (0.39 to 0.78) |
| Sweden | 1.04 (0.78-1.34) | 1.16 (0.79-1.63) | 0.82 (0.08 to 1.56) | 7.53 (6.15-9.13) | 3.77 (2.78-5.04) | -1.86 (-2.31 to -1.42) |
| Switzerland | 1.56 (1.11-2.18) | 1.42 (0.94-2.04) | -0.26 (-0.69 to 0.17) | 9.78 (7.61-12.41) | 4.79 (3.43-6.52) | -2.32 (-2.49 to -2.15) |
| Syrian Arab Republic | 0.57 (0.28-0.90) | 1.22 (0.64-1.99) | 2.29 (2.13 to 2.45) | 7.43 (3.78-11.37) | 6.85 (3.56-10.46) | -0.57 (-0.76 to -0.38) |
| Taiwan (Province of China) | 0.54 (0.38-0.74) | 1.49 (1.00-2.15) | 2.76 (2.26 to 3.26) | 6.27 (4.83-7.99) | 8.58 (6.43-11.33) | 0.4 (-0.09 to 0.89) |
| Tajikistan | 0.38 (0.24-0.58) | 0.47 (0.21-0.90) | 0.42 (0.08 to 0.76) | 11.28 (6.98-17.23) | 11.64 (5.22-22.73) | -0.19 (-0.46 to 0.08) |
| Thailand | 0.42 (0.18-0.72) | 1.29 (0.37-2.44) | 3.7 (3.48 to 3.92) | 8.26 (3.57-14.27) | 12.39 (3.65-23.14) | 1.16 (0.98 to 1.34) |
| Timor-Leste | 0.14 (0.06-0.26) | 0.25 (0.12-0.46) | 1.98 (1.77 to 2.19) | 4.56 (2.08-8.41) | 5.81 (2.83-10.43) | 0.74 (0.53 to 0.95) |
| Togo | 0.30 (0.18-0.46) | 0.74 (0.34-1.31) | 3.18 (3.06 to 3.29) | 9.24 (5.63-14.17) | 17.13 (7.88-30.26) | 2.22 (2.07 to 2.37) |
| Tokelau | 0.65 (0.32-1.18) | 1.27 (0.67-2.21) | 2.07 (2 to 2.14) | 17.88 (8.69-32.55) | 21.70 (11.97-38.11) | 0.54 (0.48 to 0.6) |
| Tonga | 0.96 (0.56-1.56) | 1.56 (0.87-2.61) | 1.53 (1.46 to 1.6) | 22.09 (13.29-35.32) | 26.86 (15.36-43.85) | 0.67 (0.6 to 0.74) |
| Trinidad and Tobago | 1.65 (1.30-2.10) | 3.37 (2.14-4.95) | 2.09 (1.84 to 2.34) | 24.57 (21.03-28.64) | 33.25 (23.33-45.90) | 0.55 (0.27 to 0.84) |
| Tunisia | 0.22 (0.12-0.37) | 0.56 (0.29-0.97) | 3.04 (2.97 to 3.12) | 2.22 (1.28-3.65) | 2.64 (1.41-4.46) | 0.45 (0.39 to 0.51) |
| Turkey | 0.69 (0.36-1.20) | 1.65 (0.92-2.81) | 3.18 (2.94 to 3.43) | 9.69 (5.34-16.39) | 7.86 (4.57-12.88) | -0.69 (-0.88 to -0.49) |
| Turkmenistan | 0.35 (0.30-0.41) | 0.84 (0.63-1.10) | 3.75 (3.46 to 4.04) | 9.99 (9.03-11.00) | 17.78 (14.17-22.49) | 2.76 (2.44 to 3.08) |
| Tuvalu | 0.56 (0.31-0.91) | 0.98 (0.53-1.69) | 1.86 (1.8 to 1.92) | 17.38 (9.52-28.90) | 21.06 (11.63-34.84) | 0.69 (0.65 to 0.73) |
| Uganda | 0.78 (0.44-1.25) | 1.28 (0.70-2.19) | 1.09 (0.92 to 1.26) | 24.37 (14.28-39.59) | 29.80 (16.52-51.45) | 0.05 (-0.17 to 0.28) |
| Ukraine | 1.14 (0.89-1.45) | 1.17 (0.68-1.79) | 0.16 (-0.56 to 0.88) | 25.21 (19.73-31.58) | 20.51 (12.36-31.79) | -0.68 (-1.43 to 0.08) |
| United Arab Emirates | 0.64 (0.33-1.15) | 0.92 (0.44-1.75) | 1.62 (1.34 to 1.9) | 8.20 (4.42-14.68) | 5.93 (2.87-10.97) | -0.61 (-0.91 to -0.31) |
| United Kingdom | 1.04 (0.96-1.12) | 1.93 (1.76-2.11) | 2.23 (1.95 to 2.51) | 7.92 (7.56-8.28) | 7.17 (6.65-7.83) | -0.33 (-0.45 to -0.22) |
| United Republic of Tanzania | 0.48 (0.18-0.94) | 0.60 (0.25-1.15) | 0.5 (0.43 to 0.58) | 15.09 (5.55-29.07) | 13.93 (5.97-26.52) | -0.46 (-0.53 to -0.39) |
| United States of America | 2.80 (2.63-2.98) | 3.98 (3.68-4.32) | 0.56 (0.15 to 0.97) | 10.41 (9.71-11.26) | 9.63 (8.62-10.72) | -0.75 (-0.96 to -0.54) |
| United States Virgin Islands | 2.49 (1.31-4.22) | 3.98 (1.81-7.89) | 1.68 (1.53 to 1.84) | 28.26 (15.63-47.47) | 34.55 (16.44-68.39) | 1.13 (0.99 to 1.27) |
| Uruguay | 0.82 (0.59-1.11) | 1.60 (1.09-2.25) | 1.78 (1.44 to 2.13) | 14.43 (11.01-18.77) | 14.20 (10.40-18.75) | -0.48 (-0.7 to -0.27) |
| Uzbekistan | 0.26 (0.19-0.36) | 0.39 (0.26-0.57) | 1.72 (1.18 to 2.26) | 6.95 (5.13-9.06) | 7.79 (5.40-10.94) | 0.65 (0.24 to 1.07) |
| Vanuatu | 0.51 (0.28-0.87) | 0.78 (0.43-1.28) | 1.3 (1.26 to 1.34) | 15.09 (8.05-25.72) | 20.20 (11.19-33.64) | 0.9 (0.85 to 0.94) |
| Venezuela (Bolivarian Republic of) | 1.35 (1.03-1.77) | 3.46 (2.18-5.18) | 2.5 (2.02 to 2.98) | 13.98 (11.96-16.19) | 21.01 (14.47-29.11) | 0.72 (0.4 to 1.04) |
| Viet Nam | 0.05 (0.03-0.08) | 0.12 (0.07-0.21) | 3.07 (2.99 to 3.15) | 1.21 (0.70-1.96) | 1.38 (0.77-2.31) | 0.54 (0.48 to 0.61) |
| Yemen | 0.23 (0.10-0.41) | 0.46 (0.19-0.87) | 2.64 (2.45 to 2.83) | 4.42 (2.10-7.84) | 5.65 (2.36-10.58) | 0.89 (0.78 to 1.01) |
| Zambia | 0.40 (0.17-0.72) | 1.48 (0.36-3.17) | 5.28 (4.53 to 6.04) | 13.13 (5.68-23.69) | 35.08 (8.53-73.41) | 4.11 (3.47 to 4.76) |
| Zimbabwe | 0.72 (0.43-1.15) | 1.50 (0.74-2.59) | 2.16 (1.56 to 2.76) | 20.35 (11.93-32.54) | 38.85 (18.81-69.93) | 2.17 (1.63 to 2.72) |

**
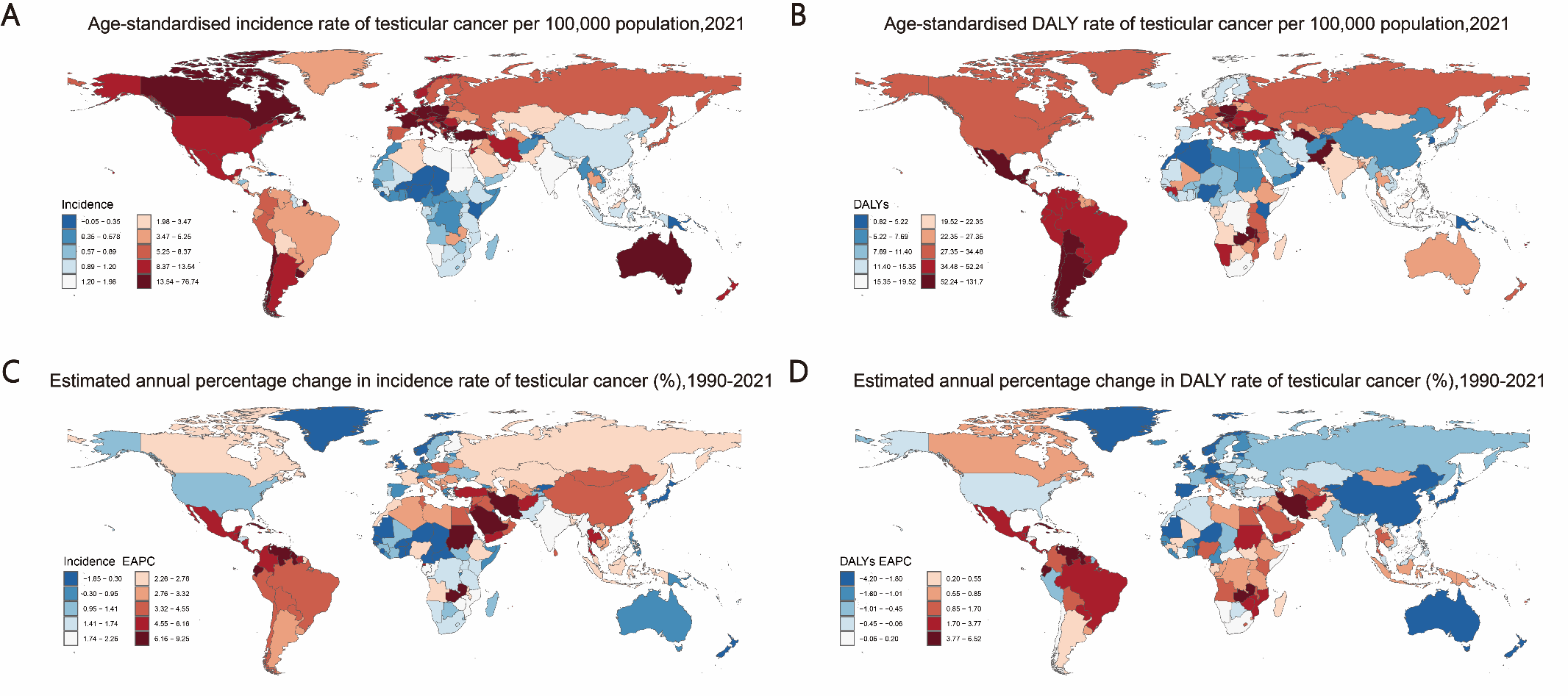
**

**Figure S1. Age-standardized incidence and DALY rates in 2021, and their estimated annual percentage changes from 1990 to 2021 for testicular cancer, by country**

Age-standardized rate (A) and estimated annual percentage change (B) of the incidence of testicular cancer. Age-standardized rate (C) and estimated annual percentage change (D) of the DALYs of testicular cancer. DALY=disability-adjusted life-years.


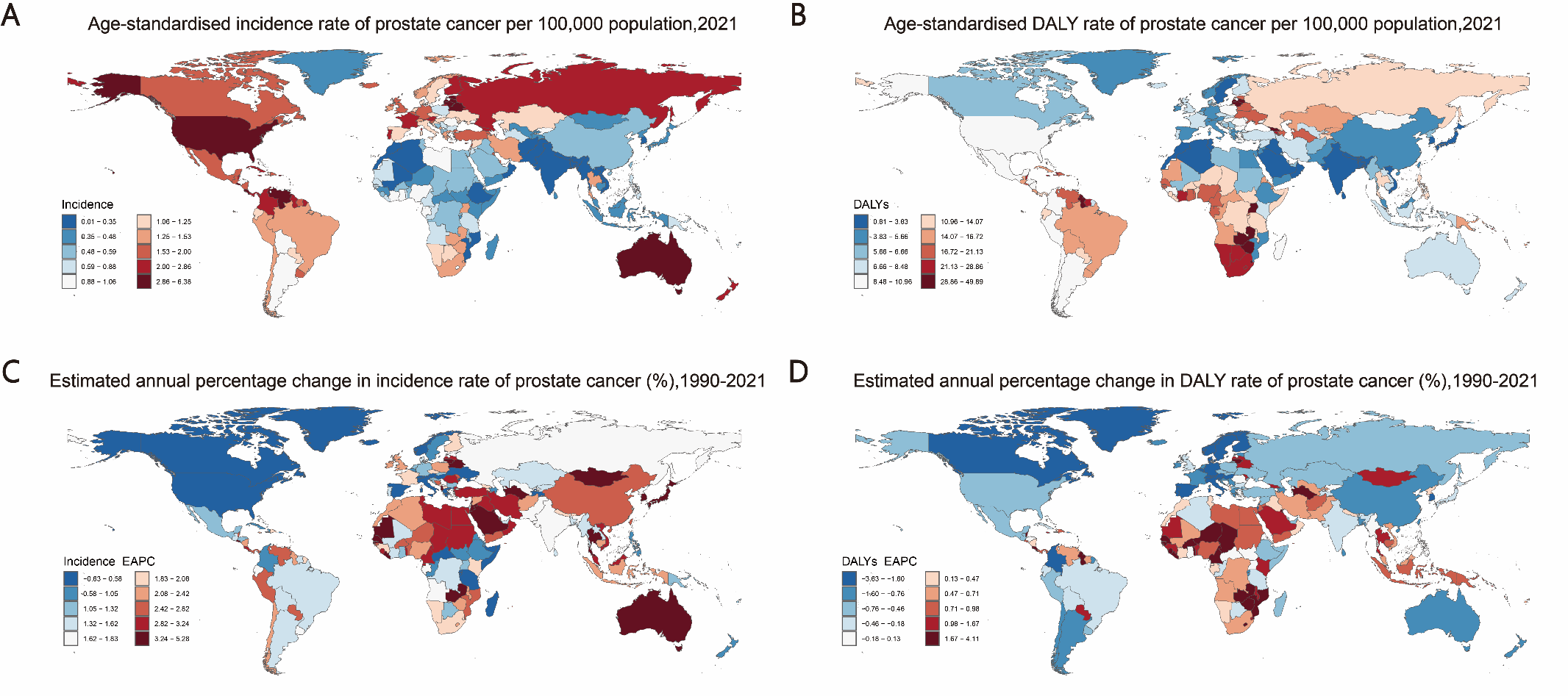


**Figure S2. Age-standardized incidence and DALY rates in 2021, and their estimated annual percentage changes from 1990 to 2021 for prostate cancer, by country**

Age-standardized rate (A) and estimated annual percentage change (B) of the incidence of prostate cancer. Age-standardized rate (C) and estimated annual percentage change (D) of the DALYs of prostate cancer. DALY=disability-adjusted life-years.


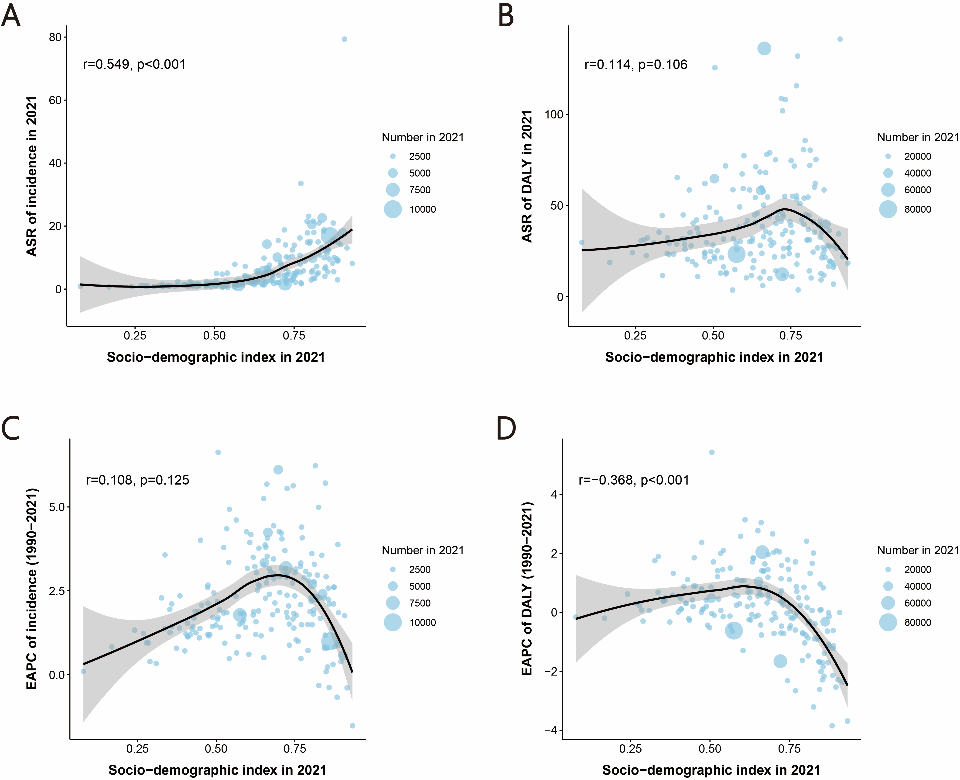


**Figure S3. Age-standardized rates of incidence and DALYs in 2021, and their estimated annual percentage change (1990–2021) of overall male cancers, globally and for the 204 countries and territories, by SDI in 2021**

Age-standardized rate (A) and estimated annual percentage change (C) of incidence of overall male cancers, by SDI. Age-standardized rate (B) and estimated annual percentage change (D) of DALYs of overall male cancers, by SDI. Expected values, based on SDI and disease rates in all countries and territories, are shown as a solid line; expected values are based on a calculation accounting for the SDI and disease rates across all countries and territories. 204 circles are plotted for each country and show the observed age-standardized incidence or DALY rates in 2021. The size of the circle is increased with the cases of male cancers. The shaded area indicates the 95% CI of the expected values. Countries and territories above the solid line represent a higher-than-expected burden, and countries and territories below the line show a lower-than-expected burden. Male cancers include testicular cancer, and prostate cancer. ASR=age-standardized rate. DALY=disability-adjusted life-years. SDI=socio-demographic index. EAPC=estimated annual percentage change. We regarded p<0.05 as statistically significant.


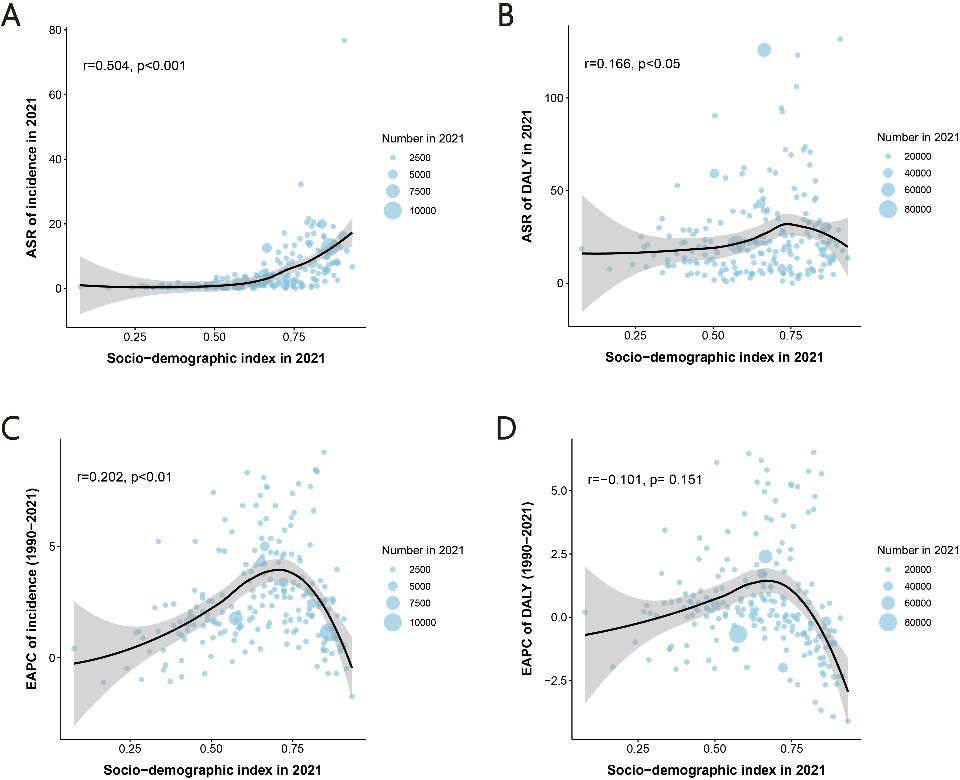


**Figure S4. Age-standardized rates of incidence and DALYs in 2021, and their estimated annual percentage change (1990–2021) of testicular cancer, globally and for the 204 countries and territories, by SDI in 2021**

Age-standardized rate (A) and estimated annual percentage change (C) of incidence of testicular cancer, by SDI. Age-standardized rate (B) and estimated annual percentage change (D) of DALYs of testicular cancer, by SDI. ASR=age-standardized rate. DALY=disability-adjusted life-years. SDI=socio-demographic index. EAPC=estimated annual percentage change. We regarded p<0.05 as statistically significant.


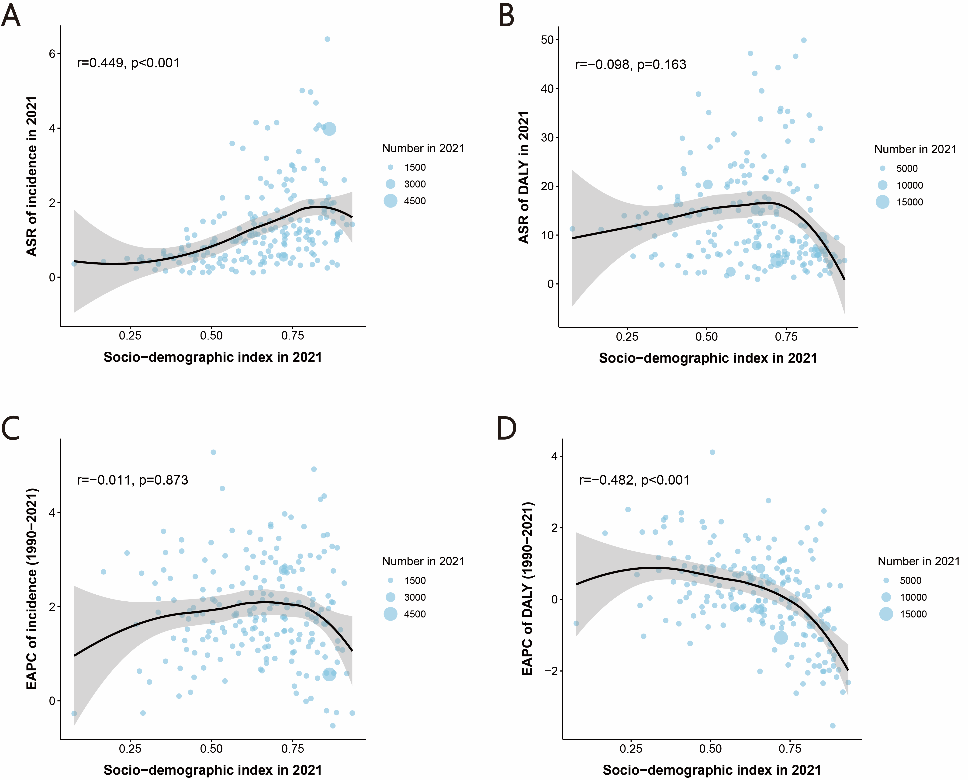


**Figure S5. Age-standardized rates of incidence and DALYs in 2021, and their estimated annual percentage change (1990–2021) of prostate cancer, globally and for the 204 countries and territories, by SDI in 2021**

Age-standardized rate (A) and estimated annual percentage change (C) of incidence of prostate cancer, by SDI. Age-standardized rate (B) and estimated annual percentage change (D) of DALYs of prostate cancer, by SDI. ASR=age-standardized rate. DALY=disability-adjusted life-years. SDI=socio-demographic index. EAPC=estimated annual percentage change. We regarded p<0.05 as statistically significant.
