# Supplementary material for: Global trends in testicular and prostate cancer among adolescents and young adult males aged 15–49 years, 1990–2021: insights from the GBD study
Source: Sci Rep. 2025 Jul 2;15:23388. doi: 10.1038/s41598-025-07361-3 (PMC12222491; doi:10.1038/s41598-025-07361-3)
Supplement: Supplementary file 2 — Supplementary Material 2 [file 41598_2025_7361_MOESM2_ESM.docx]

Supplementary File 1. Data Sources and Quality Assurance in the GBD 2021 Database

1. Overview of Data Sources

The Global Burden of Disease (GBD) 2021 study synthesizes data from multiple sources to generate robust and globally representative estimates. Key data sources include:

- Vital registration systems (e.g., national death certificates)
- Population-based cancer registries (e.g., IARC, SEER, CI5)
- Household surveys (e.g., DHS, MICS)
- Health administrative records
- Peer-reviewed scientific literature
- Verbal autopsy data in settings lacking reliable civil registration systems

2. Quality Control and Estimation Methods

To mitigate data gaps and heterogeneity across countries and time periods, GBD employs several advanced modeling techniques:

- DisMod-MR 2.1: A Bayesian meta-regression framework that ensures internal consistency across epidemiological parameters (incidence, prevalence, remission, and mortality).
- CODEm (Cause of Death Ensemble Modeling): A modeling system that tests multiple algorithms and covariates to estimate cause-specific mortality.
- Spatiotemporal Gaussian Process Regression (ST-GPR): Applied to interpolate data-sparse regions by leveraging temporal and spatial correlations.
- Data harmonization: Includes smoothing, calibration, and adjustment for underreporting, misclassification, and missing data using standardized algorithms.

3. Data Quality Assessment and Bias Adjustment

All data sources are graded based on completeness, accuracy, and representativeness. Adjustments are made for potential biases such as:

- Under- and overdiagnosis
- Reporting delays
- Systematic data gaps, especially in low- and middle-income settings
- Estimates are reported with 95% uncertainty intervals (UIs) to reflect data reliability and model uncertainty.

4. Cancer-Specific Procedures

For testicular and prostate cancers, GBD prioritizes data from high-quality cancer registries (e.g., CI5, SEER, Nordcan). When registry data are unavailable, estimates are derived using model-based extrapolation informed by:

- Sociodemographic Index (SDI)
- Age structure
- Health system access
- Regional cancer patterns
- Standardized ICD-coded mortality data and ICD-O-coded cancer incidence data are utilized where available.

5. Validation and Transparency

GBD estimates undergo out-of-sample validation and are benchmarked against independent data sources (e.g., WHO, IHME, GLOBOCAN).

All data inputs, methodological details, and modeling code are publicly available via the Global Health Data Exchange (GHDx): http://ghdx.healthdata.org.
